# Prospects for Declarative Mathematical Modeling of Complex Biological Systems

**DOI:** 10.1007/s11538-019-00628-7

**Published:** 2019-06-07

**Authors:** Eric Mjolsness

**Affiliations:** Department of Computer Science, University of California, Irvine, CA 92697 USA

**Keywords:** Declarative modeling, Development, Multiscale modeling, Operator algebra, Semantics, Graph grammars, Graded graphs, Stratified graphs, Cell division, Cytoskeleton

## Abstract

Declarative modeling uses symbolic expressions to represent models. With such expressions, one can formalize high-level mathematical computations on models that would be difficult or impossible to perform directly on a lower-level simulation program, in a general-purpose programming language. Examples of such computations on models include model analysis, relatively general-purpose model reduction maps, and the initial phases of model implementation, all of which should preserve or approximate the mathematical semantics of a complex biological model. The potential advantages are particularly relevant in the case of developmental modeling, wherein complex spatial structures exhibit dynamics at molecular, cellular, and organogenic levels to relate genotype to multicellular phenotype. Multiscale modeling can benefit from both the expressive power of declarative modeling languages and the application of model reduction methods to link models across scale. Based on previous work, here we define declarative modeling of complex biological systems by defining the operator algebra semantics of an increasingly powerful series of declarative modeling languages including reaction-like dynamics of parameterized and extended objects; we define semantics-preserving implementation and semantics-approximating model reduction transformations; and we outline a “meta-hierarchy” for organizing declarative models and the mathematical methods that can fruitfully manipulate them.

**Electronic supplementary material:**

The online version of this article (10.1007/s11538-019-00628-7) contains supplementary material, which is available to authorized users.

## Introduction

Central to developmental biology is the genotype-to-phenotype map required to close the evolutionary loop implied by selection on heritable variation. However, relating genotype to phenotype in a multicellular organism is an intrinsically multiscale and (therefore) complex modeling endeavor. Partial automation has the potential to tame the complexity for human scientists, provided it can address highly heterogeneous mathematical dynamics including stochastic reaction networks, dynamic spatial structures at molecular and cellular scales, and partial differential equations (PDEs) governing both pattern formation and dynamic geometry within dynamic topology. Here, we outline such a mathematical modeling framework, founded on the unifying idea of rewrite rules denoting operators in an operator algebra. The rewrite rules make this framework *declarative*: capable of expressing mathematical ideas at a high level in a symbolically and computationally manipulable form.

To this end, we propose and discuss an informal definition of declarative modeling in general and provide as examples a collection of specific mathematical constructions of processes and extended objects for use in declarative models of complex biological systems and their processing by computer. Such processing can be symbolic and/or numerical, including for example model reduction by coupled symbolic and machine learning methods. The resulting apparatus is intended for semiautomatic synthesis and analysis of biological models, a computational domain which must typically deal with substantial intrinsic complexity in the subject modeled.

The necessity for such automation is strongest for the most complex biological models, notably those required for developmental modeling. Examples of such dynamical spatial systems in development include plant cell division under the influence of dynamic microtubules in the pre-prophase band; neurite branching and somal translocation dependent on dynamic cytoskeleton in mammalian brain development; mitochondrial fission and fusion; plant organogenesis in shoot (shoot apical meristem or SAM) and root (lateral root initiation); topological changes in close-packed 2D tissues (e.g., fly wing disk) in response to cell division and convergent extension; neural tube closure; branching morphogenesis in vascular tissues; and many others. In all these cases, the spatial dynamics involves nontrivial changes in geometry and/or topology of extended biological objects; we must be able to represent such dynamics mathematically and computationally. Our main examples will be networks of dynamically interconnected cells and dynamically interconnected segments of cytoskeleton within a cell.

This paper is organized as follows. We will define declarative models in Sect. 2 (informally in general but formally in particular cases) and survey a series of declarative quantitative modeling languages of increasing expressive power for biochemical and biological modeling as exemplified by coarse-scale models of multicellular tissues with cell division and by cytoskeletal dynamics; also we will describe the “operator algebra” mathematical semantics for these languages and the utility of structure-respecting maps among these mathematical entities. We will generalize the declarative modeling language/operator algebra semantics approach to encompass extended objects in Sect. 3, by way of spatially embedded discrete graph structures (including dimension and refinement level indices) and their continuum limits; dynamics on such structures including PDEs; and dynamics by which such structures can change including graph rewrite syntactic rules under a novel operator algebra semantics. We will discuss progress toward a general method for nonlinear model reduction (usually across scales) by machine learning in Sect. 4 including an application of variational calculus generalized to a higher level. In Sect. 5, we will propose the elements of a larger-scale mathematically based “meta-hierarchy” for organizing many biological models and modeling methods, enabled by the declarative approach to modeling and by structure-respecting maps among declarative languages and their mathematical semantics.

Much of this paper reviews previous work, extending it (e.g., with the multiscale “graded graph” constructions of Sect. 3, their dynamics of Eq. (17), and graph rewrite rule operator products defined by Propositions 1 and 2 in Sect. 3.2.2) and setting it in a broader context. The aim of this paper is not to provide a balanced summary of work in the field; instead it is aimed mainly at outlining mathematically the possibilities of particular directions for future development.

## Declarative Modeling

A distinction made in classical Artificial Intelligence (AI) by Winograd among others is that between *declarative* and *procedural* representations of knowledge; this is the AI version of a philosophical distinction between “knowing that” and “knowing how,” as it pertains to knowledge expressed in a formal language that can be used to program intelligent systems (Winograd 1975). Generic advantages for declarative knowledge identified by Winograd include its greater flexibility, compactness, understandability, and communicability compared to procedural knowledge; these are virtues we seek for complex biological modeling. On the other hand, declarative knowledge may be incomplete, as it omits for example “heuristic” knowledge of domain-specific strategies for action (terminology surrounding the “declarative/procedural” distinction is discussed briefly in Supplementary Material Section 7.1).

**Fig. 1:**
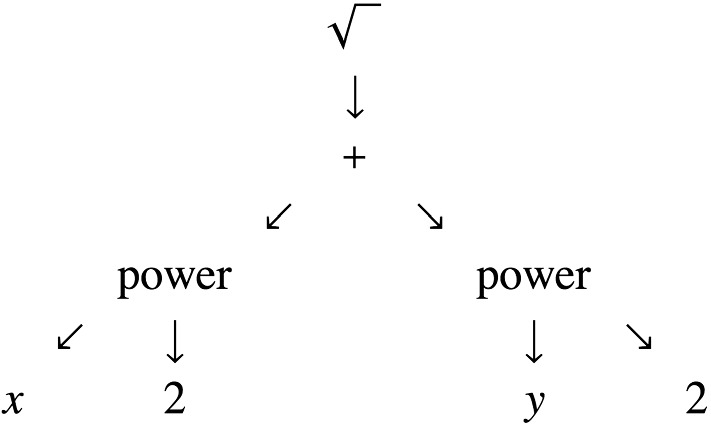
Schematic view of an abstract syntax tree for the algebraic expression $\sqrt{x^2+y^2}$. It can be transformed in many ways. For example, it could be numerically evaluated at $x=5,y=12$ by successive local transformations of ASTs. AST transformations are used in compilers for programming languages, in computer algebra systems, and in recent work on “symbolic regression” in machine learning

An example of a formalizable, declarative language for modeling biology is a collection of partial differential equations in which the variables represent local concentrations of molecular species and the spatial differential operators are all diffusion operators $\nabla _{{{\varvec{x}}}}\cdot (D_\alpha ({{\varvec{x}}}) \nabla _{{\varvec{x}}}$). Such a deterministic reaction/diffusion model can be represented in a computer by one or more abstract syntax trees (ASTs) including nodes for variable names, arithmetic operations, spatial differential operators such as the Laplacian, the first-order time derivative operator, equality constraints including boundary conditions, and possibly function definitions (a simple AST is shown in Fig. 1). Such an AST can be used to denote a reaction–diffusion model as a data object that can be manipulated by computer algebra. It can also under some conditions be transformed symbolically, for example by separation of time scales replacing a subset of ordinary differential equations (without spatial derivatives) by function definitions of algebraic rate laws to be invoked in the differential equations for the remaining variables (e.g., Ermentrout 2004). The language is “declarative” from a computational point of view because it does not specify any algorithmic details for numerically solving the dynamical systems specified. Implemented examples will be discussed in Sect. 3.

We generalize from this example. A formal language should have a “semantics” map ${\varPsi }$, giving a mathematical meaning to some defined set of valid expressions in the language. For a modeling language, each valid model declaration *M* should correspond to an instance ${\varPsi }(M)$ in some space *S* of “dynamical systems” interpreted broadly, so that such systems may be stochastic and/or infinite-dimensional. If some of the semantically meaningful model expressions are composed of meaningful sub-expressions, and their semantics can be combined in a corresponding way, then the semantics is “compositional”; composition commutes with the semantic map. Likewise for valid transformations of model declarations, one would like the semantics before and after transformation to yield either the same mathematical meaning (equivalent dynamics), or two meanings that are related somehow, for example, by approximate equality under some conditions on parameters that may be partially known by proof and/or numerical experiment.

So in the context of modeling languages in general and biological modeling languages in particular, some key advantages of the declarative language style are captured by the following informal (but perhaps formalizable) definition:

A *declarative modeling languageL* is a formal language together with (a) compositional semantics ${\varPsi }:L \rightarrow S$ that maps all syntactically valid models *M* in *L* into some space *S* of dynamical systems, and (b) conditionally valid or conditionally approximately valid families of *transformations* on model-denoting AST expressions in the modeling language *L*. These AST transformations can be expressed formally in some computable meta-language, though the meta-language need not itself be a declarative modeling language.

Under this informal definition, the utility of a declarative modeling language would depend on its expressive power, addressed in Sects. 2.1–2.3 and 3, and on the range, value, and reliability of the model-to-model transformations that can be constructed for it, to be discussed in Sects. 3 (implementation), 4 (model reduction), and 5 (wider prospects). Multiscale modeling benefits from both the expressive power (e.g., representing cellular and molecular processes in the same model) and model reduction (finding key coarse-scale variables and dynamics to approximate fine-scale ones) elements of this agenda.

Although it is plausible that “one can’t proceed from the informal to the formal by formal means” (Perlis 1982), so that the task of formalizing a complex biological system to create models must begin informally, we will nevertheless try to be *systematic* about this task by building the semantic map ${\varPsi }$ up out of “processes” and “objects” of increasing generality and structure. These processes and objects are represented by “expressions” in a language, and mapped to mathematical objects that together define a model. So the map ${\varPsi }$ will be concerned with expressions, processes, and objects.

We show how to define a series of simple quantitative modeling languages of increasing expressive power, accompanied by semantic maps ${\varPsi }(M)$ to operator algebras. To do this, we need some idea concerning how to represent elementary biological processes with syntactic expressions that can include numerical quantities. One such idea begins with chemical reaction notation.

### Pure Reaction Rules

In biology, generalized versions of *rewrite rules* naturally specify biochemical processes in a declarative modeling language, as well as model transformations in a meta-language. The most straightforward example is chemical reaction notation. As in Mjolsness (2010), we could use chemical “addition” notation:

1 $$\begin{aligned} \Bigg ( \sum \limits _{\alpha =1}^{A_{\max }}m_{\alpha }^{\left( r\right) } A_{\alpha } \Bigg ) \ \ \overset{k_{\left( r\right) }}{\longrightarrow }\ \ \Bigg ( \sum \limits _{\beta =1}^{A_{\max }}n_{\beta }^{\left( r\right) } A_{\beta } \Bigg ) , \end{aligned}$$

where $m_{\alpha }^{\left( r\right) }$ and $n_{\beta }^{\left( r \right) }$ are nonnegative integer-valued stoichiometries for molecular species $A_{\alpha }$ indexed by $\alpha$ in reaction *r* with nonnegative reaction rate $k_{(r)}$, and we may omit summands with $m_{\alpha }^{( r) }=0$ or $n_{\beta }^{(r)}=0$. The left hand side (LHS) and right hand side (RHS) of the reaction arrow are just formal sums or equivalently ${ multisets}$ with nonnegative integer multiplicities of all possible reactants, defaulting to zero if a reactant is not mentioned:

2 $$\begin{aligned} \Big \{ m_{\alpha }^{\left( r\right) } A_{\alpha } \Big \}_* \ \ \overset{k_{\left( r\right) }}{\longrightarrow }\ \ \ \Big \{ n_{\beta }^{\left( r\right) } A_{\beta } \Big \}_*. \end{aligned}$$

Concisely, we can summarize reaction rule *r* as “${\text {LHS}_r \rightarrow \text {RHS}_r}$”. Either detailed syntax expresses the transformation of one multiset of symbols into another, with a numerical or symbolic quantitative reaction rate $k_{\left( r\right) }$. The syntax is easily encoded in an abstract syntax tree (AST).

An example AST *transformation* might be a meta-rule that reverses an arrow (Yosiphon 2009) and changes the name of the reaction rate to (for example) $k_{\left( r^{\prime }\right) }$, for a new reaction number $r^{\prime }$, allowing for the possibility of detailed balance to be satisfied in a collection of reactions. Many other reaction arrow types (e.g., substrate-enzyme-product) can then be defined by computable transformation to combinations of these elementary mass action reactions [e.g., Shapiro et al. (2003), Yang et al. (2005) and Shapiro et al. (2015) for many examples in a declarative biological modeling context], using either commercial (Wolfram Research 2017) or open-source (Joyner et al. 2012) computer algebra system software.

How can we define a compositional semantics for this reaction notation? Fortunately, the operator algebra formalism of quantum field theory can be adapted to model the case of ordinary (non-quantum) probabilities governed by the law of mass action in a Master Equation (Doi 1976a, b; Peliti 1985; Mattis and Glasser 1998; Mjolsness 2010) [and Morrison and Kinney (2016) for the equilibrium case]. As in physics, each operator algebra we deal with will be generated by a collection of elementary operators and their commutation relations, together with their closure under operator addition, operator multiplication, and scalar multiplication of operators by real numbers. In the present case, the generators are the identity operator together with, for each molecular species or other symbol type $\alpha$ in the reaction set, a creation operator ${\hat{a}}_\alpha$ and an annihilation operator $a_\alpha$ the commutation relations are $a_\alpha {\hat{a}}_\beta = {\hat{a}}_\beta a_\alpha - 2 \delta _{\alpha \beta } {\hat{a}}_\alpha a_\alpha + \delta _{\alpha \beta } I_{\alpha }$ as discussed in Supplementary Material Section 7.2.1. Then, the semantics ${\varPsi }(M)$ is determined by the creation/annihilation operator monomials

3 $$\begin{aligned} {{\hat{W}}}_{r} \equiv {\hat{W}}_{\text {LHS}_r \rightarrow \text {RHS}_r} \equiv k_{r} \left\{ \prod \limits _{\beta \in {\text {rhs}}( r) } ({{\hat{a}}}_{\beta })^{n_{\beta }^{\left( r\right) }} \right\} \left\{ \prod \limits _{ \alpha \in {\text {lhs}}( r) } (a_{\alpha })^{m_{\alpha }^{\left( r\right) }} \right\} \ \ \end{aligned}$$

which specifies the nonnegative flow of probability between states under each reaction *r*. The states are given by vectors $$|{{\varvec{n}}}\rangle = |[n_\alpha ]\rangle $$ of nonnegative integers $$n_\alpha $$ (distinguished from stoichiometry $$n_{\beta }^{\left( r\right) }$$ by its lack of a superscript), one for each molecular species. The full system state is a probability distribution $$p(|{{\varvec{n}}}\rangle )$$, on which operators act linearly. Here, $$ k_{r }$$ is conventional notation for a reaction rate; later we will call it $$ \rho _{r }$$ instead. The usual chemical law of mass action is encoded in the annihilation operators $$a_{\alpha }$$. Annihilation operator subscript $$\alpha $$ indexes the species present in the multiset on the left hand side (LHS) of Eq. (2); the corresponding operator is raised to the power of its multiplicity or ingoing stoichiometry $$m_{\alpha }^{\left( r\right) }$$ in reaction *r*, destroying that many particles of species $$\alpha $$ if they exist (and contributing zero probability if they don’t). Likewise creation operator $${{\hat{a}}}_{\beta }$$ has subscript $$\beta $$ indexing the species present in the multiset on the right hand side (RHS) of Eq. (2), and the operator is raised to the power of its outgoing stoichiometry $$n_{\beta }^{\left( r\right) }$$ which indicates how many particles of species $$\beta $$ are to be created. The “$${\hat{W}}_{\text {LHS}_r \rightarrow \text {RHS}_r}$$” notation is also used by Behr et al. (2016). Given Eq. (3), the actual *semantics* is then expressed by $${\varPsi }(M)=W(M)$$ where *W*(*M*) sums over all reactions *r* as in Eqs. (4) and (5).

Explicit expressions for the creation and annihilation operators used here, along with the algebra they satisfy, calculation of probability conservation operator *D*, and definition of equivalent models, are provided in Supplementary Material Section 7.2.1.

The model semantics built on Eq. (3) is *compositional* over processes, hence structure-preserving, because:

- The operators for multiple rules indexed by *r* in a ruleset map to an *operator sum*:
  4a $$\begin{aligned} W&= \sum _r W_r \; , \quad \text {(rule operators sum up), where} \end{aligned}$$
  4b $$\begin{aligned} \quad \quad W_r&\equiv {\hat{W}}_r - D_r , \quad \text{(rules } \text{ conserve } \text{ probability) } \end{aligned}$$
  4c $$\begin{aligned} D_r&\equiv \mathrm{diag}(\mathbf{1 } \cdot {\hat{W}}_r) \quad \text{(total } \text{ probability } \text{ outflow } \text{ per } \text{ state) } \end{aligned}$$
  that specifies the combined dynamics under the chemical master equation:
  5 $$\begin{aligned} {\dot{p}} = W \cdot p. \end{aligned}$$
  In Eq. (4), the first statement is ruleset compositionality, the second and third ensure conservation of probability. Equation (5) is the resulting Chemical Master Equation (CME) stochastic dynamical system for the evolving state probability $p({{\varvec{n}}})$. Also the semantics is compositional because:
- The multisets on the left hand side and right hand side of a rule each map to an *operator product* in normal form (including powers for repeated multiset elements) in Eq. (3). Each product consists of commuting operators so their order is arbitrary.

The default continuous-time semantics $W = {\varPsi }( M )$ is now be defined by Eq. (3) (the specific semantic map for each rule of a model *M*) and Eq. (4), in the context of Eq. (5). Thus, we have defined a “structure-respecting” mapping ${\varPsi }$ from pure (multiset-changing) rulesets (each rules weighted by a nonnegative reaction rate) to operator algebras; ${\varPsi }$ is (at least) a linear mapping of vector spaces.

The integer-valued index *r* we used to name the reactions is part of a meta-language for the present theorizing, and not part of the modeling language. One slightly confusing point is that these unordered collections of chemical reactions are expressions in the language, but they also have a form reminiscent of a grammar for *another language*—albeit a language of multisets representing the system state, rather than of strings or trees, and a language that may be entirely devoid of terminal symbols (which would represent inert products such as waste). The CME as semantics was suggested by Mjolsness and Yosiphon (2006); Mjolsness (2005) and by Cardelli (2008), though it can be regarded as implicit in the original Doi–Peliti formalism. There is also a projection from continuous-time (CME) semantics to discrete-time probabilistic semantics in the form of a Markov chain (Mjolsness and Yosiphon 2006).

From this operator algebra semantics and the time-ordered product expansion (TOPE) approach to Feynman path integrals (Mjolsness and Yosiphon 2006; Mjolsness 2005), one can derive valid exact stochastic simulation algorithms including the Gillespie stochastic simulation algorithm (SSA) and various generalizations, as detailed in Mjolsness (2013). Such algorithms can also be accelerated exactly (Mjolsness et al. 2009), and accelerated further by working hierarchically at multiple scales and/or using parallel computing (Orendorff and Mjolsness 2012). The same theory can be used to derive machine learning algorithms for the inference of reaction rate parameters from sufficient data (Wang et al. 2010) (cf. Golightly and Wilkinson 2011), although sufficient data may be hard to obtain. One can also develop approximate sampling algorithms by operator splitting, justified, e.g., by the Baker–Campbell–Hausdorff theorem (BCH), and/or by moment closure methods such as those discussed in Sect. 4.

Symbolic and numerical solutions of some simple stochastic models are discussed briefly in the Supplementary Material, Section 7.4.1.

Thus, unordered collections of pure chemical reactions provide a simple example of a modeling language with a compositional semantics. But for most biological modeling, we need much more expressive power than this.

### Parameterized Reaction Rules

The first modeling language escalation beyond pure chemical reaction notation is to particle-like objects or “agents” that bear numerical and/or discrete parameters which affect their reaction reaction rates. For example, the size of a cell may affect its chances of undergoing cell division. This kind of multiset rewrite rule can be generalized to (Mjolsness 2010)

6 $$\begin{aligned} {\left\{ \tau _{\alpha ( p) }[ x_{p}] |p \in L_{r}\right\} }_{*} \longrightarrow {\left\{ \tau _{\beta ( q) }[ y_{q}] | q \in R_{r}\right\} }_{*} \quad \mathbf{with } \quad \rho _{r}\left( \left[ x_{p}\right] ,\left[ y_{q}\right] \right) . \end{aligned}$$

Here, we have switched from molecule-like term names $A_\alpha$ to more generic logic-like term names $\tau _{\alpha ( p)}$. The parameters $x_{p}, y_{q}$ of each term (indexed by positions *p*, *q* in their respective argument lists, and which may themselves be vectors ${{{\varvec{x}}}}_{p}$) introduce a new aspect of the language, analogous to the difference between predicate calculus and first-order logic: each parameter can appear as a constant or as a variable, and the same variable $X_c$ can be repeated in several components of several parameter lists in a single rule. Thus, it is impossible in general to say whether two parameters in a rule are equal or not, and thus whether two terms $\tau _{\alpha ( p) }[ x_{p}]$ in a rule are the same or not, just from looking at the rule—that fact may depend on the values of the variables, known only at simulation time. The *p*, *q* subindex notation is as in Mjolsness (2013). The reaction rate $\rho _{r}( [ x_{p}] ,[ y_{q}] )$ now depends on parameters on one or both sides of the rewrite rule which can be factored [automatically in a declarative environment (Yosiphon 2009)] into a rate depending on the LHS parameters only and a conditional distribution of RHS given LHS parameters.

#### Examples: Cell Division and Dynamic Cytoskeleton

Both multicellular tissue and intracellular cytoskeleton topologies change, discontinuously of course, in ways that could be modeled with parameterized reaction rules in a flexible declarative language. For example, a stem cell of volume *V* might divide asymmetrically yielding a stem cell and a transit-amplifying cell in for example mouse olfactory epithelium (Yosiphon 2009):

7 $$\begin{aligned} \begin{aligned}&{\text {stemcell}}[ {{{\varvec{x}}}},V,\ldots ] \longrightarrow {\text {TAcell}}[ {{{\varvec{x}}}}+{\Delta } {{\varvec{x}}},V/2,\ldots ] ,{\text {stemcell}}[ {{{\varvec{x}}}}-{\Delta } {{{\varvec{x}}}},V/2,\ldots ] \\&\quad \mathbf{with }\ \ {\hat{\rho }}( V) {\mathcal {N}}( {\Delta } {{{\varvec{x}}}};c V^{1/d}) , \end{aligned} \end{aligned}$$

where ${\hat{\rho }}( V)$ is a probability per unit time or “propensity” for cell division depending on cell volume, and ${\mathcal {N}}( {\Delta } {\mathbf{x }};c V^{1/d})$ is a Gaussian or normal probability density function with diagonal covariance proportional to a lengthscale set by cell volume. It is up to the modeler to impose appropriate invariances in such a model. In this case, Galilean invariance is ensured by fact that the propensity function depends on position only through ${\Delta } {{{\varvec{x}}}}$, a difference of cell position vectors. Rotational symmetry could be broken by the prominent apical-basal axis in such a $d=2$ model of a pseudostratified epithelium.

Another stem cell application was to models of plant root growth and pattern formation regulated by the auxin growth hormone (Yosiphon 2009; Mironova et al. 2012; Mjolsness 2013), implemented in a computer algebra system (Yosiphon 2009; Shapiro et al. 2013); cf. Julien et. al (2019) in this issue for auxin-based plant shoot patterning. These examples show that with parameters, reaction-like rewrite rules can represent (for example) both cellular and molecular processes in the same model, and of course their interactions, which is a key expressiveness capability for multiscale modeling. Further discussion of this and other cell division examples is presented in Supplementary Material Section 7.3.1.

The grammar of Equation (35) happens to have just one object on the LHS of each rule, so it is “context free” and amenable to analysis. Generally, that is not the case even for cell division grammars like Equation (34); even less so for biological many-to-one transitions such as the fusion of mitochondria or of muscle cell, or the merging of microtubule fibers in cytoskeleton.

**Fig. 2:**
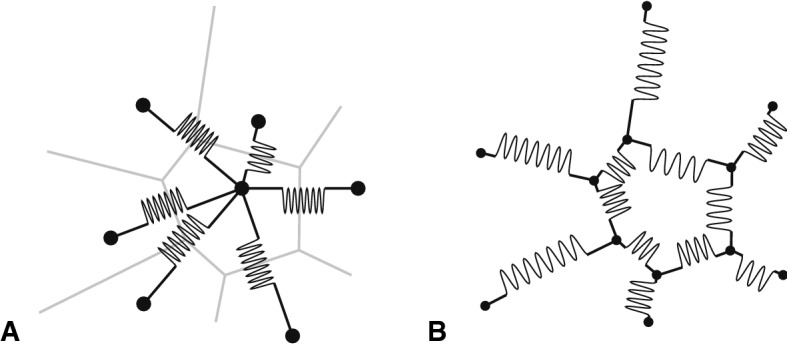
**a** Cell–cell spring biomechanical model. Potential energy *V* for each spring can be something like, e.g., a Lennard–Jones potential that is repulsive at short range, attractive at intermediate range, and flat (implies forceless) at long range; another such form is a breakable spring with $V(\mathbf{x }_p , \mathbf{x }_q)= (1/2)\sum _{q \in \text {Nbrs}(p)} k_{pq} c_{pq} [(|\mathbf{x }_p - \mathbf{x }_q| - l_{pq}|^2 - {{\Delta } l_{pq}}^2]$, where $c_{pq} \in \{0,1\}$ is chosen to minimize *V* at large separations ($c_{pq} = 0$ corresponds to a broken spring with continuity of potential energy at the breaking point). Parameters are spring constant (strength) $k_{pq}$, resting length $l_{pq}$, and breaking stretch distance ${{\Delta } l_{pq}}$. **b** Cell wall spring biomechanical model. Springs again have nonzero resting lengths, but cannot break. Cf. more detailed polygonal tessellation models in Wolff et al. (2019). Reproduced from Shapiro et al. (2013); courtesy Bruce Shapiro

A related but more detailed approach to 2D and 3D cell division modeling was taken in the shoot apical meristem (SAM) dynamical patterning model of Jönsson et al. (2006), in which cell positions and radii were again (as in 1D) the dynamical variables, determined by the mechanics of breakable springs in viscous media, and cell–cell interface areas were determined by the chordal intersections of corresponding cellular regions (Fig. 2a). The “Organism” C++ code in which this model was implemented (Jönsson et al. 2018) is not fully declarative, but is flexible enough to serve as the back end for a declarative model by translation of input files and to output similar files.^1^ It supports bidirectional coupling of regulatory networks such as regulated active auxin transport and/or gene regulation network models of SAM morphogenetic patterning [e.g., Jönsson et al. (2005); see also Banwarth-Kuhn et al. (2018) in this issue] to the biomechanics. A similar mechanical/regulatory model with breakable springs for the stem cell niche of mouse olfactory receptor neurons was implemented in the Plenum prototype implementation of the dynamical grammars declarative modeling language (Yosiphon 2009). A similar cell-centered model was used recently to model neural crest cell group migration, with stronger springs at the rear of each cell group representing multicellular cytoskeletal structures there (Shellard et al. 2018).

**Fig. 3:**
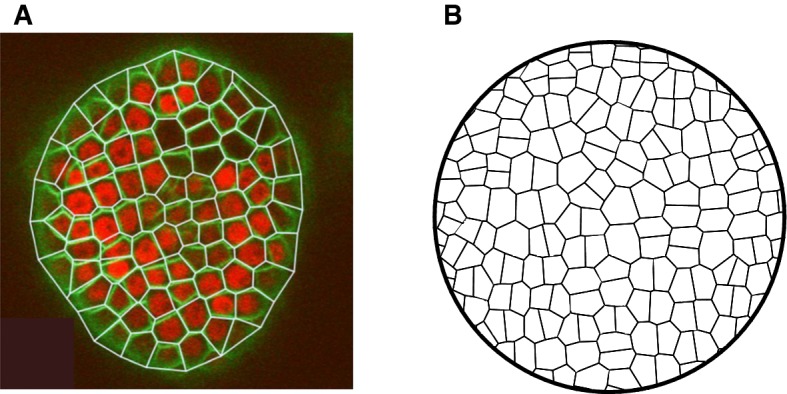
**a** Trimmed Voronoi diagram closely matches cell walls in *Arabidopsis thaliana* shoot apical meristem outer L1 cell layer 2D geometry derived from confocal laser scanning microscopy. Green: cell wall marker. Yellow: Voronoi diagram edges. Red: nuclear marker. Reproduced from Shapiro et al. (2012); courtesy Bruce Shapiro and Marcus Heisler. **b** Growing SAM geometry produced by Cellzilla declarative model, using optimized cell division rule as described in the text. Reproduced from Shapiro et al. (2013) (Color figure online)

As described in Supplementary Material Section 7.3.2, more detailed quantitative cell division rules can create growing convex polygonal patterns that appear qualitatively similar to those of derived from microscope imagery of SAM tissue as shown in Fig. 3.

*Dynamic cytoskeleton* All of these examples of coarse-scale models of plant cell division could and probably should be elaborated at a much finer biophysical scale. But doing so requires the introduction of models of cytoskeleton in general and microtubule dynamics in particular, a problem of current research interest (Vemu et al. 2018; Chakrabortty et al. 2018). That is because the role microtubules play in determining the plane of the cortical pre-prophase band, which in turn correlates well with the subsequent septation and choice of division plane. Here, we simply observe the composable rule-like behavior of some of the principal processes that cortical microtubules undergo: (1) nucleation of new MTs, often in association with old ones; (2) treadmilling, in which tubulin subunits are added to the ragged growing “+ end” of a microtubule and removed from the “− end”; (3) probabilistic transitions of $+$ end state among growth, pause, and catastrophic depolymerization (Shaw et al. 2003); (4) collision of one CMT into the side of another in the 2-dimensional cell cortex, resulting depending on collision angle in (4a) “zippering” or “bundling” into a CMT bundle if the collision angle is small, or else (4b) an apparently stochastic choice between (4b1) colliding $+$ end goes into catastrophe state, or (3b2) colliding $+$ end crosses over and continues past the other CMT, forming a stable junction with it; (5) katanin-induced severing of a CMT far from either end; and (6) biomechanics of bending. Other processes may have to do with anchoring the CMT to the cell membrane and cell wall. Processes 4a, 4b1 and 4b2 are illustrated in Supplementary Material Section 7.3.3, Figures 7 and 8. One way to express some of these processes [(1), (2), part of (3), and (4a)] in a graph grammar will be discussed in Sect. 3.2.

Even for multilevel modeling of a single-scale reaction network, dynamic parameters allow for the possibility of aggregate objects that keep track of many individual particles including their number. Such aggregate objects would have their own rules as discussed in Yosiphon (2009, Section 6.4), possibly obtained from fine-scale reaction rules by meta-rule transformation.

A detailed description of the semantics of *parameterized* rewrite rules as developed in Mjolsness (2010) is presented in Supplementary Material Section 7.2.2. It is followed in Section 7.2.3 by a discussion of the high degree of intrinsic parallelism of the resulting semantics.

Equations (6) and (28) comprise the syntax and semantics of the basic ruleset portion of stochastic parameterized grammars (SPG) language of Mjolsness and Yosiphon (2006) and Yosiphon (2009). A simulation algorithm is derived in Mjolsness (2013). *Equivalence* of models can again be defined as “particle equivalence,” Equation (25). Other features such as submodels, object type polymorphism, and graph grammars were also included. The idea of a biological modeling language whose models take the form of grammars goes back to L-systems (discussed below); continuous-time versions of biomodel grammars to which it would be easy to add differential equation rules goes back at least to Mjolsness et al. (1991) and Prusinkiewicz et al. (1993); in the former case there is also in principle an optimization-based semantics for choosing which collection of discrete-time rules to fire.

Parameterized reaction rule notation is fundamentally more powerful than pure chemical reactions, because now reaction/process rates $\rho _{r}( [ x_{p}] ,[ y_{q}] )$ can be functions of all the parameters involved in a rule, *and* a rule firing event can change those parameters. It becomes possible to express sorcerer’s apprentice models which purport to accomplish an infinite amount of computing in a finite simulated time, though this situation can also be avoided with extra constraints on the rate functions in the language.

Brief comparisons to other rule- or grammar-like modeling languages, namely L-systems and the BioNetGen modeling languages, are provided in Supplementary Material Section 7.2.4. Another relevant graph rewrite rule modeling language is Kappa (Danos et al. 2007) discussed in Sect. 3.2.

We have argued that summation of time-evolution operators corresponds to model compositionality in terms of processes. To a lesser extent, models are compositional in their *objects* as a result, since each object participates in a limited set of processes particularly if processes are disaggregated by spatial position as they will be in Sect. 2.3; thus locality expressed as commutation of spacelike separated operators helps to license a degree of decomposition by object as well. But process compositionality is primary.

Parameterized rewrite rule models would seem to be non-spatial, but particle movement through space can already be encoded using discrete or continuous parameters that denote spatial location. Such motion would however have to occur in discrete steps due to discrete-time rule firing. The solution to that limitation (among others) is another language escalation.

### Differential Equation Rules

Another form for parameterized rewrite rules licenses locally attached ordinary differential equations (ODEs) for continuous parameters, as in Mjolsness et al. (1991), Prusinkiewicz et al. (1993) and Mjolsness (2013), as part of the language *L*:

8 $$\begin{aligned} \begin{aligned}&{\left\{ \tau _{\alpha ( p) }[ x_{p}] |p \in L_{r}=R_{r} \right\} }_{*} \longrightarrow {\left\{ \tau _{\alpha ( p) }[ x_{p}+d x_{p}] | q \in R_{r}= L_{r} \right\} }_{*} \\&\quad {\;\mathbf with \;} \Bigg \{ {d x_{p}}=v_{p}( \left[ x_{k}\right] ){d t} \Bigg | p \Bigg \} \text { , i.e., } \\&{\left\{ \tau _{\alpha ( p) }[ x_{p}] |p \in L_{r}=R_{r} \right\} }_{*} \longrightarrow {\left\{ \tau _{\alpha ( p) }[ x_{p}] | q \in R_{r}= L_{r} \right\} }_{*} \\&\quad {\; \mathbf with \;} \Bigg \{ \frac{\mathrm{d} x_{p,j}}{\mathrm{d} t}=v_{p,j}( \left[ x_{k}\right] ) \Bigg | p,j \Bigg \} , \end{aligned} \end{aligned}$$

where the second form uses component rather than vector notation for all the ODEs. The first form is more readily generalizable to stochastic differential equations (SDEs) $$\{d x_{p}=v_{p}( [ x_{k}] ){d t} + w_{p}( [ x_{k}]) dB_t$$ where $$ dB_t$$ is a Brownian motion. The ODE semantics is given by the corresponding differential operators:

9 $$\begin{aligned} {{\hat{W}}}_{\mathrm {drift}}=-\int \mathrm{d}\left\{ x\right\} \int \mathrm{d}\left\{ y\right\} {\hat{a}}( \left[ y\right] ) a( \left[ x\right] ) \left( \sum \limits _{j}\nabla _{ y_{j}}v_{j}( \left[ y\right] ) \prod \limits _{k}\delta ( y_{k}-x_{k}) \right) \end{aligned}$$

as shown for example in Mjolsness (2013). The SDE case is discussed in Mjolsness and Yosiphon (2006, section 5.3).

Equations (6), (8) and (28), (9) comprise the syntax and semantics, respectively, of the basic ruleset portion of dynamical grammars (DG) language of Mjolsness and Yosiphon (2006) and Yosiphon (2009), by addition of differential equations to SPGs. A simulation algorithm is derived from the time-ordered product expansion in Mjolsness (2013). The generalization to operator algebra semantics for partial differential equations (PDEs) and stochastic partial differential equations (SPDEs), as a limit of spatially discretized ODE and SDE systems, is outlined in Mjolsness (2010). These differential equation bearing rules can be used to describe processes of growth and movement of individual particle-like objects, as in “agent-based” modeling. Further comments on differential equation rules are made in Supplementary Material 7.2.5. Further comments on the reduction relations between alternative semantic maps are made in Supplementary Material 7.2.6.

Complex biological objects often have *substructure* whose dynamics is not easily captured by a fixed list of parameters and a rate function or differential equation that depends on those parameters. Extended objects such as molecular complexes, cytoskeletal networks, membranes, and tissues comprising many cells linked by extra-cellular matrix are all cases in point. There can and sometimes should be several levels of substructure in a single biological model. We now wish to extend the syntax and semantics of the foregoing class of declarative languages to handle extended objects systematically, by creating a compositional language and semantics for biological *objects* as well as processes, and then extending the semantics for processes accordingly. In the case of discrete substructure, this can be done with labeled graph structures (discussed in Sect. 3.1). In some cases, the sub-objects (such as lipid molecules in a membrane, or long polymers in cytoskeleton) are so numerous that an approximate spatial continuum object model is justified and simpler than a large spatially discrete model. Spatial continuum models with geometric objects can be built out of manifolds and their embeddings, along with biophysical fields represented as functions defined on these geometries, in various ways we discuss in Sect. 3.

## Extended Objects

Can we declaratively model non-pointlike, extended biological objects such as polymer networks, membranes, or entire tissues in biological development? To achieve constructive generality in treating such extended objects we introduce, in Sect. 3.1, ideas based on discrete graphs and their possible continuum limits. These ideas include: graded graphs, abstract cell complexes, stratified graphs, and combinations of these ideas. Dynamics by rewrite rules, beginning with graph rewrite rules, are developed in Sect. 3.2. In Supplementary Material Section 7.4.2, we discuss the more general nonconstructive types of extended objects that we may wish to approximate constructively; here, we turn to constructive types of extended objects.

### Constructive Extended Objects via Graphs

Discrete graphs, especially when augmented with labels, are mathematical objects that can represent computable objects and expressions at a high level of abstraction. In Supplementary Material Section 7.2.7, we give standard definitions of graphs (directed and undirected) and graph homomorphisms, and thence (vertex-)labeled graphs, bipartite graphs, cliques, trees, directed acyclic graphs (DAGs), and their use in abstract syntax trees; also the standard functors from undirected to directed graphs and from edge-labeled to vertex-labeled graphs; also binary graph operators including graph sum, cross product, box product, strong product, and graph function.

A special case of a labeled graph is a *numbered graph* with integer labels $\lambda _i^{\prime } = i$, $|{\varLambda }^{\prime }| \geqslant |V|$, and the labeled graph homomorphism to ${K}^+_{{\varLambda }^\prime }$ is one-to-one (but not necessarily onto), so each vertex receives a unique number in ${\varLambda }^\prime = \{1 \ldots k \geqslant |V|\}$. Then, one way to express a labeled graph $G \rightarrow _\text {Graph} {K}^+_{{\varLambda }}$ is as the composition $G \rightarrow _\text {Graph} {K}^+_{{\varLambda }^\prime } \rightarrow _\text {Graph} {K}^+_{{\varLambda }}$ of a graph numbering $G \rightarrow _\text {Graph} {K}^+_{{\varLambda }^\prime }$ followed by a relabeling determined by a mapping of sets ${{\varLambda }^\prime } \rightarrow _\text {Set} {{\varLambda }} \mathbin {{\dot{\cup }}} \{\varnothing \}$, with $\varnothing$ the value taken on unused number labels *i*. The possibility of unused numbers ($|{\varLambda }^{\prime }| \geqslant |V|$) will be needed when consistently labeling two different graphs. We consider graph homomorphisms to five particular integer-labeled graphs defined carefully in Supplementary Material Section 7.2.8: ${{\mathbb {N}}}^+$, the nonnegative integers with successor links, for level number labels; ${J}^+_D$, the integers $\{0, \ldots D\}$ with direct and indirect predecessor links, for dimension labels; ${{{\mathbb {N}}}}_D^{+ \text {op}}$, the integers $\{0, \ldots D\}$ with direct predecessor links; $C_D \equiv {{\mathbb {N}}}^+ \Box {J}^+_{D}$; and ${{\tilde{C}}}_D \equiv {{\mathbb {N}}}^+ \Box {{{\mathbb {N}}}}_D^{+ \text {op}}$. Then, we can define the following:

A *graded graph* is a homomorphism from a graph *G* to ${{\mathbb {N}}}^+$. It labels vertices by a level number. Additional assumptions may allow an undirected graded graph to “approach” a continuum topology such as a manifold or other metric space, in the limit of large level number. The metric can be derived via a limit of a diffusion process on graphs. For example, *D*-dimensional rectangular meshes approach ${{\mathbb {R}}}^D$ this way (e.g., Mjolsness and Cunha 2012).

A *stratified graph* of dimension *D* is a homomorphism from a graph *G* to ${J}_D^+$. It labels vertices by a “dimension” of the stratum of the stratum they are in. The reason for reversing the edges in the directed graph case, compared to the graded graph definition, is that the level-changing edges then correspond to standard boundary relationships from higher to lower dimensional strata. In the undirected graph case, such a homomorphism is equivalent to a graph whose vertices are labeled by dimension number, since it imposes no constraints on edges.

*Abstract cell complex* a special case of a stratified graph of dimension *D* is a graph homomorphism from *G* to ${{\mathbb {N}}}_D^{+ \text {op}}$, the directed graph of integers $\{0, \ldots D\}$ with self-connections and immediate predecessor connections $i\rightarrow i-1$ within the set. Since ${{\mathbb {N}}}_D^{+ \text {op}}$ maps to ${J}^+_{D}$ by graph homomorphism, this is (equivalent to) a special case of stratified graphs.

A *graded stratified graph* of dimension *D* is a graph homomorphism from *G* to $C_D = {{\mathbb {N}}}^+ \Box {J}^+_{D}$. Two nodes in $C_D$ are connected, permitting corresponding edge connections in *G*, iff either their level numbers are equal (and, in the directed case, the source node dimension is $\geqslant$ the target node dimension), or if their level numbers differ by one and their dimensions are equal. Because *G* projects to both ${{\mathbb {N}}}^+$ and ${J}^+_{D}$, a graded stratified graph maps straightforwardly (by functors) to both a graded graph and to a stratified graph. However, it enforces an additional consistency constraint on level number and dimension: they cannot both change along one edge (and, in the directed case, the source node dimension must be $\geqslant$ the target node dimension).

A *graded abstract cell complex* of dimension *D* is likewise a graph homomorphism from *G* to ${\tilde{C}}_D = {{\mathbb {N}}}^+ \Box {{\mathbb {N}}}_D^{+ \text {op}}$.

Our aim with these definitions is to discretely and computably model continuum stratified spaces (including cell complexes) in the limit of sufficiently large level numbers. Stratified spaces are topological spaces decomposable into manifolds in more general ways than CW cell complexes are. In addition, we would like each manifold to be a differentiable manifold with a metric related to a Laplacian, since that will be the case in modeling spatially extended biological or physical objects.

Thus, we arrive at the constructive *slice categories*$S(H): G \rightarrow H$ with morphisms $h: G \rightarrow H$ that makes a commutative diagram $\varphi _G = h \circ \varphi _{G^{\prime }}$: 
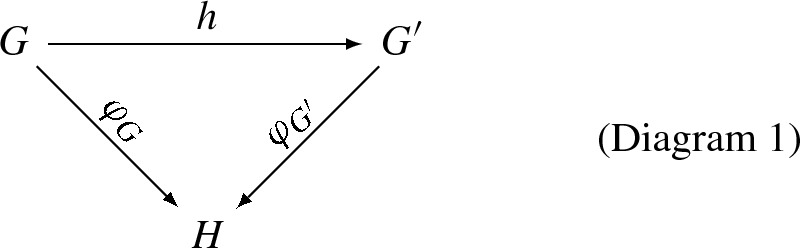
where $H = {{\mathbb {N}}}^+, \;\; {J}_D^+, \;\; {{\mathbb {N}}}_D^{+ \text {op}}, \;\; C_D, \;\; \mathrm{or } \;\; {\tilde{C}}_D$. Here, it is essential that each possible target graph *H* includes all self-connections. In particular, a homomorphism of graded graphs is a graph homomorphism with $H={{\mathbb {N}}}^+$, so graded graphs together with level-preserving graph homomorphisms form a slice category.

For a discrete approximation to a stratified space by a stratified graph, we can identify the “graph strata,” and their interconnections by boundary relationships, as connected components of constant dimension. So, by eliminating links between nodes of different dimension, and then finding the connected components that remain, we identify the *strata* in a stratified graph (or in a stratified labeled graph). In this commutative diagram: 
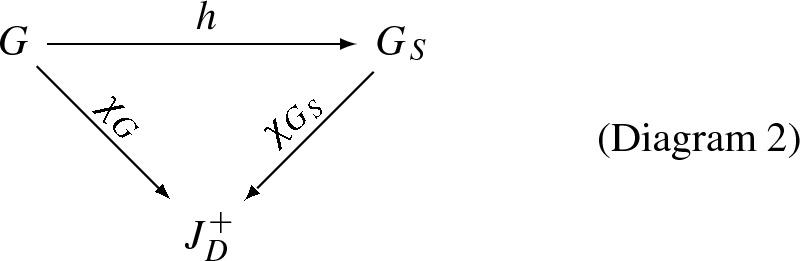
each inverse image $$(\chi _{G_S}^{-1})(d)$$ must be a fully disconnected graph, with each vertex then corresponding (by $$h^{-1})$$ to a *d*-dimensional stratum (maximal connected *d*-dimensional component) in *G*. The directed graph associated to $$G_S$$ (directed by dimension number in the case of undirected graphs) is a DAG, due to disconnection within each dimension. The graph homomorphism *h* becomes a homomorphism of stratified graphs.

#### Observation 1

The resulting $G_S$ in Diagram 2 is the graph of strata, a minimal structure for modeling complex geometries. *It is therefore a natural graph on which to specify rewriting rules for major structure-changing processes* such as biological cell division, mitochondrial fission/fusion, neurite or cytoskeletal branching, and other essential processes of biological development.

The geometry of cytoskeleton, in particular, is better captured by stratified graphs and stratified spaces than by cell complexes, because 1D and 0D cytoskeleton is often embedded directly into 3D cytosol rather than into 2D membrane structures (this violates the CW complex assumption that cells are *d*-dimensional balls, whose boundaries map continuously into finite unions of lower dimensional balls, both because dimension 2 is skipped between 3 and 1, and because 3D cytosol is not generally homeomorphic to a ball if it can be punctured by a $1\hbox {D}+0\hbox {D}$ cytoskeleton with 1D loops and/or multiple anchor points in the biological cell’s surface).

In many developmental biology systems, the spatial dynamics involves nontrivial changes in geometry and/or topology of extended biological objects. By using rewrite rules for the graph of strata, together ODE-bearing rules for the parametric embeddings of individual strata into 3D space, we now have in principle a way to represent such dynamics mathematically and computationally.

#### Observation 2

In a graded stratified graph (and therefore also in a graded abstract cell complex), a useful special case occurs if $G_S$ restricted by level number stabilizes beyond a constant number of levels, so the description in terms of strata has a continuum limit $G^*_S$. In that case, *the limiting*$G_S$*is also the natural locus for verification of compatible boundary conditions between PDEs* of different dimensionality governing the evolution of biophysical fields defined on continuum-limit strata. Here, “compatibility” includes local conservation laws.

The edges remaining in $G_S$, connecting strata of unequal dimensionality, model in-contact relations such as “boundary” and/or “inside”. Further constraints are needed to disentangle these alternatives. Similar ideas to $G^*_S$ may be involved in the “persistent homology” approach to unsupervised learning of data structure (Bendich et al. 2007). A further discussion of the relation between stratified graphs abstract cell complexes as defined here, and abstract cell complexes as defined elsewhere is given in Supplementary Material Section 7.2.8.

Given an extended object *G* constructed as outlined here, it is generally also necessary to define some dynamics that run “in” or “on” such an object: diffusing or otherwise moving particles with position $x \in G$ described by a spatial probability distribution *p*(*x*, *t*), or other dynamical fields *f*(*x*) at a given moment of time *t*. Several approaches to this issue are outlined in Supplementary Material Section 7.4.3.

#### Relation to PDEs

If some of the strata in $G_S$ are host to partial differential equations (respecting possibly dynamical boundary conditions at adjacent lower and higher dimensional strata) then $G_S$ will be too minimal for solution algorithms like finite elements or finite volumes, and those strata may have to be meshed. In that case, the strata of $G_S$ may need to be subdivided sufficiently finely into patches that can each host a local coordinate system, compatible with its neighboring patches of different dimension and (under one possible strategy) separated by extra artificial boundary strata patches from its neighbors of the same dimension. More detailed approaches to this issue are outlined in Supplementary Material Section 7.4.3.

By these various means, one would like to generalize from ODE-bearing rules to PDE-bearing rules which would be of two basic types: (1) PDEs for the evolution of biophysical fields such as diffusion within a manifold (possibly including a hyperbolic term for finite speed *causal* information propagation, as in the hyperbolic Telegrapher’s Equation derivable for stochastic diffusion (Kac 1974) which has as a limit the parabolic heat equation), and (2) PDEs for the evolution of the embeddings of strata into higher strata, such as cytoskeleton mechanics (1D into 2D or 3D) or the biomechanics of 2D membranes embedded into ordinary three dimensional Euclidean space. This could be accomplished by way of level sets and local stress fields, for example.

Such dynamic biophysical fields can also influence the (discontinuous and usually much slower) topology-changing dynamics by which the number and connectivity of a model’s geometric strata change—such as in plant or animal cell division. We study such dynamic graph structure next.

### Dynamics of Graphs

There are at least two ways to mathematically define the semantics of graph rewrite rules for use in biological modeling of extended objects. The operator algebra approach pursued in this paper, extending the form of rewrite rule semantics given in Sect. 2, has been used as the theory behind some molecular complex modeling and tissue-level developmental modeling methods (Johnson et al. 2015; Mjolsness 2013). It takes advantage of the algebra of operators to blend with continuous-time process models by scalar multiplication of rates and operator addition of parallel processes, thereby also gaining compatibility with quantitative methods of statistical mechanics and field theory in physics, and with machine learning by continuous optimization. The essential step is to express natural graph-changing operations, including a collection of graph rewrite rules, in terms of an operator algebra generated by the operators for the individual rules. A second approach, the graph homomorphism pushout diagram approach championed in Ehrig et al. (2006), has been used in molecular complex modeling (Danos et al. 2012) by providing a mathematical semantics for the “Kappa” modeling language (Danos et al. 2007). It takes advantage of the category theory formulation of graphs, discussed above. We will first develop operator algebra semantics for graph grammars in continuous time in detail. Then, we will briefly compare it with the pushout semantics approach which leverages category theory in a way similar to our discussion above.

#### Graph Rewrite Rule Operators

By generating unique (e.g., integer-valued) “ObjectID” parameters for each new object created in a parameterized grammar rule, it is possible to implement graph grammar rules by parameterized grammar rules (Sect. 2.2) alone, just using repeated ObjectID values to represent graph links. Since parameterized grammar rules are mapped by semantic map ${\varPsi }(M)$ to an operator algebra, the composition of two maps ${{{\mathcal {I}}}} \circ {\varPsi }$ defines an operator algebra semantics for graph grammars. This route was detailed in Mjolsness (2005) and Mjolsness and Yosiphon (2006), and implemented declaratively in Yosiphon (2009). But it is also possible (Mjolsness 2010) to define graph grammar rewrite rule semantics directly as a continuous-time dynamical system, using operator algebra as in the previous semantics definitions of Sect. 2.

Suppose we have two labeled graphs $G _1: G^{\text {pure}}_1 \rightarrow _\text {Graph} K^+_{{\varLambda }_1}$ and $G_2: G^{\text {pure}}_2 \rightarrow _\text {Graph} K^+_{{\varLambda }_2}$. We decompose them each into a numbered graph $G^{\text {num}}_i : G^{\text {pure}}_i \rightarrow _\text {Graph} K^+_{\{1,\ldots k_i \geqslant |V_i|\}}$ and a relabeling $K^+_{\{1,\ldots k_i \geqslant |V_i|\}} \rightarrow _\text {Graph} K^+_{{\varLambda }_i}$ determined by a mapping of sets $\{1,\ldots k_i \} \rightarrow _\text {Set} {{\varLambda }_i}$, determined in turn by an ordered listing of labels ${\varvec{\lambda }}_i$, possibly augmented by the nullset symbol $\varnothing$. The whole decomposition can be denoted . We are interested in graph rewrite rules  that respect a single consistent numbering of vertices of the two numbered graphs before their relabelings. In that case, vertices in $G_1$ and $G_2$ that share a vertex number are regarded as “the same” vertex *v*, before and after rewriting (similar to the shared graph “*K*” in the double pushout approach discussed in Supplementary Material Section 7.2.10), so that any graph edges contacting *v* and not mentioned in the rewrite rule are preserved.

*3.2.1.1 Graph Rewrite Rule Examples* Here (Eq. 10) is an example pertaining to refinement of triangular meshes in 2D. This is one of four rewrite rules that suffice to implement a standard triangular mesh refinement scheme [similar examples were studied in Maignan et al. (2015)]. Three of those rules including this one deal with partially refined triangle edges, an intermediate state produced by the previous refinement of nearby triangles. It can also be interpreted as an (unlabeled) graded graph rewrite rule since it preserves the graded graph constraints on level numbers *l*, if they are satisfied initially (the other rewrite rules are similar but deal with the cases of zero, two, or three partially refined triangle sides). The labeled graph rewrite rule is:10Note that in this example, there is a shared numbering of nodes of the two graphs, and node numbers 1–4 occur in both graphs. This is equivalent to identifying the shared subgraph *K* in the double pushout semantics of Diagram 4 in Supplementary Material Section 7.2.10. Any extra edges that contact nodes 1–4 in a subgraph of the pool graph, e.g., parts of other nearby triangles, will remain after this rule fires. The other required rules are discussed in Supplementary Material Section 7.3.4, along with several variant grammars that would refine an initial triangular mesh in different ways.

In addition to the forgoing geometric example, we consider briefly how one might express some of the dynamics of visible plant cortical microtubule bundles previously described, in particular growth at a growing end (whether $+$ or − ends of individual MTs in the bundle); retraction at a retracting end, and bundling following front-to-side collision, in terms of graph grammar rules. Let a microtubule be an extended object represented as a chain of super-particles (each much larger than a tubulin dimer, representing a roughly straight cylindrical segment of one MT of length approximately on a lengthscale *L* that is several times an MT diameter, *or* of a parallel and/or antiparallel bundle of a few such cylindrical segments). Continuous parameters of such a fiber segment super-particle will include its center-of-mass position, and a unit vector pointing toward the growing end and away from the retracting end of an end segment (interior segments will have lengthwise unit vectors too, but their sign shouldn’t matter). Discrete parameters will include a four-valued categorical label $s \in \{{\text {internal}}, {\text {grow}}\_{{\text {end}}}, {\text {retract}}\_{{\text {end}}}, {\text {junct}} \}$ (or  in diagrams) for status as interior segment, growth-capable end segment, retraction-capable end, or junction segment, respectively.

A diagrammatic presentation of an MT graph grammar, with subscripts for the rule-local arbitrary but consistent numbering of vertices in left- and right-hand-side graphs of each rule, is here:11A corresponding textual presentation of this MT dynamic graph grammar (DGG) is given in Supplementary Material Section 7.3.3.

*3.2.1.2 Graph Rewrite Rule TheoryIn general* now, suppose *G* and $G^{\prime }$ are numbered graphs sharing a common numbering of their vertices, with index-ordered adjacency matrices $[g_{p q} | p, q]$ and $[g^{\prime }_{p^{\prime } q^{\prime }} | p^{\prime }, q^{\prime }]$ whose elements take values in $\{0,1\}$. Then, an unambiguous graph rewrite rule can be expressed as:12where the double angle brackets denote label substitutions: $\sigma (1) \mapsto \lambda _1, \sigma (2) \mapsto \lambda _2, \dots$ and $\sigma ^{\prime }(1) \mapsto \lambda ^{\prime }_1, \sigma ^{\prime }(2) \mapsto \lambda ^{\prime }_2, \dots$ where $\sigma : {{\mathbb {N}}} \rightarrow {{\mathbb {N}}}$ and $\sigma ^{\prime }: {{\mathbb {N}}} \rightarrow {{\mathbb {N}}}$ are strictly monotonically increasing mappings of initial segments of integers into the shared index space.

Given the foregoing graph rewrite rule syntax for graphs *G* and $G^{\prime }$ with adjacency matrices *g* and $g^{\prime }$, we now define an operator algebra semantics sufficient to bring all such graph rewrite rules into the general operator algebra/master equation formalism of previous sections. This is necessary to incorporate all the capabilities of the previously discussed languages by summing up time-evolution operators of the corresponding old and new kinds.

We give graph grammar rule operator semantics for the case of directed graphs in terms of binary state vectors for node and edge existence; in this case creation and annihilation operators all have dimension $2 \times 2$. As before, the notation is that indices may have primes or subscripts and are usually deployed as follows: *r* indexes rewrite rules, *i* and *j* index individual domain objects (now nodes or vertices in a graph), $\alpha$ and $\beta$ are generic indices or multiple indices, and *p* and *q* index elements in either side of a rule. Assuming there is at least one label that can be used to indicate node allocation from available memory, and provided the global state is initialized to have zero probability of active edges for unused nodes, then the off-diagonal portion of the operator algebra semantics is slightly modified from Mjolsness (2010):

13 $$\begin{aligned} \begin{aligned}&{{\hat{W}}}_{r} \; \propto \; \rho _{r}({{\varvec{\lambda }}} , {{\varvec{\lambda }}^{\prime }}) \sum \limits _{{\langle i_{1},\ldots i_{k}\rangle }_{\ne }} \Bigg [ \prod \limits _{p \in {\text {lhs}}(r) {\setminus } {\text {rhs}}(r)} \Big ( \prod _i E_{i_{p} \; i} E_{i \; i_{p}} \Big ) \Bigg ] \\&\quad \times \Bigg [ \prod \limits _{p^{\prime }, q^{\prime } \in {\text {rhs}}( r) } {\left( {{\hat{a}}}_{i_{p^{\prime }} i_{q^{\prime }}}\right) }^{{g^{\prime }}_{p^{\prime } \; q^{\prime } }} \Bigg ] \Bigg [ \prod \limits _{p^{\prime } \in {\text {rhs}}( r) } {{\hat{a}}}_{i_{p^{\prime }} {\lambda ^{\prime }}_{p^{\prime }}} \Bigg ] \\&\quad \times \Bigg [ \prod \limits _{p, q \in {\text {lhs}}( r) } {\left( a_{i_{p} i_{q}}\right) }^{g_{p \; q}} \Bigg ] \Bigg [ \prod \limits _{p \in {\text {lhs}}(r) } a_{i_{p} \lambda _{p}} \Bigg ] . \end{aligned} \end{aligned}$$

The sum over indices $\sum _{\langle i_{1},\ldots i_{k}\rangle _{\ne }}$ means that none of the indices are allowed to be equal to any of the others, in the sum. As in Equation (28) there could also be an integration over all the possible values of some rule variables, in this case a subset of the incoming and outgoing labels ${{\varvec{\lambda }}}$ and ${{\varvec{\lambda }}^{\prime }}$.

14 $$\begin{aligned} {{\hat{W}}}_{r} = \int \mathrm{d} X {{\hat{W}}}_{r}({{\varvec{\lambda }}}(X) , {{\varvec{\lambda }}^{\prime }}(X)) \end{aligned}$$

where ${{\hat{W}}}_{r}({{\varvec{\lambda }}}, {{\varvec{\lambda }}^{\prime }})$ is given by Eq. (13). However, we will not have occasion to use this extra flexibility. Additional technical discussion of the label encoding from which $\lambda$ is drawn appears in Supplementary Material Section 7.2.9.

The main four factors in lines 2 and 3 of Eq. (13) are as in Mjolsness (2010) and act in an analogous way to the previous rule semantics definitions: first (reading operator products right-to-left) all the vertex labels, hence all the vertices, of the incoming (LHS) graph are annihilated in an arbitrary order, “then” (instantaneously) all the edges of the incoming graph are annihilated in an arbitrary order, then all the vertices and vertex labels of the outgoing (RHS) graph are created, and then all the edges of the incoming graph are created. However, these ordered operations all happen with zero time delay in the model, and with the same binding of the indices $i_*$. We note that the RHS vertex indices $i_{p^{\prime }}$ get assigned uniquely since $({\hat{a}}_i)^2 = {\mathbf{0 }}$.

In addition to the main four factors, in line 1 provision is made for allocation and deallocation of integer-valued graph vertex indices *i* from a single central index list. The erasure operators $\Big ( \prod _i E_{i_{p} \; i} E_{i \; i_{p}} \Big )$ serve to maintain the invariance of the statements that (a) every vertex has either 0 or 1 labels present (1 defining an “active” vertex), and (b) edges that are present must connect two active vertices. Additional technical discussion of these points appears in Supplementary Material Section 7.2.9.

Equations (12) and (13), elaborated here from Mjolsness (2010) which omitted *E* factors, provide the built-in syntax and semantics for the basic ruleset portion of a proposed dynamical graph grammar (DGG) generalization of stochastic parameterized grammars (6) and (28) or dynamical grammars (6), (8) and (28),(9), all as outlined in Mjolsness 2005; Mjolsness 2010 and Mjolsness and Yosiphon (2006). The latter two references also map graph grammars to operator algebras by way of unique ObjectID node labels—similar to indices $i_p$ in that all that matters about them for the graph grammar is not their numerical values, but just whether two such numbers are equal or not.

Additional discussion of model equivalence under this semantics, together with comparison and contrast with related work on graph grammar rule semantics, appears in Supplementary Material Section 7.2.9.

#### Product of Graph Grammar Rules

We approach the multiplication of grammar rule operators in two steps. First, we consider the simpler form omitting cleanup post-factors:

15 $$\begin{aligned} \begin{aligned}&{{\hat{W}}}_{r} \; \propto \; \rho _{r}({{\varvec{\lambda }}} , {{\varvec{\lambda }}^{\prime }}) \sum \limits _{\langle i_{1},\ldots i_{k}\rangle _{\ne } } \Bigg [ \prod \limits _{p^{\prime }, q^{\prime } \in {\text {rhs}}( r) } {\left( {{\hat{a}}}_{i_{p^{\prime }} i_{q^{\prime }}}\right) }^{{g^{\prime }}_{p^{\prime } \; q^{\prime } }} \Bigg ] \Bigg [ \prod \limits _{p^{\prime } \in {\text {rhs}}( r) } ({{\hat{a}}}_{i_{p^{\prime }} {\lambda ^{\prime }}_{p^{\prime }}})^{h^{\prime }_{p^{\prime }}} \Bigg ] \\&\quad \times \Bigg [ \prod \limits _{p, q \in {\text {lhs}}( r) } {\left( a_{i_{p} i_{q}}\right) }^{g_{p \; q}} \Bigg ] \Bigg [ \prod \limits _{p \in {\text {lhs}}(r) } (a_{i_{p} \lambda _{p}})^{h_{p}} \Bigg ] . \end{aligned} \end{aligned}$$

where $h_{i_p} \in \{0,1\}$ is an indicator function for inclusion of vertex $i_p$ independent of its edges. Again, the sum over indices $\sum _{\langle i_{1},\ldots i_{k}\rangle _{\ne }}$ means that none of the indices are allowed to be equal to any of the others, in the sum. This form can leave and/or delete undeleted hanging edges, owing to the lack of erasure post-factor. If all $h_{i_p}=1$ this is the form used in Mjolsness (2010). The advantages of this form are that it is (a) subpermutation-invariant with respect to indexing, like Eq. (13), and (b) already in normal form (monomial in ${\hat{a}}_*$ times monomial in $a_*$), and therefore, the product of two such expressions takes the same general form, by repeatedly using the basic commutation relations of Sect. 2.1 or Supplementary Material Section 7.2.1, Equation (23d):

##### Proposition 1

The product of two operators taking the form of Eq. (15) can be rewritten as an signed-integer-weighted sum of expressions taking the same form. The product and the sum are equal, and graph-equivalent, and each is subpermutation-invariant with respect to indexing.

The proof is given in Supplementary Material Section 7.4.4.

Alternatively as in the graph rule semantics of Eq. (13), we may wish to eliminate hanging edges as part of the mechanics of the grammar operator algebra. So we consider the more general form:

16 $$\begin{aligned} \begin{aligned}&{{\hat{W}}}_{r} \; \propto \; \rho _{r}({{\varvec{\lambda }}} , {{\varvec{\lambda }}^{\prime }}) \sum \limits _{\langle i_{1},\ldots i_{k}\rangle _{\ne }} \Bigg [ \Big ( \prod \limits _{p \in B_r} \; \prod _{ i \ne i_q | \forall q \in {\bar{B}}_{r p}} E_{i_{p} \; i} \Big ) \Big ( \prod \limits _{p \in C_r} \; \prod _{ i \ne i_q | \forall q \in {\bar{C}}_{r p} } E_{i \; i_{p}} \Big ) \Bigg ] \\&\quad \times \Bigg [ \prod \limits _{p^{\prime }, q^{\prime } \in {\text {rhs}}( r) } {\left( {{\hat{a}}}_{i_{p^{\prime }} i_{q^{\prime }}}\right) }^{{g^{\prime }}_{p^{\prime } \; q^{\prime } }} \Bigg ] \Bigg [ \prod \limits _{p^{\prime } \in {\text {rhs}}( r) } ({{\hat{a}}}_{i_{p^{\prime }} {\lambda ^{\prime }}_{p^{\prime }}})^{h_{p^{\prime }}} \Bigg ] \\&\quad \times \Bigg [ \prod \limits _{p, q \in {\text {lhs}}( r) } {\left( a_{i_{p} i_{q}}\right) }^{g_{p \; q}} \Bigg ] \Bigg [ \prod \limits _{p \in {\text {lhs}}(r) } (a_{i_{p} \lambda _{p}})^{h_{p}} \Bigg ] . \end{aligned} \end{aligned}$$

where sets $B_r$, $C_r$, ${\bar{B}}_r$, and ${\bar{C}}_r$ are finite (assuming $\text {lhs}(r)$ and $\text {rhs}(r)$ are both finite); this form encompasses Eq. (13) in which case $B_r = C_r = \text {lhs}(r) {\setminus } \text {rhs}(r)$ and ${\bar{B}}_{r p}= {\bar{C}}_{r p} = \varnothing$ for all *p*. Owing to the sum over indices $\{i_1, \ldots i_k\}$ is again subpermutation-invariant.

##### Proposition 2

The product of two operators taking the form of Eq. (16) can be rewritten as an signed-integer-weighted sum of expressions taking the same form. The product and the sum are equal, and graph-equivalent, and each is subpermutation-invariant with respect to indexing.

Note: Less formally, the product of two graph rewrite rule operators is an integer-weighted sum of other graph rewrite rule operators in a slightly expanded operator algebra. This proposition establishes *an operator algebra of natural graph-rewriting operations*, including all operations in any given collection of graph rewrite rules, as the operator algebra generated by the probability inflow operators ${{\hat{W}}_r}$ for the individual rules. The probability outflow operators $D_r$ are also encompassed, by a variant of Equation (24) shown in Corollary 2.

The proof is given in Supplementary Material Section 7.4.4. As in Proposition 1, it uses commutation relations to convert expressions to normal form.

##### Corollary 1

The commutator $[{\hat{W}}_{r_1}, {\hat{W}}_{r_2}]$ of two operators under the semantics of Proposition  2 [taking the form of Eq. (16)] can also be rewritten as an integer-weighted sum of expressions taking the same form. The product and the sum are equal, and graph-equivalent, and each is subpermutation-invariant with respect to indexing. Likewise, the commutator $[{\hat{W}}_{r_1}, {\hat{W}}_{r_2}]$ of two operators under the semantics of Proposition  1 [taking the form of Eq. (13)] can also be rewritten as an integer-weighted sum of expressions taking the same form (13). Compared to the product ${\hat{W}}_{r_1} {\hat{W}}_{r_2}$, however, many summands may cancel in a commutator.

##### Corollary 2

The product and the commutator of two full graph rewrite rule operators $W_{r_1}, W_{r_2}$ (including their negative diagonal terms $-{\hat{D}}_{r_1}, -{\hat{D}}_{r_2}$) under the semantics of Proposition  2 [taking the form of Eq. (16)] can also be rewritten as an integer-weighted sum of expressions taking the same form. Likewise the product and the commutator of two full graph rewrite rule operators $W_{r_1}, W_{r_2}$ under the semantics of Proposition  1 [taking the form of Eq. (13)] can also be rewritten as an integer-weighted sum of expressions taking the same form. In either case, the product (or commutator) and the sum are equal, and graph-equivalent, and each is subpermutation-invariant with respect to indexing.

##### Proof

The diagonal terms $D_r$ are equal to ${\hat{W}}_{r^{\prime }}$ for a new rule $r^{\prime }$, not included in the model, in which the LHS of *r* is both the LHS and the RHS of $r^{\prime }$. The reason is that the LHS ${\setminus }$ RHS post-factor and any RHS ${\setminus }$ LHS prefactor of ${\hat{W}}_{r^{\prime }}$ are both empty, so Equation (32) for $D_r$ also equals ${\hat{W}}_{r^{\prime }}$ from Eq. (13). Thus, $D_r = {\hat{W}}_{LHS_r \rightarrow LHS_r}$, as in Equation (24) for particle semantics, but now for graphs. Proposition 2 then applies to ${\hat{W}}_{r}$ and $D_r$ alike, for all rules *r* in the model. $\square$

##### Observation 3

In this sense (Propositions 1 and 2 and Corollaries 1 and 2), there is a higher-level algebra, generated by any collection of graph rewrite rule operators, together with the identity operator $I =N^{(\varnothing )}= {\hat{W}}_{\varnothing \rightarrow \varnothing }$ that can be “implemented” by (mapped compositionally by operator algebra homomorphism to) a sufficiently large indexed collection of binary state variables with their own lower-level state-changing operator algebra.

##### Observation 4

*Semantics alternatives.* An alternative semantics to Eq. (13) could include factors of $\prod _\mathrm{LHS} (Z_{i_p i_q})^{{\bar{g}}_{p q}}$ where ${\bar{g}}$ is a 0/1-valued matrix representing a second type of graph edge that identifies *prohibited* connections on the LHS, and likewise $\prod _\mathrm{RHS} (Z_{i_p^{\prime } i_q^{\prime }})^{{\bar{g}}^{\prime }_{p^{\prime } q^{\prime } }}$ for the RHS. The normal form could put these new *Z* product factors just to the right of (acting just before) the corresponding factors for *g* and $g^{\prime }$ in Eq. (13). If corresponding entries of *g* and ${\bar{g}}$ both take the value 1, that inconsistency has no effect since their product has a factor $a Z = a (I-{\hat{a}}a) = a - (a {\hat{a}})a= a - (I-{\hat{a}}a)a = a - a + {\hat{a}}a^2 = 0$.

Instead of the creation and annihilation operators for Boolean edge variables, we could use creation and annihilation operators for ${{\mathbb {N}}}$-valued numbers of identical particles in definition (13) of ${\hat{W}}_r$. But (1) the handling of memory allocation and deallocation by *E* factors might have to be revised, and (2) graph grammar rules could have unintended semantics in terms of multigraphs: graphs with nonnegative integer edge weights. On the other hand, multigraphs and multigraph grammar rules can also be useful, if that is the intended semantics.

#### Slice Rewrite Rule Operators

The slice categories of Diagram 1, with $H = {{\mathbb {N}}}^+, \; {J}_D^+, \; {{\mathbb {N}}}_D^{+ \text {op}}, \; C_D, \; \mathrm{or } \; {\tilde{C}}_D$ for graded graph, stratified graph, abstract cell complex, graded stratified graph, and graded abstract cell complex, respectively, are all variants of the category of labeled graphs $\varphi : G \rightarrow H$ whose labels $\kappa$ are nodes of *H*, with extra constraints added on the integer-valued labels. We can encode these constraints in each case with a predicate $P_H({\varphi })$, and enforce them with a real-valued “gating” indicator function ${\varTheta }(P_H({\varphi }))$ which takes the value 1 if the predicate is satisfied and zero otherwise. If an ordering on the nodes of *G* is established, as we have assumed, then these objects become $P_H({{\varvec{\kappa }}})$ and an indicator function ${\varTheta }(P_H({{\varvec{\kappa }}}))$. In the foregoing graph rewrite rule semantics, such an ordering is established by the arbitrary indexing scheme of *p* and *q*. So we may generalize the graph rewrite rule to cover these slice categories as well by mapping $\rho _{\mathrm{slice} \; r}({{\varvec{\kappa }}} , {{\varvec{\kappa }}^{\prime }})$ to a corresponding $\rho _{\mathrm{graph} \; r}(({{\varvec{\kappa }}, {\varvec{\lambda }}}) , ({{\varvec{\kappa }}^{\prime }, {\varvec{\lambda }}^{\prime }}))$:

17 $$\begin{aligned} \rho _{\mathrm{graph} \; r}(({{\varvec{\kappa }}, {\varvec{\lambda }}}) , ({{\varvec{\kappa }}^{\prime }, {\varvec{\lambda }}^{\prime }})) = {\varTheta }(P_H({{\varvec{\kappa }}})) \times {\varTheta }(P_H({{\varvec{\kappa }}^{\prime }})) \times \rho _{\mathrm{slice} \; H,\; r}({{\varvec{\lambda }}} , {{\varvec{\lambda }}^{\prime }}) . \end{aligned}$$

The first indicator function in Eq. (17) could be omitted if the initial condition gives nonzero probability only to valid *H*-slice graphs and all rules in the grammar are valid *H*-rewrite rules, maintaining the validity conditions $P_H$ using the second indicator function in Eq. (17). The remaining operator products in Eq. (13) can remain the same, yielding the operator algebra semantics of these (and potentially other) slice category rewrite rules, complete with provision for extra domain-model specific labels $\lambda$.

In this way, we could *implement* special graph grammar syntax for slice graph rewrite rules, and thereby achieve summable operator algebra semantics for modeling languages with rewrite rules at the level of graded graphs, stratified graphs, abstract cell complexes, graded stratified graphs, and/or graded abstract cell complexes that could implement selected continuum limits such as mesh-approximable stratified spaces and CW complexes (as suggested in Diagram 3).

For example in the case of undirected graded graphs, we could let directed edges represent ${\Delta } l = +1$ edges and undirected edges represent ${\Delta } l = 0$. The triangle 2D mesh refinement example would then become:18Directed graded graphs could be represented too, with just one bit of edge label information to record whether there is a change of level number along a directed edge or not. In both cases, the integer-valued level number edge labels are removed, to be restored automatically by an implementation map ${{{\mathcal {I}}}}$ from slice graph grammar rule syntax to ordinary graph grammar rule syntax [this implementation map could even be expressed in the form of a declarative model transformation meta-grammar rule, mapping rules like (18) to a slight variant of rules like (10) with an AST for the labels]. Similarly, for stratified graphs one could label edges with ${\Delta } d$ and allow the interpretation process to restore *d*. For abstract cell complexes, one instead needs only to record one extra bit of edge information regarding *d*: ${\Delta } d \in \{0, -1\}$. As in the case of rule (10), with these extra edge labels rule (18) could be made more elaborate by retaining all relevant strata and their connections at smaller level numbers rather than just the graded graph “frontier” comprising the deepest substrata in each stratum.

##### Observation 5

In all the foregoing slice graph category cases, the implementation mapping on rewrite rules should match the implementation mapping on their semantics as proposed above, so that slice implementation at the model level commutes with semantics:
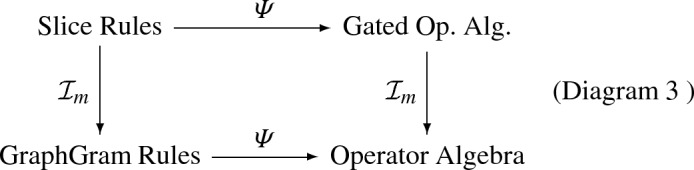
where, again, the slice rewrite rules can pertain to graded graphs, stratified graphs, abstract cell complexes, graded stratified graphs, or graded abstract cell complexes; the latter two could be used to support continuum limits such as mesh-approximable stratified spaces and CW complexes.

A systematic alternative to operator algebra semantics for graph rewrite rules is provided by the “double pushout” category-theoretic construction (Ehrig et al. 2006), using the category of graphs and graph transformations, discussed in Supplementary Material Section 7.2.10.

#### Efficient Implementations

We just saw that slice graph grammar rules can be implemented (efficiently) in terms of ordinary labeled graph grammar rules.

The efficient implementation of graph grammars rules themselves can also be considered. We have mentioned that they can be and have been implemented in terms of parameterized grammars with parameters devoted to recording integer-valued ObjectIDs. That implies that worst-case performance for parameterized grammars can be as bad as finding small unlabeled subgraphs in large unlabeled graphs, though finding subgraphs in practice is a lot easier than finding them in worst case, and labels help substantially. So one option is just to deploy algorithms that match small symbolic expressions, or use computer algebra systems that have done the same. But another option is available specifically for declarative modeling: to find the rules with the most commonly occurring rule firings in a model, and to use meta-grammars or a meta-language (discussed in Sect. 2) to transform those rules into submodels comprising rules taking only special forms that can be compiled into special-case efficient simulation code. Examples of special-form rules amenable to special-case simulation code include parameterless rules, terms with parameter sets that take only a few values, rules that consist only of differential equations, context-free grammar rules such as in Equation (35), 1D chain preserving rules such as Equation (34), and many other possibilities. Then, use strategies like operator splitting to simulate quickly most of the time, slowing down only for occasional higher-cost operations like cell division in a tissue model or bundling/zippering in a microtubule network model.

### Meta-Hierarchy Via Graphs

If we seek models in discrete mathematics for the idea of a “hierarchy” such as a hierarchy of biological systems and subsystems, or a hierarchy of modeling methods, the simplest possibility is a tree: a directed graph whose undirected counterpart has no cycles. This graph could be labeled with the names of the subsystems, methods, or other concepts in the hierarchy. Such a restrictive definition could be appropriate for a compositional hierarchy, or for a strict classification aimed at reconstructing clades, but not in general for a hierarchy of specializations or subsets in which a node may have several parent nodes. For that case, a less restrictive possibility is to model a hierarchy as a labeled DAG (directed acyclic graph), which has no cycles as a directed graph. Thus, a labeled DAG is a natural model for the idea of a hierarchy that is more general than a tree.

However, as the foregoing examples show, a hierarchy may be composed of items related in several different ways (composition, specialization, mutually exclusive specialization, and so on.) This fact suggests a further generalization. If the edge labels in a labeled DAG take values in a further DAG of possible relationships, themselves related only by specialization, the resulting compound structure can be called a *meta-hierarchy* since it encodes a hierarchy of interrelated hierarchies. This kind of structure has precedent in, for example, the more general “typed attributed graphs” of Ehrig et al. (2006).

In Sect. 5, we will consider a meta-hierarchy whose vertices index into (are labeled by) formal languages for modeling aspects of biology.

## Model Reduction

Model reduction can be a reasonable strategy to deal with biological complexity. Instead of picking out the most important variables and processes to include in a model a priori, one can include some reasonable representation of many variables and processes (although the model still would not be complete) with reasonable initial parameter values in a fine-scale model, and then computationally seek a smaller, coarser scale model that behaves in approximately the same way, on some set of “observables” or “quantities of interest,” in some relevant region of parameter space.

In addition to eliminating conditionally unnecessary state variables for simplicity’s sake, model reduction has the potential to: (a) enable scaling up to very large models through increased computational efficiency in simulation; (b) mathematically connect predictive models across scales of description for both causal authenticity and greater accuracy at each scale; (c) enable the study of the great diversity of possible emergent phenomena, as a function of the parameters, structures, and initial conditions of fine-scale models. Repeated model reduction can result in a *hierarchical stack of interrelated models*, with which to systematically maximize these advantages.

How can we use machine learning to perform the computational search for reduced models? Given enough data from a pure (parameterless) stochastic chemical reaction network, and the correct structure of the network, it is possible to learn the reaction rates (Wang et al. 2010; Golightly and Wilkinson 2011). In Sect. 4.1, we summarize how to learn not just reaction rates but an *effective reduced-state space model* in the form of a time-varying Boltzmann distribution in at least some examples by following very general principles, for the parameterless case (Johnson et al. 2015) and for the case in which parameters include spatial positions (Ernst et al. 2018).

The general theme of using machine learning to create computationally efficient reduced models is rapidly advancing. In computational chemistry for example Smith et al. (2017) develop a neural network for learning from, interpolating, and much more efficiently applying the energy and therefore force information computed in density functional theory fine-scale calculations. Likewise, other work (e.g., Burkardt et al. 2006) addresses difficult problems in fluid flow.

As for the general semantics (${\varPsi }$) and implementation (${{{\mathcal {I}}}}$) families of structure-respecting mappings, we will denote model reduction mappings by a mapping family symbol ${{\mathcal {R}}}$.

### Learning Boltzmann Distribution Dynamics

In the parameterless rewrite rule [e.g., a chemical reaction as in Eq. (2)] case, we learn a coarse-scale approximation ${\tilde{p}}$ of *p* as a time-varying version of a Boltzmann distribution or Markov random field (MRF):

19 $$\begin{aligned} {\tilde{p}}({{{\varvec{s}}}}, t; [ \mu _\alpha | \alpha ] ) = \frac{1}{\mathcal {Z}(\mu (t))} \exp \left[ - \sum _\alpha \mu _\alpha (t) V_\alpha (s_i \in C_\alpha ) \right] . \end{aligned}$$

Here, each potential function $V_\alpha$ is a function of a subset or clique $C_\alpha$ of the components of ${{{\varvec{s}}}}$, creating a bipartite “factor graph” of variable-nodes indexed by *i* and factor-nodes indexed by $\alpha$ (Lauritzen 1995; Frey 2003). If the factor graph is not connected then its connected components all factorize into independent probability distributions whose product is ${\tilde{p}}$.

To get the dynamics of ${{\varvec{\mu }}}$, one can minimize the KL divergence between ${\tilde{p}}$ and *p*, where ${\tilde{p}}$ evolves under a differential equation

20 $$\begin{aligned} \frac{\mathrm{d} \mu _\alpha }{ \mathrm{d}t} = F_\alpha ( [\mu _\beta |\beta ] ) = F_\alpha ({{\varvec{\mu }}}) \end{aligned}$$

whose right hand sides $[ F_\alpha | \alpha ]$ can be taken to be a learned combination of a large number of hand-designed basis functions (Johnson et al. 2015; Johnson 2012), optimizing a KL divergence between distributions *p* and ${\tilde{p}}$. This is the “Graph-Constrained Correlation Dynamics” (GCCD) model reduction method. It was used to achieve a substantial reduction in number of variables for modeling a molecular complex in synapses.

The goal of training for model reduction may be summarized as minimal degradation over time of the approximation on a set of observables: 
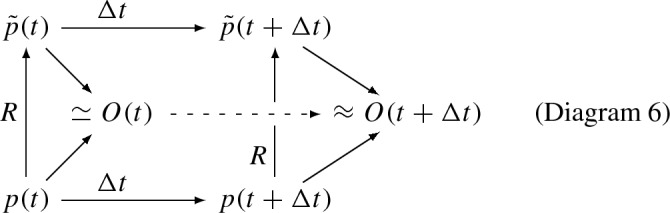
 Here, *R* is a restriction operator mapping fine-scale to coarse-scale states, and ${\Delta } t$ indicates the passage of time under model dynamics. This diagram can be stacked horizontally, for teacher-forcing model training, or vertically, for application across more than two scales. This definition of model reduction is discussed more extensively in Johnson et al. (2015).

This model reduction method has been extended to the case of continuous spatial parameters (Eqs. 6, 8) as described in Supplementary Material Section 7.4.5 (Ernst et al. 2018). In addition, a future direction in model reduction by discrete and/or continuous search over expressions denoting reduced models is introduced in that section as a speculation.

## A Meta-hierarchy for Declarative Modeling

We have developed the ideas of declarative modeling including various formal modeling languages, together with structure-respecting maps (some of them category morphisms or functors) between formal languages and related mathematical objects for semantics, implementation, and model reduction. We have also defined a “meta-hierarchy” as a DAG whose edges are labeled by a DAG of types of relationships, the relationship types forming a specialization hierarchy.

**Fig. 4:**
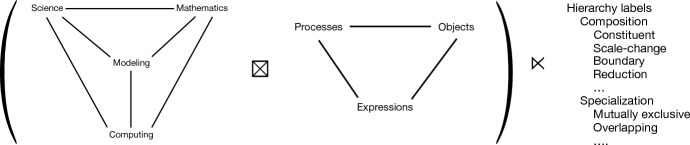
Outline of the Tchicoma meta-hierarchy for organizing formal languages for modeling and the structure-preserving maps between them. First two labeled graphs are fully connected, and the $\boxtimes$ strong graph product between provides for plentiful potential connections. They represent major “knowledge domains” and “ontolexical categories,” respectively. The third element is a DAG of hierarchical relationship types, as called for in the definition of a meta-hierarchy (see main text); this asymmetric construction is indicated idiosyncratically here by the “$\ltimes$” operator. Actual formal languages, and structure-preserving mappings between them, would be positioned deeply inside such a meta-hierarchy—not at the coarse indexing levels illustrated here

**Fig. 5:**
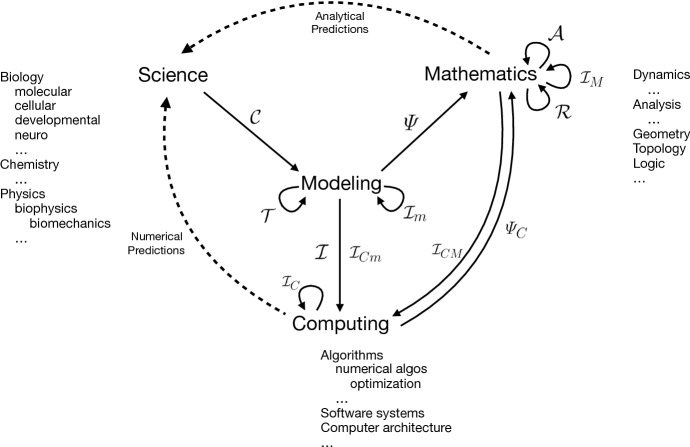
First “knowledge domain” factor of the proposed Tchicoma meta-hierarchy for organizing formal languages involved in complex biological modeling, with graph edge labels showing the *purviews* of the main kinds of structure-preserving mappings between them (${\varPsi }, {{\mathcal {I}}, R, A, C, T}$), as well as a collection of helper mapping types (${\varPsi }_C, \{{{\mathcal {I}}}_*\}$) aimed at computational implementation. Solid arrows represent the purviews of various kinds of mappings; the mappings themselves be would defined individually much deeper in the hierarchy. Mathematically founded implementation maps from modeling to computing should be related by ${{\mathcal {I}}}_{Cm} \simeq {{\mathcal {I}}}_{CM} \circ {\varPsi }$, and/or ${{\mathcal {I}}}_{M} \circ {\varPsi } \simeq {\varPsi }_C \circ {{\mathcal {I}}}_{Cm}$, where defined. Additional mapping kinds could be defined by further such relations. Implementation maps essential for computation can be built up by compositions such as ${{\mathcal {I}}} \simeq {{\mathcal {I}}}_{C} \circ {{\mathcal {I}}}_{Cm} \circ {{\mathcal {I}}}_m$, etc., which may vary in their computational efficiency and domain of applicability. Dotted arrows show workflow information return to science. Semantics maps ${\varPsi }$ apply to modeling languages as discussed in the text; semantics maps ${\varPsi }_C$ apply to the semantics of programming languages and models of computation, as studied in theoretic computer science. The beginnings of a conventional specialization hierarchy for modeling-relevant topics within several knowledge domains are also indicated

We propose here a particular extensible meta-hierarchy (as defined in Sect. 3.3) of formal languages aimed at declarative modeling (as defined in Sect. 2) of complex scientific domains such as developmental biology. We attempt to specify the top levels of the meta-hierarchy (Fig. 4) but leave lower levels free to evolve with usage. Every node in the meta-hierarchy is either a symbolic placeholder (typical for top-level nodes) or represents a formal language or sub-language to be used in a way that satisfies the definition of declarative modeling. The top-level nodes in the meta-hierarchy are symbolic placeholders for a classification of deeper-level nodes along two independent labelings: (a) “ontolexical,” in which the labels are “process,” “object,” and “expression” as used in Sect. 2, and (b) knowledge domain (expanded in Fig. 5), in which the labels are “science,” “mathematics,” and “computing”. In addition there is a “declarative dynamical model informatics” or simply “models” knowledge domain node, aimed at mediating between the other three. Of course many deep sub-classifications are possible especially for the knowledge domains, beginning with specialization links to physics, chemistry, and biology, together with reduction links among their further specializations. The resulting meta-hierarchy is named “Tchicoma” after a volcanic formation in the southern Rocky Mountains.

As discussed in Sect. 3.3, the edges of the meta-hierarchy are labeled by relationship types (e.g., composition vs. specialization, the latter specialized, e.g., into mutually exclusive and/or exhaustive vs. overlapping specializations; also proven vs. machine-verifiably proven vs. unproven relationships; and so on) that themselves stand in a specialization hierarchy. Such relationship links could be used in the construction of maps ${\varPsi }$, ${{\mathcal {I}}}$, and ${{\mathcal {R}}}$ (for semantics, implementation and model reduction, respectively) in declarative modeling, e.g., to retrieve similar known maps from previous work. Specialization links can be used to insert conditions that enable theorems and algorithms to work, and to evolve those conditions as knowledge accumulates. Automatic curation of these link types would also provide an opportunity to keep usage-based statistics on the edges of each type at each node in the meta-hierarchy from prior successful model-building activities, for human visualization, for automatic heuristic search, and for targeting the invention of new nodes in the meta-hierarchy to regions with high previous application. New nodes could be specializations or generalizations of single nodes or of several nodes jointly, resulting in a cumulative resculpting of the meta-hierarchy and its relationship type DAG under pressure of maximal utility.

The languages at the nodes of this meta-hierarchy can be specified by formal grammars, or they can be generated by unary and binary operators defined for all objects in some mathematical category *C*, including but not limited to category-level binary operators such as universal sum, product, or function arrow that can be defined purely in category-theoretic terms. Such a generated language has the advantage that there is a built-in mathematical semantics taking values in the category *C* objects denoted by the operator expression trees. In full generality, the detection of semantic equivalence between expressions in such languages is intractable, though it can often be specialized to a solvable problem.

The near-top nodes of the Tchicoma meta-hierarchy comprise a Cartesian product of three ontolexical nodes and four knowledge domain nodes (Fig. 4). With the resulting twelve-element cross product classification, one can identify the following potential kinds of structure-respecting inter-language mappings (illustrated in more detail in Fig. 5), differentiated by top-level source and target positions in the Tchicoma meta-hierarchy:

1. Mappings discussed in this paper:
  - ${{\varPsi }}$: Semantics: Mapping *from* model object and/or process expressions in a modeling language *L*, *to* mathematical objects (specifically to time-evolution operators, in the case of model processes). This mapping is an essential part of declarative modeling as defined in Sect. 2 and was detailed in Sects. 2 and 3.
  - ${{\mathcal {I}}}$: Implementation: Mapping *from* mathematical objects that are the semantics of model objects and/or model processes, *to* computational objects and processes, respectively; or mapping from model expressions to computational expressions that denote these objects and processes; as introduced in Sect. 3 and illustrated in Diagram 3. Computer Science seeks common target objects for many efficient implementation maps; current examples include Trilinos and PetSc for large-scale scientific computing.
  - ${{\mathcal {R}}}$: Model reduction *from* model expressions *to* model expressions with approximately the same semantics under projection to a set of observables; as exemplified in Sect. 4 and Diagram 6.
  - ${{\mathcal {X}}}$: Transformation: *From* expressions *to* expressions, preserving or approximating semantics (or projections thereof); an essential part of declarative modeling as defined in Sect. 2. For example such transformations could include computational implementation ${{\mathcal {I}}}_m$ at the model level, model translation ${{\mathcal {T}}}$ (discussed below), and/or symbolic model reduction ${{\mathcal {R}}}_m$ = an inverse image of ${{\mathcal {R}}}$ under ${\varPsi }$, if it exists.
2. Mappings not detailed in this paper:
  - ${{\mathcal {C}}}$: Model creation: *From* domain (object, process) expressions in a domain language *to* (object, process) expressions, respectively, in a modeling language. Domain languages may incorporate ontology formalizations such as the Web Ontology Language (“OWL”) or more general Description Logics; examples include the BioPAX biological pathway description language and ontology (Demir et al. 2010), and the Gene Ontology (GO) three-part hierarchy of biological objects and processes that has an object hierarchy and two process hierarchies at two scales, with “Is-A” specialization links within each hierarchy. Declarative model creation packages implemented within computer algebra problem-solving environments include Cellerator (Shapiro et al. 2003, 2015), Cellzilla (Shapiro et al. 2013), and Plenum (Yosiphon 2009).
  - ${{\mathcal {T}}}$: Translation: *From* expressions *to* expressions, preserving or approximating semantics (or projections thereof to observables). For example, translation could formalize the process of translating between the inputs to different modeling software systems. By defining such translations first at the level of classical (and/or intuitionistic) mathematics rather than software, it is much easier to establish equivalences where they exist. Then, one can explore whether and how deeply implementing or updating such translations is worth the effort.
  - ${{\mathcal {A}}}$: Model analysis (phase diagrams, bifurcation diagrams, and the like): *From* models *to* mathematical analysis products such as reduced-parameter spaces, discontinuity or phase-change strata therein, and long-time asymptotics of observables. Model analysis enables further reduction of submodels, for example by algebraic solution of fast subnetworks.

For most of these kinds of maps, several maps of the same kind could have the same source and target nodes (e.g., multiple semantics maps related by refinement as discussed in Supplementary, 7.2.6); in that case subscripting the mapping symbols by *mapping sub-kinds* could become necessary—although one would prefer to elaborate the meta-hierarchy nodes instead, if possible.

The curation of this meta-hierarchy may provide a fruitful application area for automatic theorem verification software based on theorem-proving methods, since (a) many of these map types require the assertion of mathematical equivalences and approximations that could be proven, possibly with computer help, in an automatically verifiable form; (b) the applicability of a particular map to a particular modeling problem could be subject to logical inference on applicability conditions, perhaps using advanced type theory and (c) the retrieval or synthesis of valid map *compositions* that achieve some formalizable goal could be achieved by forward- and/or reverse-chaining style theorem-proving algorithm. For “computing” nodes in the meta-hierarchy, and implementation maps that target them, predictive declarative models of computational resource use as a function of problem statement could also be collected and trained on past data. Heuristic search for useful new intermediate nodes in the meta-hierarchy could be based on the utility of constructing commutative diagrams of inter-node mappings that “lift” one mapping along another, e.g., lifting implementation maps to more mathematical levels of abstraction where possible, in an internal improvement process akin to software refactoring.

## Conclusion

We aim to formalize aspects of mathematical biological modeling so that they become amenable both to computer assistance and to cooperative human development of complex biological models. This capability will be particularly useful in developmental biology, where the necessity of relating genotype to phenotype in any fundamental “evodevo” research program commits a modeler to repeated and often difficult scale-changes within the modeling enterprise. A conceptual framework, based on declarative modeling, in support of these goals is presented. The elements of the conceptual framework include an informal definition of declarative biological modeling with formalized examples; a nested series of declarative biological modeling languages with compatible mathematical semantics defined in terms of operator algebra; a model reduction method based on machine learning with which to tame the often necessary complexity of biological models; and a meta-hierarchy of biological modeling sub-languages and methods, organized and cross-linked by structure-respecting maps.

The biological modeling languages defined in this framework include physics-derived operator algebra semantics for processes expressed as reaction/rewrite rules acting on discrete objects, parameters for such objects that can be discrete and/or continuous variables, extended objects with graph structure including containment and adjacency, and approximate spatially continuous object models. Each of these object types receives appropriate dynamics expressed in reaction/rewrite rules whose operator algebra semantics is built from elementary creation and annihilation operators, so they are compatible and can be mixed together into complex multi-rule models by summation of time-evolution operators. A constructive labeled graph approach to the semantics of extended objects including “graded graphs” labeled by approximation level number and/or stratum dimension and identity can approximate and computably implement constructive variants of classical nonconstructive geometries such as manifolds, cell complexes, and stratified spaces.

In many developmental biology systems, the spatial dynamics involves nontrivial changes in geometry and/or topology of extended biological objects. By using rewrite rules for the graph of strata defined in Sect. 3.1 together ODE-bearing rules for the parametric embeddings of individual strata into 3D space, we now have in principle a way to represent such dynamics mathematically and computationally.

An essential step is to express natural graph-changing operations, including a collection of labeled graph rewrite rules, in terms of an operator algebra generated by the operators for the individual rules. Each graph rewrite rule operator is expressed in terms of elementary creation and annihilation operators, hence explicitly implementable in terms of binary and/or integer-valued random variables. Using this result (Propositions 1 and 2 and corollaries), we also achieve summable operator algebra semantics for modeling languages with rewrite rules at the level of graded graphs, stratified graphs, abstract cell complexes, graded stratified graphs, and/or graded abstract cell complexes that could implement selected continuum limits such as mesh-approximable stratified spaces and cell complexes. A very expressive language of “dynamical graph grammars” results. All rule operator products and commutators are explicitly calculable, enabling the derivation of simulation and analysis methods.

Significant limitations of the approach as discussed here are of course legion and notably include the fact that many graph structures can be defined virtually, as the result of a function of the labels pertaining to two vertices that may or may not be connected, rather than in terms of explicitly represented edges as we have generally assumed.

A potentially generic model reduction and moment closure method for such models is based on dynamically evolving Boltzmann distributions, derived from fine-scale models by a form of machine learning. Other model reduction methods may be enabled by the broad collection of possible mathematical model types that have been formalized as possible outcomes of model reduction. In this way, both numeric (machine learning) and symbolic (declarative model transformations) Artificial Intelligence methods can be brought to bear on complex biological modeling problems.

Finally, an overarching meta-hierarchy of modeling sub-languages is proposed, within which the structure-respecting maps required for declarative biological modeling could be defined, curated, and evolved through experience for maximal utility. This framework may provide opportunities for mathematical biologists to contribute to systematic mappings for complex biological model creation, definition, reduction, implementation and analysis in ways that could be greatly amplified by automation and artificially intelligent computational improvement.

## Electronic supplementary material

Below is the link to the electronic supplementary material.

Supplementary material 1 (pdf 1191 KB)

## Footnotes

1 A simpler published plant model has been translated from declarative form to Organism input; see http://computableplant.ics.uci.edu/sw/CambiumOrganism/.
