# Supplementary material for: Prospects for Declarative Mathematical Modeling of Complex Biological Systems
Source: Bull Math Biol. 2019 Jun 7;81(8):3385–420. doi: 10.1007/s11538-019-00628-7 (PMC6677696; doi:10.1007/s11538-019-00628-7)
Supplement: Supplementary file 1 — Supplementary material 1 (pdf 1191 KB) [file 11538_2019_628_MOESM1_ESM.pdf]

7 Supplementary Material: Prospects for Declarative Mathematical Modeling of Complex Biological Systems

Bulletin of Mathematical Biology

Eric Mjolsness, University of California Irvine, emj@uci.edu

7.1 Notation and Terminology

We will use square brackets to build sets (tuples or lists) ordered by indices, so in a suitable context $f([x]) = f([x_k|k \in \{1, \ldots n\}]) = f(x_1, \ldots x_n)$. Multisets are denoted $\{(\text{multiplicity})(\text{element}), \ldots\}_*$. Indices may have primes or sub-subscripts and are usually deployed as follows: r indexes rewrite rules, i and j index individual domain objects, α and β index domain object types, p and q index elements in either side of a rule, c indexes variables in a parameterized rule, and A indexes a list of defined measure spaces. Object typing will be denoted by ":", so for example $n : \mathbb{N}$ means that $n \in \mathbb{N}$ and the usual set of integer arithmetic operations pertain to n. Subtyping will be denoted by "::".

There is some history, mostly beyond the scope of this paper, to the adaptation of the "declarative/procedural" distinction to both computer programming languages and to formal modeling languages that can be executed on a computer. The classic programming textbook by Abelson and Sussman (Abelson et al. 1996) uses a very similar "declarative/imperative" distinction (knowledge of "what is" vs. "how to") in introducing the programming languages Scheme and Prolog, both with historical pedigrees in logic. Functional programming advocated by Backus (Backus 1978) and exemplified in Haskell, and logic programming exemplified by Prolog, both satisfy "referential transparency" (expressions can be substituted by equivalent expressions or values), a technical idea from mathematical logic that is sometimes taken to define the "declarative" programming paradigm. Even within biological modeling (Spicher et al. 2007) the "what" vs. "how" declarative/procedural distinction has been used, though in a somewhat different way than we use it: in that case the relevant "procedure" to be omitted is fine-grain biological rather than computational process information. We will specialize anew from the looser AI-inspired meaning: Declarative modeling languages specify the goals or criteria of a successful modeling computational simulation, leaving many of the procedural decisions about how exactly to pursue those goals up to other software. This will be done by mathematically specifying the biological (or other scientific) model, including its dynamics.

7.2 Semantics

7.2.1 Explicit operator semantics

If the number of particles of a given species is any nonnegative integer, as we assume for molecular species in solution, then annihilation and creation

operators have infinite dimensional representations and satisfy the commutator relations of the Heisenberg algebra $[a_\alpha, \hat{a}_\beta] \equiv a_\alpha \hat{a}_\beta - \hat{a}_\beta a_\alpha = \delta_{\alpha\beta} I$, where $I = \prod_\gamma I_\gamma$ is the product identity operator over all relevant spaces including $I_\alpha I_\beta$, and δ is the Kronecker delta. For each species α we have the matrix representation in terms of the particle number $n_\alpha \in \{0, 1, \ldots\}$ basis:

$$\hat{a} = \begin{pmatrix} 0 & 0 & 0 & 0 & \cdots \\ 1 & 0 & 0 & 0 & \\ 0 & 1 & 0 & 0 & \\ 0 & 0 & 1 & 0 & \\ \vdots & & & \ddots & \ddots \end{pmatrix} = \delta_{n,m+1} \text{ and } a = \begin{pmatrix} 0 & 1 & 0 & 0 & \cdots \\ 0 & 0 & 2 & 0 & \\ 0 & 0 & 0 & 3 & \\ 0 & 0 & 0 & 0 & \ddots \\ \vdots & & & & \ddots \end{pmatrix} = m\delta_{n+1,m}, \tag{21}$$

and

$$[a, \hat{a}] \equiv (a\hat{a} - \hat{a}a) = I = \begin{pmatrix} 1 & 0 & 0 & 0 & \cdots \\ 0 & 1 & 0 & 0 & \\ 0 & 0 & 1 & 0 & \\ 0 & 0 & 0 & 1 & \\ \vdots & & & & \ddots \end{pmatrix}; \hat{a}a = N \equiv \begin{pmatrix} 0 & 0 & 0 & 0 & \cdots \\ 0 & 1 & 0 & 0 & \\ 0 & 0 & 2 & 0 & \\ 0 & 0 & 0 & 3 & \\ \vdots & & & & \ddots \end{pmatrix}. \tag{22}$$

Note: (Morrison and Kinney 2016) suggest denoting a_α as $\check{a}_\alpha$, in which case $\hat{a}_\alpha$ and $\check{a}_\alpha$ are mnemonic of increasing and decreasing particle number n_α, respectively.

The commutator $[a_\alpha, \hat{a}_\beta] = \delta_{\alpha\beta} I$ can be used to enforce a "normal form" on polynomials in which annihilation operators precede creation operators in each monomial, as in Equation (3). As above we define the number operator $N_\alpha = \hat{a}_\alpha a_\alpha$, diagonal in the "number basis" comprising state vectors $|\boldsymbol{n}\rangle$.

A different case is that in which the number of "particles" of any given species must be in $\{0, 1\}$, as for example if the species are individual binding sites occupied by a particular molecular species; then the operators are 2×2 matrices $a = \left(\begin{smallmatrix} 0 & 1 \\ 0 & 0 \end{smallmatrix}\right)$ and $\hat{a} = \left(\begin{smallmatrix} 0 & 0 \\ 1 & 0 \end{smallmatrix}\right)$ obeying $[a_\alpha, \hat{a}_\beta] \equiv a_\alpha \hat{a}_\beta - \hat{a}_\beta a_\alpha = \delta_{\alpha\beta}(I_\alpha - 2\hat{a}_\alpha a_\alpha)$. Explicitly,

$$\hat{a} = \begin{pmatrix} 0 & 0 \\ 1 & 0 \end{pmatrix}, a = \begin{pmatrix} 0 & 1 \\ 0 & 0 \end{pmatrix} \text{ implies} \tag{23a}$$

$$\hat{a}a = N \equiv \begin{pmatrix} 0 & 0 \\ 0 & 1 \end{pmatrix}, \quad a\hat{a} = Z \equiv I - N = \begin{pmatrix} 1 & 0 \\ 0 & 0 \end{pmatrix}, \text{ and} \tag{23b}$$

$$[a_\alpha, \hat{a}_\beta] = \delta_{\alpha\beta}(I_\alpha - 2N_\alpha)I \qquad \text{Alternative for normal form calcs:} \tag{23c}$$

$$a_\alpha \hat{a}_\beta = \hat{a}_\beta a_\alpha - 2\delta_{\alpha\beta}\hat{a}_\alpha a_\alpha + \delta_{\alpha\beta} I_\alpha \tag{23d}$$

$$= (1 - \delta_{\alpha\beta})\hat{a}_\beta a_\alpha + \delta_{\alpha\beta} Z_\alpha \tag{23e}$$

In this case for each particle species or object type α we can define the diagonal number operator $N_\alpha = \hat{a}_\alpha a_\alpha = \left(\begin{smallmatrix} 0 & 0 \\ 0 & 1 \end{smallmatrix}\right)$, the zero-checking operator $Z_\alpha = I_\alpha - N_\alpha = \left(\begin{smallmatrix} 1 & 0 \\ 0 & 0 \end{smallmatrix}\right)$, and the "erasure" projection operator $E_\alpha = (Z_\alpha + a_\alpha) = \left(\begin{smallmatrix} 1 & 1 \\ 0 & 0 \end{smallmatrix}\right)$ which takes either state to the zero-particle state. In this case also $a^2 = \hat{a}^2 = 0$.

Under Equation (3) one may calculate that $\mathrm{diag}(\mathbf{1}\cdot\hat{W}_r)$ equals the diagonal monomial operator

$$D_r = \prod_{\alpha\in \mathrm{lhs}(r)} {N_\alpha}^{m_\alpha} = \prod_{\alpha\in \mathrm{lhs}(r)} (\hat{a}_\alpha)^{m_\alpha^{(r)}}(a_\alpha)^{m_\alpha^{(r)}} \equiv N^{(r)} = \hat{W}_{\mathrm{LHS}_r\to \mathrm{LHS}_r}, \quad (24)$$

where "$N^{(r)}$" is a number operator for the entire left hand side of the rule, and "LHS_r" is the left hand side of reaction rule r; here in the "$\hat{W}_{\mathrm{LHS}_r\to \mathrm{LHS}_r}$" notation of (Behr et al. 2016) the LHS appears on *both* sides of the arrow. D_r represents the total *probability outflow* from each state under rule r and is, like $\hat{W}_r$, nonnegative in the number basis. One may regard the linearity of Equation (4) as a linear mapping of vector spaces (hence as a morphism or category arrow): The source vector space is spanned by basis vectors that correspond to ordered pairs of multisets of species symbols, weighted by scalar reaction rates k_r, and the target vector space is a vastly larger space of possible probability-conserving operators.

As a consequence of this semantics, two models are *particle-equivalent* just in case they have the same CME solution, i.e. the same joint distribution over all collections of particle numbers n_α observable at the same or different times t. For countable collections (indexed by integer q) these quantities take the equal forms

$$\mathrm{Pr}_{\mathrm{CME}}([n_{\alpha(q)}(t_q)|q]) = \Big\langle \prod_q \delta(N_{\alpha(q)}(t_q) - n_{\alpha(q)}I_{\alpha(q)})\Big\rangle_{\mathrm{CME}} \quad (25)$$

for any choice of particle numbers $\alpha(q)$ and observation times t_q, where: the Kronecker delta is applied componentwise; the "CME" subscript refers to the solution of the Chemical Master Equation, Equation (5) above; and for any diagonal operator D we have at a single time t that $\langle D(t)\rangle_{\mathrm{CME}} = \mathbf{1}\cdot D(t)\cdot p_{\mathrm{CME}}$; unequal times require the joint distribution $\mathrm{Pr}_{\mathrm{CME}}$ at all the relevant times. The $\langle\ldots\rangle$ right hand side expression isn't necessary here but will be useful in a future section. All other observables $\langle f([N_{\alpha(q)}(t_q)|q])\rangle_{\mathrm{CME}}$ (where f is applied componentwise to diagonal matrices) follow from this linear basis, Equation (25).

7.2.2 Parameterized Reaction Rules: Fock Space Semantics Definition

To specify the compositional semantics for *parameterized* rewrite rules it is necessary to specify the probability space in which distributions are defined. In (Mjolsness 2010) a sufficiently general space S of dynamical systems available as targets for the semantics map $\Psi(M) \in S$ is specified in terms of a master equation governing the evolution of a distribution p in a Fock space constructed out of elementary measure spaces for the parameters.

The semantic map $\Psi(M)$ involves each species of molecule, cell type, or other object type α in a way that depends on the maximum number $n_\alpha^{(\max)}$ of indistinguishable individuals possible for that species at a time. Here as in Equation (21) we will assume n_α is unbounded ($n_\alpha^{(\max)} = +\infty$). This case is

relevant to well-stirred chemical reaction network models in which a harmless simplifying assumption is that there is no fixed upper bound to the number of indistinguishable instances of a particular object. However, the $n_\alpha^{(\max)} = 1$ case (as in Equation (23)) will be relevant to extended object modeling below, both for spatially localized objects and for graph-like extended objects.

In order to extend the main continuous-time model semantics $\Psi(M)$ to define the semantics of a model containing not only objects but also dynamical *variables*, each generally taken to be associated with some object, we will need to integrate over the possible values of all such variables. Notation is as follows. As before, r indexes rewrite rules and α and β index domain object types. Also p and q index elements in either side of a rule, c indexes variables in a rule, and A indexes a list of defined measure spaces. Each variable X_c will have a type required by its position(s) in the argument list $[x_p]$ of one or more terms $\tau_{\alpha(p)}$, and a corresponding measure space $D_{A(c)}$ and measure $\mu_{A(c)}$. Here A indexes some list of available measure spaces including discrete measure on $\mathbb{N}$ and Lebesgue measure on $\mathbb{R}^{d:\mathbb{N}}$.

Let $\mathcal{V}_\alpha = \bigotimes_p D_{A(\alpha,p)}$ be the resulting measure space for parameter lists of terms of type α. To summarize briefly the "symmetric Fock space" construction outlined in (Mjolsness and Yosiphon 2006; Mjolsness 2010): For each nonnegative integer n_α we define a measure space of states that have a total of n_α "copies" of parameterized term $\tau_\alpha(x_\alpha)$:

$$f_\alpha(n_\alpha) = \left(\bigotimes_{m=1}^{n_\alpha} \mathcal{V}_\alpha \right) / \mathcal{S}(n_\alpha) \tag{26}$$

where $\mathcal{S}(n)$ is the permutation group on n items - in this case the elements of the n_α measure space factors. Next, any number $n_\alpha : \mathbb{N}$ of terms is accommodated in a disjoint union of measure spaces $f_\alpha(n_\alpha)$, resulting in a measure space for all terms of type α, and a cross product is taken over all species α:

$$f_\alpha = \bigoplus_{n_\alpha=0}^{\infty} f_\alpha(n_\alpha) \quad \text{and} \quad \mathcal{F} = \bigotimes_\alpha f_\alpha \quad . \tag{27}$$

This is the measurable system state space. In it, objects of the same type α are indistinguishable except by their parameters $x_p : \mathcal{V}_\alpha$.

The operator semantics now becomes an integral over all the variables X_c:

$$\begin{aligned} \hat{W}_r = \int \dots \int_{[D_c|c]} \left(\prod_k d\mu_{A(c)}(X_c) \right) \rho_r \Big([x_p([X_c])] \, , [y_q\left([X_c]\right)] \Big) \\ \times \left\{ \prod_{q \in \mathrm{rhs}(r)} \hat{a}_{\beta(q)} \left(y_q([X_c])\right) \right\} \left\{ \prod_{p \in \mathrm{lhs}(r)} a_{\alpha(p)} \left(x_p([X_c])\right) \right\} . \end{aligned} \tag{28}$$

This expression is again in normal form, with annihilation operators preceding (to the right of) creation operators. When other operator expressions need to

be converted to normal form one uses the same kinds of Heisenberg algebra commutation relations as before except that the $\delta_{\alpha\beta}$ Kronecker delta functions are now augmented by Dirac delta functions, and their products, as needed to cancel out the corresponding measure space integrals:

$$\begin{aligned} [a_\alpha(\boldsymbol{x}), \hat{a}_\beta(\boldsymbol{y})] &= \begin{cases} \delta_{\alpha\beta}\delta_{\mu_A}(\boldsymbol{x}-\boldsymbol{y})I & \text{for } n \in \mathbb{N} \\ \delta_{\alpha\beta}\delta_{\mu_A}(\boldsymbol{x}-\boldsymbol{y})(I_\alpha - 2N_\alpha(\boldsymbol{x}))I & \text{for } n \in \{0,1\} \end{cases} \\ f(\boldsymbol{x}) &= \int_A \mu_A(\boldsymbol{y})\delta_{\mu_A}(\boldsymbol{x}-\boldsymbol{y})f(\boldsymbol{x}-\boldsymbol{y}) \end{aligned} \tag{29}$$

The integrals over measure spaces act a bit like quantifiers in first order logic, binding their respective variables. We speculate that the product of two such operator expressions could be computed in part by using the logical unification algorithms of computational symbolic logic, since the problem of finding the most general unifier (MGU) arises naturally when integrating over several sets of variables during restoration of canonical form (cf. the proof of Proposition 2 in Section 7.4.4 below) using the commutation relations of Equation (29) and their delta functions. Such full MGU computations (Martinelli et al. 1982) may incur overhead costs which however are not large if the computation is performed on the relatively small AST representing the model as part of model analysis or implementation, rather than at simulation time when the graphs are large and there is a premium on speed.

7.2.3 Parallelism in operator algebra semantics

In the Wightman axioms for quantum field theory (QFT) the physical characteristics of *locality* and *causality* enter through commutation relations similar to Equation (29) pertaining to spatiotemporal fields that can be built out of creation/annihilation operators (Glimm and Jaffe 1981). What is important is that fields at points with spacelike separation (in our case, that includes fields defined at the same time t, omitted in our notation, but different places $\mathbf{x}$ and $\mathbf{y}$, as shown in Equation (29) above) must commute. In this way different processes, or processes acting on different objects, all happen truly in parallel. When continuous models of space are added to the semantics our modeling languages, e.g. differential equations as discussed in Sections 2.3 (main text) and 7.2.8 below, it will be important to ensure that they too respect causality. Hyperbolic PDEs clearly do so; parabolic PDEs comprise a borderline case.

In simulation, the mapping from model time to computer time can in principle be done by slicing space-time along any family of spacelike surfaces, including but not limited to surfaces of constant model time, while maintaining the parallelism due to commutation of operators at spacelike separation as determined by any propensity functions and differential operators in the model. This geometry provides a natural limit to the parallelism of discrete event simulations. Thus, the operator algebra *of the rules* of a model, developed further in Section 7.4.4 below, specifies the amount of computational parallelism possible for a given model - usually high since the natural world being modeled is intrinsically parallel.

7.2.4 Brief comparison to L-Systems and BioNetGen

A related fully declarative modeling language family rides under the banner of "L-systems", named after Lindenmeyer and championed by Prusinkiewicz (e.g. (Prusinkiewicz and Lindenmeyer 1990)). L-systems and their generalizations have been effective in the modeling of a great variety of developmental phenomena, particularly in plant development, because of their declarative expressive power. But the usual semantics of L-systems falls outside the family of languages considered in this section and in Section 3.2.1, for a theoretically interesting reason. The applicable rules of L-systems are usually defined to fire in parallel, and *synchronously in discretzed time* so that one tick of a global clock may see many discrete state updates performed. This semantics seems to be incompatible with the *summation* of local, continuous-time operators defined in Equation (4), because "atomic" uninterruptible combinations of events are represented in operator algebra by multiplication rather than by addition of operators (and even then they are serialized), and on the other hand continuous-time parallel processes are represented by addition rather than multiplication of operators, in the master equation that describes the operation of processes in continuous time. Despite this difference, one could seek structure-respecting mappings between operator algebra and L-system semantics at least at the level of individual rule-firings.

A subtle point here is that the truly (to very high accuracy at least[2]) continuous-time parallelism and compositionality of the physical universe seems to be best expressed as in QFT with spacelike commutation of operators (as in Equation (29)) and by Equation (4)'s summation of time-evolution operators over processes and (as shown in the next section) over space. In terms of this elementary parallelism, it takes further engineering and/or computing to implement synchronous discrete-time parallelism in terms of such continuous-time parallelism. Modeling languages that invoke such relatively "heavy" discrete-time parallel semantics at the rule-firing level include traditional L-systems, MGS with maximal-parallel or alternative semantics (Maignan et al. 2015), the subgrammar call feature of SPGs and Dynamical Grammars, and the optimization-based definition of rule-firing choice in the development-modeling grammars of (Mjolsness et al. 1991). Each such semantics implicitly poses an interesting problem of efficient reduction to continuous-time parallel semantics, in general or for specific biological models.

A relevant point of comparison for the semantics of parameterized reaction network languages is the BioNetGen modeling language (Blinov et al. 2004). This language has been applied to many problems in signal transduction pathway modeling with discretely parameterized terms representing multistate molecular complexes. It also represents labelled graph structures that arise in such molecular complexes, placing it also in the class of graph rewrite rule dynamics languages.

[2] Physical time can now be measured to one part in 10^{18} (McGrew et al. 2018), so any discretization of time must be finer than this

7.2.5 Comments on differential equation rules

Differential equation bearing rules such as Equation (8) with the semantics of Equation (9) can be used to describe processes of growth and movement of individual particle-like objects, as in “agent-based” modeling. For example, a cell may grow according to a differential equation and divide with a probability rate (propensity) that depends on its size, as in the plant root growth model of (Mironova et al. 2012). In general, rewrite rules can be used to describe individual processes within a model if they are augmented (as above) with a symbolically expressed quantitative component such as a probability distribution or a differential equation. Each such process has a semantic map Ψ to an algebra of operators, and processes operating in parallel on a common pool of objects compose by operator addition (Equation (4)). Declarative computer languages based on this and other chemical reaction arrows, transformed to ordinary differential equation deterministic concentration models, include (Shapiro et al. 2015b, 2003; Mjolsness 2013; Yosiphon 2009) among others.

The semantics as developed so far covers discrete-time transitions between parameterized objects, stochastically interrupting continuous-time semantics given by differential equations. As such it is similar to “hybrid systems” with discrete events indicated by threshold crossings, and indeed that is one possible implementation for the ODE portion of a dynamical grammar solution engine (Mjolsness 2013), the hybrid SSA/ODE solver, in which a “warped time” variable increases until a predefined randomly chosen maximum warped time when the next rule fires. However, there are several increases in generality in the present framework. The discrete events occur stochastically according to ODE-state-dependent time-varying propensities, and can be specialized to behave deterministically; the converse is not generally true. The continuous-time ODE dynamics does not occur in a single continuous product space over parameters, but rather in a space of intrinsically varying dimension, because discrete events change the number and nature of parameterized objects (as in Mjolsness et al. 1991); we refer to this kind of dynamical system as a “variable-structure system”. In addition the semantics have been generalized to cover true continuous-time stochastic processes, such as Brownian motion, specified by stochastic differential equations.

7.2.6 Refining semantic maps

We have outlined how rule-like syntax can be mapped to operator algebra semantics in several cases, though not yet for extended objects in sufficient generality (cf. Section 3). There is also the possibility to define several interrelated semantics maps for one modelling language, in order to serve purposes such as analysis and computation. In (Mjolsness 2013, Section 3.1.2) the operator algebra/ master equation semantics of Equation (4) and Equation (5) for the case of stochastic chemical kinetics was also related to a discrete-“timestep” Markov chain semantics equivalent to the Gillespie Stochastic Simulation Algorithm, in which the molecular state and the most recent reaction time determine the

molecular state and physical reaction time just following the “next” reaction event on a computational time axis. (This relationship is discussed further in Section 7.4.1 below.) The discrete timesteps in the Markov chain map to reaction event number k, not to a uniform discretization of continuous physical time t. Since the distribution of *intervals* between reaction times depends only on molecular state, this Markov chain can be projected further down to a Markov chain without reaction time state information (Equation (39)) - determining what happens but not when. But of course the additivity of time-evolution operators $W = \sum_r W_r$ doesn't map to additivity of Markov chains U and in this sense the continuous-time model must be primary.

Starting with such a model Markov chain which they refer to as “stochastic semantics” for the “Biochemical Abstract Machine” (Biocham) modeling language, (Fages and Soliman 2008) show that one can project systematically down to yet courser semantics for chemical reaction networks such as a “discrete semantics” related to Petri nets which forgets transition rates and hence conflates nonzero probabilities, and a “Boolean semantics” which tracks only zero vs. nonzero molecule number for each chemical species. These coarse semantic maps are formalized as in programming language theory by way of a “Galois connection” between two lattices, namely adjoint forward and reverse order-preserving functions. In the case of discrete-time models there is a large literature on programming language semantics to draw on for this purpose; much of it uses denotational semantics based on lattice theory, although operational semantics (Plotkin 2004) is another relevant approach. In programming language theory, process algebras such as the “Calculus of Communicating Systems” (Milnor 1980) are designed to have a clear mathematical semantics for parallel computational processes. In general it is useful and interesting to be able to formally map a mathematical model semantics Ψ to a computational model semantics Ψ_C (a mapping discussed further below) because the latter can be *implemented* in a conventional programming language; the resulting implementation mappings could be a formally verified computer program implementing a mathematical model. Formal verification can help assure not only correctness, but also computational efficiency by making available program transformations for efficiency that are too involved for human programmers to make at with a reasonable level of effort.

7.2.7 Definitions related to graphs and graph homomorphisms

Discrete graphs, especially when augmented with labels, are mathematical objects that can represent computable objects and expressions at a high level of abstraction.

7.2.7.1 Standard definitions

An (undirected/directed) *graph* is a collection V of nodes and a collection E of edges each of which is an (unordered/ordered) pair of vertices. We allow self-edges (for example by defining an “unordered pair” to be a multiset of cardinality 2). A *graph homomorphism* is a map from vertices (also called nodes) of

source graph to vertices of target graph that takes edges (or links) to edges but not to non-edges. Graphs (undirected or directed) and their homomorphisms form a category. The category of undirected graphs can be modeled within the category of directed graphs by a functor representing each undirected edge by a pair of oppositely directed edges between the same two vertices.

Either category of graphs and graph homomorphisms contains resources with which to formalize *labelled graphs* (specifically vertex-labelled graphs), *bipartite graphs*, and *graph colorings* all as graph homomorphisms (to a fully connected and self-connected graph K_{Λ}^{+} of $L = |\Lambda|$ distinct labels $\Lambda = \{\lambda_{i\in\{1...L\}}\}$; to a two-node graph without self-connections; and more generally to a *clique* of L distinct nodes fully interconnected but without self-connections, respectively). Edge-labelled graphs (as opposed to the default vertex-labelled graphs) can be modelled by way of a functor from graphs to bipartite graphs which maps vertices to red vertices and edges to blue vertices; the resulting bipartite graph can be further labelled as needed. In a *multigraph*, the collection E of edges is allowed to be a multiset rather than just a set; thus a single (unordered/ordered) pair of vertices in V can be connected by any nonnegative integer number of edges, not just zero or one.

As usual, a *tree* is an undirected graph without cycles and a Directed Acyclic Graph (DAG) is a directed graph without directed cycles. A *directed rooted tree* is a DAG whose undirected counterpart is a tree, and whose (directed) edges are consistently directed away from (or consistently towards) exactly one root node. Then Abstract Syntax Trees (ASTs) (previously discussed as the substrate for declarative model transformations; see Figure 1) can be represented as node- and edge-labelled directed rooted trees with symbolic labels for which the finite number of children of any node are totally ordered (e.g. by extra numerical edge labels), so there is a unique natural "depth-first" mapping of ASTs to parenthesized strings in a language. Alternatively, ASTs can be mapped to terms in universal algebra, facilitating abstraction. Representing an AST as a labelled tree has the additional advantage that one or more graph rewrite rules (Section 3.2.1) can be defined to manipulate the ASTs so as to implement the transformations of model-denoting ASTs foreseen in the informal definition of declarative modeling langages in Section 2. In addition to depth-first traversal, it is also possible to totally order the nodes in a tree or DAG in "breadth-first" ways that respect the directed-edge partial order, as in task scheduling.

The automorphism group of a labelled graph is in general reduced in size from that of the corresponding unlabelled graph, making it computationally easier to detect particular subgraphs. Some binary operators of type $(\text{graph}, \text{graph}) \rightarrow \text{graph}$, namely categorical disjoint sum "$\oplus_{\text{Graph}}$" and the graph cross product "$\times$" = categorical product "$\otimes_{\text{Graph}}$", can be defined by the usual universal diagrams applied to the Graph category. The graph product "$G \times H$" connects a pair of product vertices $(a, b), (c, d)$ just in case a connects to c in G *and* b connects to d in H. If G and H happen to be two-node graphs each with a single edge between their nodes, the resulting graph (we could call it a "pictograph") looks like the symbol "$\times$", where a and c are

laid out along the horizontal axis and b and d along the vertical. The graph box product "$\square$", essential for meshing, can also be formulated as a tensor product universal diagram but not strictly within the category ((Knauer 2011) Theorem 4.3.5). The graph box product "$G \square H$" connects a pair of product vertices $(a, b), (c, d)$ just in case a connects to c in G *and* b is identical to d in H, *or* vice versa. If G and H happen to be two-node graphs each with a single edge between their nodes, the resulting pictograph looks like the symbol "$\square$". The "$\boxtimes$" strong graph product includes all edges licensed by either $\times$ *or* $\square$ (Imrich and Klavžar, 2000), and again the product symbol is close to denoting its own pictograph. Graph *functions* by binary operator "$\rightarrow_{\text{Graph}}$" that produces a graph are also possible but require an altered definition of the category (Brown et al. 2008).

7.2.8 Non-standard definitions

We consider graph homomorphisms to five particular integer-labelled graphs:

$$
\begin{aligned}
\mathbb{N}^+ \equiv & (\mathbb{N}, \text{Successor}) \\
& = \text{ nonnegative integers } \{0, 1, \ldots\} \text{ as vertices,} \\
& \text{with (possibly directed) edges from each} \\
& \text{integer } i \text{ to its immediate successor } i+1 \text{ and to itself;} \\
J_D^+ \equiv & \begin{cases} (\mathbb{N}_D \equiv \{0, \ldots D \geqslant 0\}, \geqslant) & \text{directed graphs;} \\ \quad = \text{ integers } \{0, \ldots D\} \text{ with } (i,j) \text{ edge iff } i \geqslant j; & \\ K_{\mathbb{N}_D}^+ = K_{\{0,\ldots D\}}^+ \text{ (fully connected w. self-edges)} & \text{undirected graphs} \end{cases} \\
\mathbb{N}_D^{+\text{op}} \equiv & \begin{cases} \text{integers } \{0, \ldots D\} \text{ with } (i,j) \text{ edge iff } i = j+1 \text{ or } i = j & \text{directed graphs;} \\ \text{integers } \{0, \ldots D\} \text{ with } (i,j) \text{ edge iff } |i-j| \leqslant 1 & \text{undirected graphs} \end{cases} \\
C_D \equiv & \mathbb{N}^+ \square J_D^+ \\
\tilde{C}_D \equiv & \mathbb{N}^+ \square \mathbb{N}_D^{+\text{op}}
\end{aligned}
\tag{30}
$$

(In this notation the '+' exponents G^+ refer to the addition of self-edges.) Graph homomorphisms to these graphs will provide the core of the next few definitions. Following ((Ehrig et al. 2006) Chapter 2), such homomorphisms themselves form useful graph-related categories. These definitions can all be used in either the undirected or directed graph contexts. The two contexts are related by the standard functor in which an undirected edge corresponds to two oppositely directed edges, defining a mapping from undirected graphs to directed graphs.

As in the main text, a *graded graph* is defined as a homomorphism from a graph G to $\mathbb{N}^+$. It labels vertices by a level number. Here we note also that a conflicting definition for the term "graded graph", disallowing connections within a level, was given in (Fomin 1994) but we need both $\Delta l = 0$ and $\Delta l = +1$ edges. In the category of undirected graphs, $\Delta l = \pm 1$ are indistinguishable

except by consulting the vertex labels l. Of course, $(\mathbb{N}^+, id_{\mathbb{N}^+})$ is itself a graded graph.

Pursuing the discussion of stratified graphs in the main text, another useful special case of a stratified graph occurs if G_S obeys the constraint that (a) only adjacent dimensions connect. In that case one has the discrete graph structure which we have defined as an abstract cell complex (ACC), although there are several related claimants for that phrase and we make no claim for the superiority of this one. This condition results from using the Hasse diagram for the dimension-labelled strata, with other possible boundary relationships among cells being obtained by multiple steps along $\Delta d = -1$ ACC edges. In most geometric applications one may in addition observe that (b) two strata of dimensionality labels $d-1$ and $d+1$ are always mutually adjacent to either zero or two strata of dimension d (Lane 2015), though in common with the "abstract complexes" of MGS (Giavitto and Michel 2001, Giavitto and Spicher 2008) we do not make this part of the definition. ACCs have been used for declarative developmental modeling at least of plant development (Lane 2015) and neural tube development (Spicher 2007), the latter using MGS.

7.2.9 Details on graph grammar rule operator semantics

In the graph grammar rule operator semantics of Equation (13) we note that the set Λ of available labels λ can be augmented with an extra unique formal label $\varnothing$ to make the full label set $\Lambda^+ = \Lambda \,\dot\cup\, \{\varnothing\}$. Then we can enforce a unary "winner-take-all" (WTA) or "one-hot" encoding by coupling each Λ label addition or removal with an opposite change of state for the $\varnothing$ label: $a_{i,\lambda} = \hat{b}_{i,\varnothing} b_{i,\lambda}$, $\hat{a}_{i,\lambda} = \hat{b}_{i,\lambda} b_{i,\varnothing}$. After using $b_\alpha^2 = 0 = \hat{b}_\alpha^2$, and since b operators with different indices commute, the form of Equation (13) is preserved by this mapping. Under it, inductively every vertex i will have exactly one label $\lambda \in \Lambda^+$ present, $n_{i\lambda} = 1$; this constrains the subspace that the dynamical system will move through. A vertex i is regarded as "allocated" or "active" if any of its label states $(i, \lambda \in \Lambda)$ are present ($n_{i\lambda} = 1$ for some $\lambda \in \Lambda$); otherwise not; in other words, if it has $n_i = \sum_{\lambda \in \Lambda} n_{i\lambda} = 1$ rather than zero. Since $\sum_{\lambda \in \Lambda} n_{i\lambda} + n_{i,\varnothing} = 1$ is an invariant of dynamics using this WTA encoding, then for a (partly or completely) unlabelled graph the presence of the extra "$\varnothing$" label will indicate a vertex is inactive.

In addition to the main four factors in the graph grammar rule operator semantics of Equation (13), we now consider allocation and deallocation of integer-valued graph vertex indices i from a single central index list. The leading (rightmost) operator could be preceded by a rightmost memory-checking "pre-factor" such as $X_r = \Big[\prod_{p \in \mathrm{rhs}(r) \setminus \mathrm{lhs}(r)} Z_{i_p \lambda_p} \Big]$ to ensure that only currently unused memory gets allocated for new use, but since $\hat{a}_{i\lambda} Z_{i\lambda} = \hat{a}_{i\lambda}$ and $\hat{a}_{i\lambda}$ is present in the second line and unobstructed by $a_{i\lambda}$ in the third line whenever it is present in this pre-factor, and since we are in sector $\mathcal{S}_{\mathrm{graph}}$ with at most one label per node activated, such a pre-factor is not needed.

The indices i_p should be each averaged rather than summed over, since it doesn't matter *which* unused node is brought into service; hence the proportionality rather than equality in (13). If only a finite range of index values $i_p \in \{0, \ldots I_{\text{overflow}} - 1\}$ are possible and they are all used up, then only rewrite rules that don't create new nodes will be able to fire. One is naturally interested in the infinite storage space limit $I_{\text{overflow}} \equiv N_{\text{globalmax}} \to +\infty$ for which this problem doesn't occur; in that case one could argue that the E post-factors (in right to left order) become unimportant since for any finite state of the graph rewriting system, the chance of accidentally reusing a node approaches zero. Here however we will conservatively retain the E post-factors on line 1. These trailing (leftmost) "garbage collection" erasure operators $\left(\prod_i E_{i_p\, i} E_{i\, i_p}\right)$ erase any edges (i_p, i) or (i, i_p) dangling from or associated with nodes i_p that have just been deleted (factor $a_{i_p \lambda}$ on the third line), making those nodes suitable for reuse in future rule firings. An erasure factor $E_{i_p \lambda_p}$ is not needed since $E_\alpha a_\alpha = a_\alpha$, and $a_{i_p \lambda_p}$ is present on line 3 and unobstructed by $\hat{a}_{i_p \lambda_p}$ (for $p \in \text{rhs}(r)$) on line 2 whenever it would be present on line 1.

These erasure operators maintain the invariance of the statements that (a) every vertex has either 0 or 1 labels present (1 defining an "active" vertex), and (b) edges that are present must connect two active vertices. In operator-state language the predicate S_{graph} is:

$$\forall_i \quad \Big(\sum_{\lambda \in \Lambda} N_{i\lambda}\Big) |\boldsymbol{n}\rangle = n_i\, |\boldsymbol{n}\rangle, \quad n_i \in \{0, 1\} \tag{31a}$$

$$\forall_{i,j} \quad N_{ij}\, |\boldsymbol{n}\rangle = n_{ij}\, |\boldsymbol{n}\rangle, \quad n_{ij} \leqslant n_i n_j \tag{31b}$$

(Here the label space Λ could be designed as a product space $\Lambda = \otimes_A \Lambda_A$, so no intrinsic expressivity is lost under condition (a)). We assume these invariant statements are true of the initial condition; for example they are true in the "vacuum" state in which all $n_* = 0$, and of course in any other state reachable from the vacuum by one or more grammar rule firings. These conditions S_{graph} define a linear subspace "sector" $\mathcal{S}_{\text{graph}}$ of the Fock space $\mathcal{F}$. Starting from the null space $|\boldsymbol{n} = \boldsymbol{0}\rangle$, this sector is preserved by the operators $\hat{W}_r$. The exact form of the X, E pre- and post- factors (though we don't use X here), and the invariant predicate $\mathcal{S}_{\text{graph}}$ they maintain, can be formulated in several different ways that differ just by the hidden indices i_p that end up representing particular labelled graphs.

We may calculate $D_r \equiv \text{diag}(\boldsymbol{1} \cdot \hat{W}_r)$ for Equation (13) using $\boldsymbol{1} \cdot E = \boldsymbol{1}$, where G_r is the labelled graph on the LHS of rule r:

$$D_r = N^{(G_r)} \equiv \sum_{\langle i_1, \ldots i_k \rangle_{\neq}} \Bigg[\prod_{p \in \text{lhs}_r} N_{i_p\, \lambda}\Bigg]\Bigg[\prod_{p,q \in \text{lhs}(r)} \left(N_{i_p i_q}\right)^{g_{p\, q}}\Bigg]. \tag{32}$$

Equivalence of models, previously defined by "particle equivalence" under master equation semantics in Equation (25), must be modified for this graph grammar semantics to account for the fact that the index numbering i_p of graph vertices is unimportant; only their labels and edges matter. Any

permutation of indices should yield an equivalent state, as should any subpermutation which also alters the choice of which indices are active, holding fixed their number. So we would like to define "equivalent" models in an index subpermutation-invariant way. Subpermutation invariance (with respect to indexing) is achieved by using the index-invariant number operators $N^{G(q)}$ of Equation (32) for each labelled graph G_q in any collection indexed by q (usually taken to be finite or at least countable), and then seeking equality of all joint probabilities:

$$\Pr_{\text{graph}}([n_{(G_q)}(t_q)|q]) = \left\langle \prod_q \delta(N^{(G_q)}(t_q) - n_{(G_q)}(t_q) I_{G(q)}) \right\rangle_{\text{graph}}, \qquad (33)$$

just as in Equation (25), but with graph number operators now defined in the index-subpermutation-invariant manner of Equation (32). As before, other graph grammar observables $\langle f([N^{(G_q)}(t_q)|q]) \rangle$ can be defined from these, where any function f acts on the components of a diagonal matrix (which is consistent with any polynomial expansion or power series f may have). The resulting equivalence relation on labelled graph grammar models could be called *graph-equivalence.* As a further elaboration of the graph case, graph-equivalence could be restricted to some sector $\mathcal{S}$ of the Fock space, preserved under time evolution of a model, for example $\mathcal{S}_{\text{graph}}$ defined above. By a natural extension, two operators are graph-equivalent if they can be added to a third operator in common, yielding two graph-equivalent model time-evolution operators.

Closely related concepts have been proposed recently by (Behr et al. 2016) and nicely re-expressed, *without* an explicit analog of our grounding and hence implementation map $\mathcal{I}$ in terms of elementary creation/annihilation operators $\hat{a}, a$, but instead more abstractly. As in (Mjolsness 2010), they suggest that Fock spaces and master equations built from a collection of rule-level operators generalizing the Heisenberg algebra creation/annihilation operators could express graph rewrite rules. They introduce operator representations similar to our number basis by way of many graph-counting "observable" operators $\hat{W}_{\text{LHS}_r \to \text{LHS}_r}$ $(= N^{(\text{LHS}_r)} = D_r$ in our notation - see Corollary 2 below). They also derive differential equation dynamics for graph moments in terms of other, generally higher order moments, which raises the classic moment closure problem of statistical mechanics. (Behr et al. 2016) also derive their operator construction from the "double pushout" category-theoretic approach to defining graph grammar semantics, which we will discuss briefly in Section 7.2.10 below.

By comparison, in this work we express $\hat{W}_r$ in terms of products of elementary creation and annihilation operators and thus provide an explicit implementation in terms of Boolean and/or integer-valued random variables. This implementation map (a) enables the integration of graph rewrite semantics with non-graph modeling language semantics defined in previous sections; (b) enables the mechanical computation of fundamental commutation relations for graph rewrite rules as in Propositions 1 and 2 and the Corollaries below, thus in principle permitting the derivation and analysis of operator splitting simulation methods underwritten by e.g. the Baker-Campbell-Hausdorff (BCH)

theorem; and (c) supports the derivation of model reduction based moment closure methods such as those of Section 4. In addition we include labels for the graph vertices and show how other categories of spatially extended objects can be mapped to and hence implemented in terms of such labelled graphs.

As is the case for Equation (3), Equation (13) can be taken as a normal form for rewrite rule dynamics, but now applied to graphs. We will show next that (somewhat akin to the "Concurrency theorems" of the double pushout approach discussed in Section 7.2.10 below, but more general) the product and commutator of two such forms can be rewritten as a (possibly large) integer-weighted sum of expressions having the same form, or a form of equivalent meaning with extra factors of E that don't affect the active node set; however, some of the weights may be negative.

7.2.10 Pushout semantics

A very different approach to defining a similar idea of graph rewrite rule semantics is provided by the "double pushout" category-theoretic construction (Ehrig et al. 2006), using the category of graphs and graph transformations.

A pure (unlabelled) graph grammar rule $G \to_K G'$ can replace graph G with graph G', holding common subgraph K constant, anywhere that G (and its subgraph K) occurs as a subgraph inside of some "host" graph C within the current pool of one or more graphs (indeed C can be taken to be the entire pool of graphs in the current state since that's just a big, possibly disconnected graph). The result will be an altered version C' of the host or pool graph. Using graph homomorphism arrows, the double pushout diagram for the firing of a graph grammar rule relates all these graphs as follows:

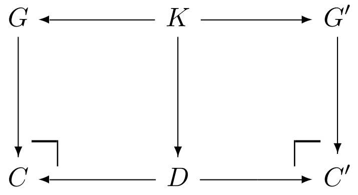

(Diagram 4)

The diagram is to be universal at C and C' (so any rival occupant for either position is essentially just a homomorphic image of the universal occupant), hence is double pushout. If such a C' exists, then it is a candidate outcome for the effect of a rule firing of the given rule on the given pool.

What is nice about this diagram is that, as in Section 3.1, its objects and arrows can be reinterpreted in other graph-related categories including all the foregoing slice categories such as labelled graphs, graded graphs, stratified graphs, abstract cell complexes, and their various combinations in Section 7.2.8 above; and any other typed attributed graphs. We also imagine that it should be possible to *implement* a slice graph category rewrite rule under the double pushout semantics in terms of ordinary (double pushout) labelled graph rewrite rule firings, by use of suitable labels. A possible sticking point is verifying implementation of the universalities of the pushout construction

in the slice category. In this paper we only studied the analogous question for operator algebra semantics (Section 3.2.3).

There is also a closely related single pushout diagram version of the semantics, and a collection of “independence” conditions for two successive rule firings to have an order-independent result (Ehrig et al. 2006). The work by (Behr et al. 2016) discussed above combines in one paper and connects together both double-pushout and master equation semantics, using a restricted subset of the operator algebra implied by Propositions 1 or 2. Their work provides evidence in favor of some version of the following idea:

Conjecture 1 Since the operator algebra and pushout diagram semantics are alternative ways of defining the “same” operation, they must be related. So we conjecture there is a map Ψ_2 making Diagram 5 commute:

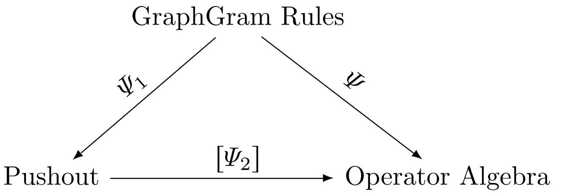

(Diagram 5)

The conjectured semantics reduction mapping Ψ_2 can be defined as tranferring probability to the subspace of states of the Fock space corresponding to a C' post-firing pool graph, from the subspace of states of the Fock space corresponding to a C pre-firing pool graph. The operator algebra of Propositions 1 and 2 and its corollaries, possibly different in detail from that of (Behr et al. 2016), is in turn mapped by an implementation map $\mathcal{I}$ to a computational substrate including stochastic simulation algorithms.

7.3 Biological Examples

7.3.1 Stem cell niche examples, amplified

To further explain the 1D root growth model cited in the main text, the cell division rule (one out of about a dozen in the model “grammar”) was augmented to preserve 1D topology in a manner similar to the following:

$$
\begin{array}{l}
\text{/* cell replication, explicitly preserving 1D structure: */} \\
\mathrm{cell}[\mathrm{curr}, 2, x, r, A, Y, \mathrm{prev}, \mathrm{next}], \mathrm{cell}[\mathrm{prev}, \mathrm{mode}', x', r', A', Y', \mathrm{prevprev}, \mathrm{curr}] \\
\quad \mathrm{cell}[\mathrm{next}, \mathrm{mode}, x'', r'', A'', Y'', \mathrm{curr}, \mathrm{nextnext}] \\
\longrightarrow \mathrm{cell}[\mathrm{new}_1, 1, x - r\alpha, r(1-\alpha), A, Y, \mathrm{prev}, \mathrm{new}_2], \\
\quad \mathrm{cell}[\mathrm{new}_2, 1, x + r(1-\alpha), r\alpha, A, Y, \mathrm{new}_1, \mathrm{next}], \\
\quad \mathrm{cell}[\mathrm{prev}, \mathrm{mode}', x', r', A', Y', \mathrm{prevprev}, \mathrm{new}_1], \\
\quad \mathrm{cell}[\mathrm{next}, \mathrm{mode}, x'', r'', A'', Y'', \mathrm{new}_2, \mathrm{nextnext}] \\
\textbf{with} \quad \rho_Y(Y/Y_0) p_\alpha(\alpha) \quad /* \text{ where } p_\alpha(\alpha) \text{ enforces } \ \alpha \in [\tfrac{1}{2} - \varDelta, \tfrac{1}{2} + \varDelta] \quad */
\end{array}
\tag{34}
$$

Here $p_\alpha(\alpha)$ denotes a uniform distribution of α in its allowed interval, governing the relative sizes (lengths) of daughter cells compared to the parent cell.

Also A (the plant growth hormone auxin) and the hypothetical molecule Y are two dynamical morphogens; “curr”, “next”, “prev”, etc. are unique (e.g. integer-valued) object identifiers. Cell positions are denoted by $x, x', \ldots$, and $r, r', \ldots$ denote 1D cell radius i.e. half of cell length. Variable “mode” is a discrete cell state determining readiness for cell proliferation vs. vegetative growth; it is determined stochastically in another rule by cell size r compared to a threshold. The discrete “mode” parameter could be obviated by the use of subtyping, in which some rules such as biomechanics act on all “cell” objects and other only on “vegetative_cell::cell” or on “proliferative_cell::cell” subtypes which under the Liskov substitution principle of programming languages would each be subject to the generic cell rules as well. Random variable $\alpha \in [\frac{1}{2} - \Delta, \frac{1}{2} + \Delta] \subseteq [0, 1]$ denotes the fraction of parent cell size inherited by one of the two daughter cells; the other gets $1 - \alpha$. Constant parameters include Y_0, a baseline level of Y, and Δ, the allowed variation of α away from $\alpha = 1/2$ which would represent spatially symmetric cell division.

There are many other cell lineage tree systems in biology in which less committed cell types give rise to more committed cell types during development by symmetric or and/or asymmetric cell division. Examples include vertebrate hematopoiesis, and the generation of neuronal diversity in animal brain development (Holguera et al. 2018). For example early vertebrate neural development could be modeled by cell division/specialization rules of the general form:

$$
\begin{aligned}
\text{apical\_radial\_glia}(\boldsymbol{g}_1) &\rightarrow \text{intermediate\_basal\_progenitor}(\boldsymbol{g}_2), \\
&\quad\;\; \text{apical\_radial\_glia}(\boldsymbol{g}_3) \;\textbf{with}\; \rho_{\text{aia}}(\boldsymbol{g}_1, \boldsymbol{g}_2, \boldsymbol{g}_3) \\
\text{intermediate\_basal\_progenitor}(\boldsymbol{g}_1) &\rightarrow \text{neuron}(\boldsymbol{g}_2),\;\; \text{neuron}(\boldsymbol{g}_3) \;\textbf{with}\; \rho_{\text{inn}}(\boldsymbol{g}_1, \boldsymbol{g}_2, \boldsymbol{g}_3) \\
\text{apical\_radial\_glia}(\boldsymbol{g}_1) &\rightarrow \text{outer\_radial\_glia}(\boldsymbol{g}_2),\;\; \text{outer\_radial\_glia}(\boldsymbol{g}_3) \\
&\qquad \textbf{with}\; \rho_{\text{aoo}}(\boldsymbol{g}_1, \boldsymbol{g}_2, \boldsymbol{g}_3) \\
\text{outer\_radial\_glia}(\boldsymbol{g}_1) &\rightarrow \text{outer\_radial\_glia}(\boldsymbol{g}_2),\;\; \text{neuron}(\boldsymbol{g}_3) \\
&\qquad \textbf{with}\; \rho_{\text{oon}}(\boldsymbol{g}_1, \boldsymbol{g}_2, \boldsymbol{g}_3) \\
&\ldots
\end{aligned}
\tag{35}
$$

in which the relative rates of conflicting rules (e.g. the first and third rules above) are determined by transcriptional regulation (gene expression level vectors $\boldsymbol{g}_i$) which can change upon cell division rule firings (as formalized for example in (Mjolsness Sharp Reinitz 1991)) in accordance with some propensity functions $\rho_*(\boldsymbol{g}_1, \boldsymbol{g}_2, \boldsymbol{g}_3)$, unless of course one chooses to model $\rho_*(\boldsymbol{g}_1, \boldsymbol{g}_2, \boldsymbol{g}_3) = \rho_*(\boldsymbol{g}_1)\delta(\boldsymbol{g}_2 - \boldsymbol{g}_1)\delta(\boldsymbol{g}_3 - \boldsymbol{g}_1)$ so that gene expression doesn't change upon cell division. Further continuous-time rules would allow all cell expression vectors to evolve under transcriptional regulation between cell division events.

7.3.2 2D SAM quantitative cell division rules

For a two-dimensional model, one would like more than a single random variable α to describe the selected geometry of a particular cell division. A variety

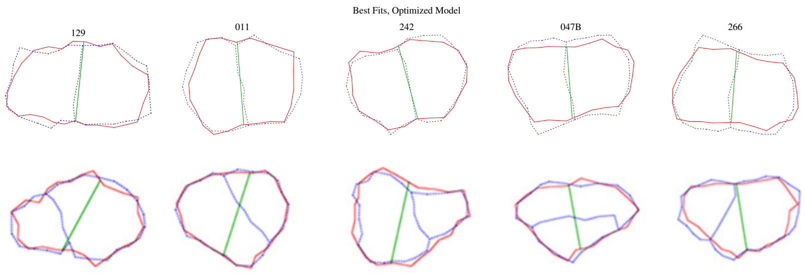

Fig. 6 (A) Top row: Best fits within a data set of cell images, after model optimization. (B) Bottom row: Corresponding worst fits within the same data set of cell images. Reprinted from (Shapiro et al. 2015a).

of cell-scale phenomenological "rules" have been explored for this geometrical behavior in plant cells, including rules proposed historically by Hofmeister, Errera, and Sachs. A parameterized family of probabilistic rules based on the Boltzmann distribution $p_\theta(\boldsymbol{\theta}|\mathbf{w}) = \exp\left[-\beta E(\boldsymbol{\theta}|\mathbf{w})\right]/Z(\beta)$ for phenomenological energy functions $E(\boldsymbol{\theta}|\mathbf{w}) = \sum_{i \in \text{area,length,extension,growth}} w_i V_i(\boldsymbol{\theta})$, encompassing modern interpretations of these historical rules as points $\mathbf{w}$ in a larger parameter space, was explored in (Shapiro et al. 2015a). Any such a Boltzmann distribution could easily be placed in a cell division rule comparable to the foregoing 1D rule, in place of the factor $p_\alpha(\alpha)$. The form chosen had several parameters learned from relevant microscopy data for the shoot apical meristem (SAM) (the opposite end of the plant from root apical meristem) of the genetic model plant *Arabidopsis thaliana*; the optimal rule was closest to but somewhat better than the standard modern interpretation of Errera's rule. Some realistic stochastic variation was captured (see Figure 6), as indicated by a relatively small but nonzero optimal temperature parameter in the Boltzmann distribution. Both rules were implemented in the declarative 2D cellular tissue modeling package "Cellzilla" (Shapiro 2013), and created growing convex polygonal patterns that appear qualitatively similar to those of derived from microscope imagery of SAM tissue as shown in Figure 3.

7.3.3 MT dynamics details

See Figures 7 and 8, illustrating plant cortical microtubule network dynamical processes, as described in the main text.

A textual presentation of a dynamic graph grammar (DGG) that includes several of these processes is here:

$$
\begin{aligned}
\mathrm{grow\_end}[\mathrm{curr}, \boldsymbol{x}, \boldsymbol{u}, S_{\mathrm{in}}, \varnothing] &\longrightarrow \mathrm{internal}[\mathrm{curr}, \boldsymbol{x}, \boldsymbol{u}, S_{\mathrm{in}}, \{\mathrm{new}\}], \\
&\quad\ \mathrm{grow\_end}[\mathrm{new}, \boldsymbol{x} + \boldsymbol{\Delta x}, \boldsymbol{u}', \{\mathrm{curr}\}, \varnothing], \\
&\quad\ \textbf{with}\ \ \hat{\rho}_{\mathrm{grow}}([\mathrm{tubulin}])\mathcal{N}(\boldsymbol{\Delta x}; L\boldsymbol{u}, \sigma) \\
&\qquad \times \mathcal{N}(\boldsymbol{u}'; \boldsymbol{u}/(|\boldsymbol{u}| + \epsilon), \epsilon), \\
\mathrm{retract\_end}[\mathrm{prev}, \boldsymbol{x}_{\mathrm{p}}, \boldsymbol{u}_{\mathrm{p}}, S_{\mathrm{in}}, \{\mathrm{curr}\}], & \\
\mathrm{internal}[\mathrm{curr}, \boldsymbol{x}, \boldsymbol{u}, \{\mathrm{prev}\}, S_{\mathrm{out}}] &\longrightarrow \mathrm{retract\_end}[\mathrm{curr}, \boldsymbol{x}, \boldsymbol{u}', \varnothing, S_{\mathrm{out}}], \\
&\quad\ \textbf{with}\ \ \hat{\rho}_{\mathrm{retract}} \\
\mathrm{internal}[\mathrm{prev}, \boldsymbol{x}_{\mathrm{p}}, \boldsymbol{u}_{\mathrm{p}}, S_{\mathrm{in}}, \{\mathrm{curr}\}], & \\
\mathrm{internal}[\mathrm{curr}, \boldsymbol{x}, \boldsymbol{u}, \{\mathrm{prev}\}, \{\mathrm{next}\}], & \\
\mathrm{internal}[\mathrm{next}, \boldsymbol{x}_{\mathrm{n}}, \boldsymbol{u}_{\mathrm{n}}, \{\mathrm{curr}\}, S_{\mathrm{out}}], & \\
\mathrm{grow\_end}[\mathrm{sport}, \boldsymbol{x}, \boldsymbol{u}_{\mathrm{s}}, S'_{\mathrm{in}}, \varnothing] &\longrightarrow \mathrm{internal}[\mathrm{prev}, \boldsymbol{x}_{\mathrm{p}}, \boldsymbol{u}_{\mathrm{p}}, S_{\mathrm{in}}, \{\mathrm{curr}\}], \\
&\quad\ \mathrm{junct}[\mathrm{curr}, \boldsymbol{x}, \boldsymbol{u}, \{\mathrm{prev}, \mathrm{sport}\}, \{\mathrm{next}\}], \\
&\quad\ \mathrm{internal}[\mathrm{next}, \boldsymbol{x}_{\mathrm{n}}, \boldsymbol{u}_{\mathrm{n}}, \{\mathrm{curr}\}, S_{\mathrm{out}}], \\
&\quad\ \mathrm{internal}[\mathrm{sport}, \boldsymbol{y}, \boldsymbol{u}_{\mathrm{s}}, S'_{\mathrm{in}}, \{\mathrm{curr}\}] \\
&\quad\ \textbf{with}\ \ \hat{\rho}_{\mathrm{bundle}}(|\boldsymbol{u} \cdot \boldsymbol{u}_{\mathrm{s}}|/|\cos\theta_{\mathrm{crit}}|) \\
&\qquad \times \exp(-|\boldsymbol{x} - \boldsymbol{y}|^2/2L^2) \\
\mathrm{retract\_end}[\mathrm{prev}, \boldsymbol{x}_{\mathrm{p}}, \boldsymbol{u}\mathrm{p}, S_{\mathrm{in}}, \{\mathrm{curr}\}], & \\
\mathrm{grow\_end}[\mathrm{curr}, \boldsymbol{x}, \boldsymbol{u}, S_{\mathrm{in}}, \varnothing] &\longleftrightarrow \varnothing \\
&\quad\ \textbf{with}\ (\hat{\rho}_{\mathrm{retract}}, \hat{\rho}_{\mathrm{nucleate}}([\mathrm{tubulin}])) \\
&\qquad \mathcal{N}(\boldsymbol{x}; \mathbf{0}, \sigma_{\mathrm{broad}})\delta_{\mathrm{Dirac}}(|\boldsymbol{u}| - 1) \\
\mathrm{grow\_end}[\mathrm{curr}, \boldsymbol{x}, \boldsymbol{u}, S, \varnothing] & \\
&\longleftrightarrow \mathrm{retract\_end}[\mathrm{curr}, \boldsymbol{x}_{\mathrm{p}}, \boldsymbol{u}, \varnothing, S] \\
&\quad\ \textbf{with}\ (\hat{\rho}_{\mathrm{growth}\to\mathrm{retract}}, \hat{\rho}_{\mathrm{retract}\to\mathrm{growth}})
\end{aligned}
\tag{36}
$$

Here the notation "$\mathrm{grow\_end}[\mathrm{curr}, \boldsymbol{x}, \boldsymbol{u}, S_{\mathrm{in}}, \varnothing]$" is equivalent to "$\bullet[\mathrm{curr}, \boldsymbol{x}, \boldsymbol{u}, S_{\mathrm{in}}, \varnothing]$" or to "$\mathrm{segment}[\bullet, \mathrm{curr}, \boldsymbol{x}, \boldsymbol{u}, S_{\mathrm{in}}, \varnothing]$", etc.. The parameter [tubulin] is the concentration of tubulin dimer, here taken to be a constant but in general dynamic. Parameter ϵ ($0 < \epsilon \ll 1$) represents a small amount of stochastic "wobble" in the direction vector $\boldsymbol{u}$ per increment $\approx L$ in MT length; approximately unit vectors $\boldsymbol{u}$ are renormalized before use in the first rule, with protection against division by zero. Spatial step standard deviation parameter σ could be on the order of $L\epsilon$. Notation $\mathcal{N}(x; \mu, \sigma)$ denotes a vector

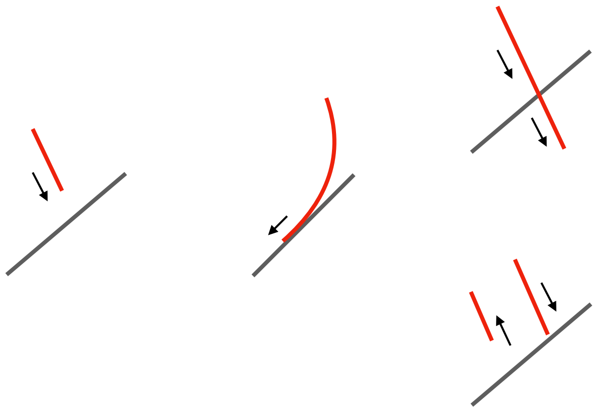

Fig. 7 Microtubule (MT) discrete transition rules associated with the collision of two MTs confined to a nearly two dimensional environment such as the cortex of a cell just inside the cell membrane. Left: cortical MT (red) undergoing "+ end" growth (thin arrow) approaching the static middle section of another cortical MT or MT fiber (gray). Center: At low angles of incidence (Wightman and Turner 2007), the approaching MT may preferentially zipper or bundle into a fiber. Right: At higher angles of incidence, the approaching MT can cross over the static one (top right) possibly forming a junction, or (bottom right) it may undergo a state change to catastrophic depolymerization at the + end. The choice may be modeled as stochastic. Redrawn and modified from (Chakrabortty et al., 2018).

Gaussian or Normal distribution for vector x with vector mean μ and standard deviation σ with covariance matrix proportional to the identity. The last two rules use bidirectional arrows as shorthand notation for a pair of ordinary unidirectional rules.

A key feature of this dynamical graph grammar is that a large number of growth and/or retraction steps (essentially, MT treadmilling in bundles) are expected to occur in between any of the events that change the number or nature of MTs: collision-induced junction formation, or MT birth or death in the fourth (bidirectional) rule. These first two MT length-altering but not MT number-altering rules form an solvable subsystem - analytically solvable as a recursion equation in the case of flat 2D geometry and noise $\epsilon \to 0$, otherwise a form of random walk which is also tractable analytically and easy to sample numerically. So the system decomposition $W_{\mathrm{MT}} = W_{\mathrm{treadmilling}} + W_{\mathrm{birth/death/bundling}}$ can be approached numerically by the (operator-algebraic) TOPE method mentioned in Section 2.3. In the zero noise limit, one could approximate the treadmilling graph grammar rules by differential equation rules in a different graph grammar that for example omits the interior nodes entirely but adds real-valued length parameters functions to one or both end nodes. This method would properly consider the effect of time-varying treadmilling velocities and other time-dependent propensities. If in addition the treadmilling velocities are constant then the straight line MT trajectories can be projected analytically to their intersections in space and time, which would

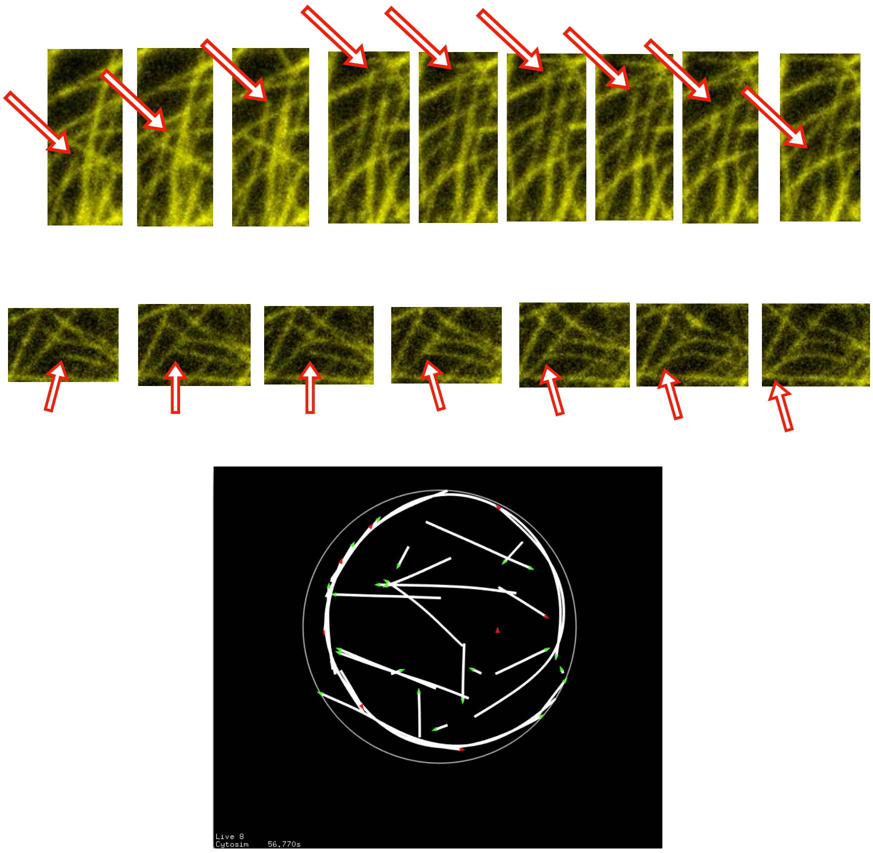

Fig. 8 (A)-(B): Sequences of image windows from an *Arabidopsis thaliana* pavement cell cortical microtubule microscopy time series. (A) An image sequence interpretable as a collision-induced catastrophe following several crossovers. (B) An image sequence interpretable as collision, then a short "withdrawal" or a large image noise event, then regrowth followed by stable zippering. (A-B): Thanks to Ray Wightman, Sainsbury Laboratory Cambridge University for help with sample preparation and microscopy, using a Leica TCS SP8 confocal laser scanning microscope in resonance scanning mode, with *Arabidopsis* samples containing fluorescently labelled microtubules as described in (Wightman and Turner 2007). (C) A larger window into a frame of a movie created in Cytosim (Nedelec et al. 2007) exhibiting MT zippering (confluence of green arrows) and collision-induced catastrophe (red arrows), along with stochastic MT biomechanics, among other processes. Thanks to Dustin Maurer, UCI for this simulation.

result in an event-driven simulation algorithm similar to that of (Tindemans et al. 2014).

7.3.4 Mesh refinement graph grammar

We discussed how to refine a 2D triangular mesh with a minimal (labelled-) graph grammar, with Equation (10) as one of four rules needed. Of the other

three grammar rules, two are analogs of (10) for two or three hanging side nodes like node 4 above; only one rule increases the maximal level number:

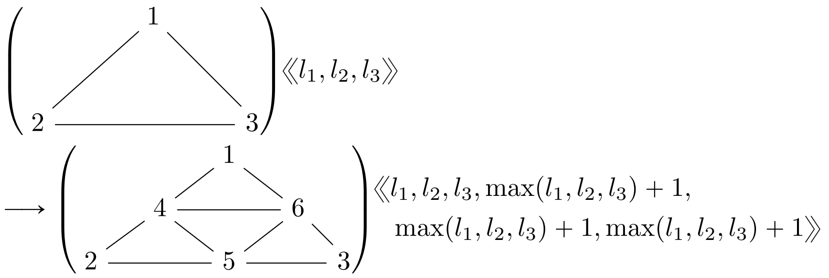

(37)

In the resulting four-rule grammar, the order in which triangles are refined does not interfere with the potential refinement of other triangles. If for example a global maximum-level constraint $l \leqslant L$ were imposed via the firing rate of each rule, then the refinement process would converge by many alternative paths to the same fully refined terminal triangular mesh.

A more complicated rewrite rule can maintain not only l = level number but also d = dimension in a cell complex representation, during this kind of triangle refinement.

To implement a 2D version of the the polyhedron → cuboid mesh refinement scheme mentioned in Section 7.4.3 below, one could alternatively start with four triangle → quadrilateral refinement rules; for example instead of rule (10) one would have:

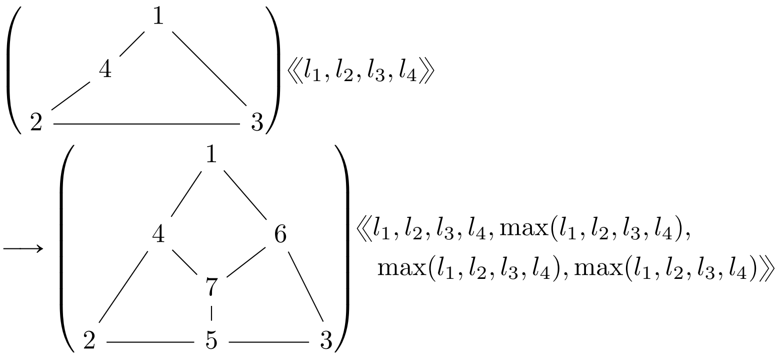

(38)

However, one is then committed to designing a larger grammar that can refine both triangles and quadrilaterals with varying numbers of hanging side nodes like node number 4 above, possibly requiring six more quadrilateral refinement rules since there are two ways to place two such side nodes. Abstraction via sub-grammars may then become important. Further notational innovations could also reduce the number of rules needed.

7.4 Mathematical points

7.4.1 Symbolic and numeric solutions of elementary operator algebra dynamics

A classic solvable example (McQuarrie 1967) is the well-mixed chemical reaction network $A \rightleftharpoons B$, with forward and reverse rates k_f and k_r respectively. The time-evolution operator for this system is defined on the space $\mathbb{N}_A \otimes \mathbb{N}_B$ by

$$W_{A \rightleftharpoons B} = k_f(\hat{a}_B a_A - N_B) + k_r(\hat{a}_A a_B - N_A).$$

This operator is a sum of four summed monomial terms: two for the forward reaction and two for the reverse. The first term destroys a particle of type A and immediately creates a replacement particle of type B. The second term removes the same amount of probability per unit time from the current state. Likewise for terms three and four. Reversibly unimolecular systems like this, with one molecule in and one molecule out of each reaction, can be solved analytically by treating each molecule in the system as an independent one-particle system. Alternatively one can use generating functions to solve most or all of the solvable small networks.

A systematic operator-algebraic solution of this example proceeds by (a) representing probability distributions with generating functions and mapping creation and annihilation operators to multiplication by variables z_α and derivative operators ∂_α (for $\alpha \in \{A, B\}$) respectively, obtaining a PDE; (b) separating out the time variable by seeking solutions proportional to $\exp \lambda t$; (c) using conservation laws and initial conditions (here $n_A + n_B =$ constant) to reduce from two integer state variables and two generating function variables to one, e.g. $\zeta = z_A/z_B$; (d) analytically solving the resulting differential equation (sometimes only possible for the steady state, $\lambda = 0$); (e) impose the initial condition $g(t = 0) = \prod_\alpha z_\alpha^{n_\alpha(t=0)}$ obtaining in our particular case a convolution of two binomial distributions that converge to one equilibrium binomial distribution. For systems that are not analytically solvable, such as $A + B \rightleftharpoons C$, there may be an analytically tractable approximation for dynamics not only in the limit but also in the approach to the limit of large numbers of molecules, for example by approximating the most significant eigenvalues and eigenvectors of W using boundary layer theory (Mjolsness and Prasad 2013).

Associated to the continuous-time semantics $\Psi(W)$ is a *discrete-time semantics* $\Psi_d(W)$ (Mjolsness and Yosiphon 2006):

$$\Pr(k) = U \circ ...U \circ \Pr(0) \equiv U^k \circ \Pr(0), \quad \text{for} \quad k \in \mathbb{N} \tag{39}$$

where U is related to W below. In the present treatment we will simplify matters by assuming there are no terminal states, so the diagonal matrix D can be inverted by inverting its elements. We can define the first rule-firing update at step $k : \mathbb{N}$ in a Markov chain as follows:

$$p_{k+1} = \tilde{W} \cdot D^{-1} \cdot p_k \tag{40}$$

where in this simplified case $\tilde{W}$ is just equal to $\hat{W}$. This update is now in a form that can be iterated as a linear map $U = \tilde{W} \cdot D^{-1}$, and hence can be iterated and interpreted as a stochastic algorithm as in Equation (39). The full treatment including terminal states, and the projection meta-operator from continuous-time semantics to discrete-time semantics $\Psi_d(W)$, is given in (Mjolsness and Yosiphon 2006).

7.4.2 Nonconstructive extended objects

Classical mathematics is not constrained by the requirement of being computationally constructive, though it can be. This lack of constraint makes easier to establish useful mappings and equivalences (compared for example to Intuitionist mathematics), so it is easier to reuse what is already known in proving new theorems. We would like to retain these advantages in a theoretical phase of work before mapping to constructive computer simulations.

Classical mathematical categories such as topological spaces, measure spaces, manifolds, CW cell complexes, and stratified spaces (the latter two composed of manifolds of heterogeneous dimension) provide models of extended continuum objects such as biological cell membranes, cytoskeleton, and tissues made out of many adjacent cells. Function spaces such as various Hilbert and Banach spaces can be used to provide models of definable biophysical ields (such as concentrations and biomechanical stress/strain fields) whose values vary over such extended objects. Objects in such categories may (depending on the category) have rich and useful collections of k-ary operators (unary, binary, countable associative, etc. object-valued “operators”, not to be confused with the probability-shifting dynamical creation/annihilation operators of Section 2) such as category sums and products, and even function arrows for Cartesian Closed Categories such as compactly generated topological spaces (Steenrod 1967; Booth and Tillotson 1980). Such $\oplus_C, \otimes_C$, and $\rightarrow_C$ operators for category C can be targeted by compositional semantics from context-free grammar rules that generate expressions including these operators in an AST in a modeling language; these are essentially “type constructor” type inference rules in standard programming language semantics (Pierce 2002). In principle, further invocations of category-specific function-arrow type constructors $\rightarrow_C$ could find application by way of variational calculus (whose “functionals” are functions from functions to reals) and even higher-order variational calculus; the latter has recently been applied to reaction-diffusion models in the model reduction work outlined in Section 4 and described in detail in (Ernst et al. 2018).

Further object-generating operators may require mathematical objects in heterogeneous but related categories, such as defining new submanifolds by level sets of continuous functions using the regular value theorem (related to the implicit function theorem), or alternatively as the image of a continuously differentiable embedding ([Hirsch 1976] Chapter 1, Theorems 3.1 and 3.2.). Such level set functions could be biophysical fields such as concentration of morphogen for tissue domain boundary (as in the well-known French flag

model (Wolpert 1969) for locally representable spatial information in developmental biology), or cortical microtubules (as in Section 2.2.1) in the preprophase band whose placement can predict the Cortical Division Site for plant cell division, or they could be purely mathematical phase fields that rapidly interpolate between discrete values for different compartments.

With extended objects we encounter the possibility that the *type* of one object is itself another typed *object*. For example a point $x : \tilde{S}$ may be constrained to lie on the surface of a sphere $\tilde{S}$: Manifold($d = 2$) :: Manifold which is itself of type 2-dimensional manifold or more generally a Manifold. (As an example, a membrane-bound receptor may diffuse in the 2D membrane of a cell which could be modeled as a manifold homeomorphic to the 2-sphere $\tilde{S}$.) Indeed this possibility may be taken to define an "extended" object like $\tilde{S}$: It is, or it at least informs, the type of its sub-objects. For consistency one might like to map biological domain objects to "mathematical objects" that find their formal packaging in category theory by the same definition, whether they are constituent objects like point x or extended objects like 2D manifold $\tilde{S}$. There are several ways to do this but one way is by loose analogy with Homotopy Type Theory (HTT 2013): We may define a *mathematical object* x as an object in a category, if for each topological space T (such as $\tilde{S}$) modeling an extended object (such as a topologically or even geometrically spherical model of the membrane of a protoplast) we also define an associated category whose category-objects are the individual *points* of T (itself a category-object in Top or Manifold) that might model, for example, positions of diffusible membrane-bound receptors. Then as in HTT, continuous paths in a space are morphisms between its points. An enriched category results, based in our case on homotopy of manifolds, CW complexes, and stratified spaces as well as graph path homotopy.

By such means we could nonconstructively define the mathematical semantics map Ψ of an extended object model in terms of functors (or at least structure-representing functions) to classical mathematical categories. However the mathematical objects in these spaces are not generally defined in a computable way, so we can't get all the way to computable implementations by this route.

Our strategy to achieve computability will be to define another fundamental kind of map in addition to semantics maps Ψ: namely, *Implementation* maps $\mathcal{I}_*$ (for various subscripts $*$ to be discussed). Implementation maps $\mathcal{I}_{CM}$ go from restricted versions of potentially nonconstructive but standard mathematical categories, to labelled graphs. They enable the construction of implementation maps $\mathcal{I}$ from models to labelled graphs and their computable dynamics, by composition with Ψ, which is most of what we need to run a model on a computer. The restricted version of standard mathematical spaces could for example be something like piecewise linear stratified spaces (Weinberger 1994), or low-order polynomial splines to better preserve the differential topology of manifolds and stratified spaces ((Hirsch 1976) Chapter 3; (Weinberger 1994) Part II). By abuse of terminology such implementation maps may alternatively run from language (with classical mathematics semantics) to lan-

guage (with graph semantics), provided the corresponding semantics diagram commutes. In other words, implementation should commute with the semantic map, both respecting composition. This constraint applies to $\mathcal{I}$ both as it acts on extended objects, and on the process models (e.g. differential equations or Markov chains) under which they evolve. Diagram 3 in Section 3.2.3 will provide examples. More implementation maps are discussed in Figure 5 of Section 5.

Finite extended objects can be modeled by labelled graphs defined in the next section. These are eminently computable. Infinite graphs comprise a borderline case. One can use *sequences* of interrelated, finite, computable graphs which we will formalize as “graded graphs” to approximate spatial continua. (These are similar to the “graph lineages” together with inter-level “prolongation maps” defined in (Scott and Mjolsness 2019).) In this way we can have access to continuum extended objects as declarative modeling “semantics” for very fine-grained biological objects, while “implementing” these infinite mathematical objects in terms of explicitly finite and computable ones. Of course one’s computational resources may or may not be adequate to making the approximation needed.

A major goal for declarative modeling of continuum objects is declarative modeling of partial differential equations (PDEs), before they have been spatially discretized into e.g. ordinary differential equation systems. Software that already supports some PDE models declaratively, though not in conjunction with all the other process types of Section 2 or 3.2, includes the “Unified Form Language” language for specifying PDEs through their algebraic weak forms (Alnaes et al. 2014), in the well-developed FEniCS project (Logg et al. 2012) for finite element method (FEM) numerical solution of PDEs, leaving the detailed choice of finite element solution methods to other parts of the model specification; and a computer algebra system (Wolfram Research 2017) that similarly separates an algebraic model specification from the choice of solution algorithms. Of course the choice of numerical solution methods for PDEs is a vast area of research; here we just observe that methods for which the mapping from continuum to discrete descriptions (e.g. meshing) is dynamic and opportunistic, such as Discontinuous Galerkin methods generalizing FEMs, or Lagrangian methods including particle methods for fluid flow, would place extra demands on the flexibility of the graph formalism for extended objects introduced in Section 3.1.

7.4.3 Dynamics on graphs

Given an extended object G constructed by graph grammar rules, it is generally necessary also to define some dynamics that run “in” or “on” such an object: diffusing or otherwise moving particles with position $x \in G$ described by a spatial probability distribution $p(x, t)$, or other dynamical fields $f(x)$ at a given moment of time t. To this end, recall that the inverse image of a node in G_S identifies the corresponding stratum in G. It is natural to use the graph Laplacian on each such stratum to define: (a) geometric distances, using

the Green's function of the Laplacian operator; (b) regularizers for regression of functions from sparsely provided data (cf. Poggio and Girosi (1990)); (c) a Sobolev space H^2 of functions in the infinite-graph limit, for biophysical fields; (d) the definition of *functional integrals* in the infinite-graph limit, using a kernel $k_{m,\lambda}(x, y) = (m - \lambda\nabla^2)\delta_{\mathrm{Dirac}}(x - y)$ to form a statistical mechanics partition function such as the Gaussian functional integral (e.g. Mandl and Shaw (2010)):

$$\begin{aligned} Z[m, \lambda, J] &= \int D[f] \exp\left[-\frac{1}{2}\int\int dx dy f(x) k_{m,\lambda}(x, y) f(y) + \int dx J(x) f(x)\right] \\ &= \int D[f] \exp\left[-\frac{1}{2}\int dx[m f(x)^2 + \lambda(\nabla f(x))^2] + \int dx J(x) f(x)\right]; \end{aligned} \tag{41}$$

and/or (e) Graph Convolutional Networks, a generalization of deep convolutional neural networks for machine learning from rectangular grids to general graphs (Hammond et al. 2011, Kipf and Welling 2016). But in order to recover the expected continuum properties from the graded graph of meshes associated with a stratum, it may be necessary to reweight the edges of the graph (and hence its Laplacian) according to the local scale of its embedding into for example the three dimensions of flat biological space. Laplacians and their heat kernels on Riemannian manifolds are sufficient to recover the local geometric structure by way of triangulated coordinate systems (Jones et al. 2008). Graph heat kernels converge to the manifold ones in cases where a sequence of graphs "approximate" the manifold (Coifman and Lafon 2006; cf. Mjolsness and Cunha 2012).

If some of the strata in the graph of strata G_S are host to partial differential equations (respecting possibly dynamical boundary conditions at adjacent lower and higher dimensional strata) then G_S will have to be subdivided and meshed before solution algorithms like finite elements or finite volumes can be applied. Ideally the strata of G_S should be subdivided sufficiently finely into patches that can each host a local coordinate system, compatible (conforming) with its neighboring patches of different dimension and (under one possible strategy) separated by extra artificial boundary strata patches from its neighbors of the same dimension. Compatible finer meshes (computed eg. by programs such as Tetgen ((Si and Gartner 2005); illustrated for plant tissue by (Mjolsness and Cunha 2012)) could be aligned with the local coordinate systems. As a simpler example, a cell complex graph whose cells embed into 3D as polyhedra can be subdivided into cuboids whose main diagonals each stretch from a vertex of the starting polyhedron to its centroid; but this decomposition may or may not have good numerical properties such as condition number of a meshed mechanical stiffness matrix. A problem for developmental biology, as for fluid simulation, is to maintain mesh quality while the system simulated undergoes large deformations. For example very close to corner-like boundaries it may become hard to avoid mesh cells with extreme angles and poor numerics, but conforming meshes that fence off such boundaries at short

distance from them (Rand and Walkington 2009, Murphy et al. 2001, Engwirda 2016) together with analytical PDE dimension reduction to such surfaces may provide an alternative path forwards. Meshing for PDEs is a vast field of applied mathematical research to which we cannot do justice; the point here is that stratified graded graphs (and perhaps other graph slice categories) provide a way to formalize many of the problems and capabilities that have to be represented explicitly for declarative modeling to be applied.

Abstract cell complex graphs are also the key structure for Finite Element Methods (Hughes 2000), and for Discrete Exterior Calculus (DEC) discretizations of PDEs (Desbrun et al. 2005) and the related Finite Element Exterior Calculus (FEEC) (Arnold et al. 2010). DEC and FEEC allow for the separate discrete representation of k-forms and the full exploitation of the generalized Stokes' theorem, including symplectic PDE integrators and the Helmholtz/Hodge decomposition of function spaces for possible PDE solutions. DEC can be combined with subdivision (de Goes et al. 2016) in the manner of a graded stratified graph. (Giavitto and Spicher 2008) show how to encode oriented abstract cell complexes and discretized differential operators similar to DEC in the MGS declarative modeling language. On the other hand, analysis on more general stratified spaces is also possible including differential operators for corners with singularities allowed on the approach to lower dimensional strata (Schulze and Tarkhanov 2003).

7.4.4 Proofs of Propositions 1 and 2

Recall Proposition 1:

The product of two operators taking the form of Equation (15) can be rewritten as an signed-integer-weighted sum of expressions taking the same form. The product and the sum are equal, and graph-equivalent, and each is subpermutation-invariant with respect to indexing.

Proof: First we remark that the form of Equation (15), in which in which none of the indices $\langle i_1, ...i_k\rangle_{\neq}$ are allowed to be equal, is related by an invertible linear bijection with integer coefficients to sums of monomial basis operators (again with creation/annihilation exponents forced to be 0 or 1 since higher exponents result in zero operators) in which all combinations of index values $\{i_1, ...i_k\}$ are summed over. In the forward linear map from $\sum_{\{i_1,...i_k\}} \cdots$ to a sum of expressions of the form $\sum_{\langle i_1,...i_{k'\leqslant k}\rangle_{\neq}} \ldots$, an unrestricted sum over indices maps to a sum of index-equality-restricted sums over unequal indices. The leading order $k' = k$ term is unique and has the same summand in either form; all other terms have $k' < k$ and can be treated inductively. The sums over unequal indices that arise each have weight one and are in 1-1 correspondence with the partitions of k indices into groups that are constrained to be equal within a group and unequal to other groups, resulting in sums of sums each taking the form $\sum_{\langle j_1,...j_{k'\leqslant k}\rangle_{\neq}} \ldots$. Since there is only one partition of k into k different blocks, by recursion on k' from k down to 0 this linear map can

be inverted by successive equal-index substitutions $i_a \mapsto i_b$ where $i_a = i_b$, resulting again in integer coefficients.

Thus it suffices to prove the proposition for the related special form:

$$\begin{aligned} \hat{W}_r \propto \rho_r(\boldsymbol{\lambda}, \boldsymbol{\lambda}') \sum_{\{i_1,\dots i_k\}} & \left[\prod_{p',q' \in \mathrm{rhs}(r)} \left(\hat{a}_{i_{p'} i_{q'}} \right)^{g'_{p'\,q'}} \right] \left[\prod_{p' \in \mathrm{rhs}(r)} (\hat{a}_{i_{p'} \lambda'_{p'}})^{h'_{p'}} \right] \\ & \times \left[\prod_{p,q \in \mathrm{lhs}(r)} (a_{i_p i_q})^{g_{p\,q}} \right] \left[\prod_{p \in \mathrm{lhs}(r)} (a_{i_p \lambda_p})^{h_p} \right]. \end{aligned} \tag{42}$$

The product of two expressions of the form of Equation (15) initially takes the general form

$$\begin{aligned} \hat{W}_{r_2} \hat{W}_{r_1} \propto & \left(\rho_{r_1}(\boldsymbol{\lambda}_1, \boldsymbol{\lambda}'_1) \rho_{r_2}(\boldsymbol{\lambda}_2, \boldsymbol{\lambda}'_2) \right) \sum_{\{i_1,\dots i_{k_1}\}} \sum_{\{j_1,\dots j_{k_2}\}} \\ & \left[\prod_{p',q' \in \mathrm{rhs}(r_2)} \left(\hat{a}_{i_{p'} i_{q'}} \right)^{g'_{2,p'\,q'}} \right] \left[\prod_{p' \in \mathrm{rhs}(r_2)} (\hat{a}_{i_{p'} \lambda'_{2,p'}})^{h'_{2,p'}} \right] \\ & \times \left[\prod_{p,q \in \mathrm{lhs}(r_2)} (a_{i_p i_q})^{g_{2,p\,q}} \right] \left[\prod_{p \in \mathrm{lhs}(r_2)} (a_{i_p \lambda_{2,p}})^{h_{2,p}} \right] \\ & \times \left[\prod_{p',q' \in \mathrm{rhs}(r_1)} \left(\hat{a}_{j_{p'} j_{q'}} \right)^{g'_{1,p'\,q'}} \right] \left[\prod_{p' \in \mathrm{rhs}(r_1)} (\hat{a}_{j_{p'} \lambda'_{1,p'}})^{h_{1,p'}} \right] \\ & \times \left[\prod_{p,q \in \mathrm{lhs}(r_1)} (a_{j_p j_q})^{g_{1,p\,q}} \right] \left[\prod_{p \in \mathrm{lhs}(r_1)} (a_{j_p \lambda_{1,p}})^{h_{1,p}} \right], \end{aligned} \tag{43}$$

with each $g, h \in \{0, 1\}$. Recall that all $a, \hat{a}$ commutators are either zero, when operator types or indices don't match, or they are diagonal and a linear combination of the identity and a normal form $N = \hat{a}a$ matrix, multiplied by a delta function that eliminates one or more indices from the sum over indices.

We repeatedly commute factors of $\hat{a}$ on line 4 to the left of factors of a on line 3 until normal form - all creation operators to the left of all annihilation operators - is restored. Each out-of-order product $a_\alpha \hat{a}_\beta$ is replaced in turn using Equation (23d), which has several summands; distributing multiplication over addition at each step, each summand has the property of reducing the total (finite) number of out-of-order pairs by at least one pair; convergence to termination at a finite sum of operator terms generated by symbolic commutation, each in normal order, and each subpermutation invariant due to the sum over its remaining indices, is thus ensured by induction. Upon termination each elementary operator $\hat{a}_\alpha$ or a_α will appear linearly, to the power zero or one, in each summand, since $a_\alpha^2 = \mathbf{0} = \hat{a}_\alpha^2$ for all indices α; this fact eliminates summands that aren't multilinear (since all creation operators commute with each other and hence can be collected by subscript within the normal form, and likewise for annihilation operators.) Thus the final operator expression is

multilinear in these elementary operators, up to and including the final normal form. From that fact one can pick out appropriate 0/1-valued matrices $g_{1;2}$ and $g'_{1;2}$ on the edge labels, and 0/1-valued vectors $h_{1;2}$ and $h'_{1;2}$ on the vertices. Likewise with labelled vertices. Operator equality implies graph-equivalence. Subpermutation invariance of each normal order operator term, and therefore of both the starting product $\hat{W}_{r_2}\hat{W}_{r_1}$ and the ending sum of such terms, follows from the outer sum over all indices i_*, j_* that remain in each operator term, after some indices have been eliminated by Kronecker delta factors. QED.

Recall Proposition 2:

The product of two operators taking the form of Equation (16) can be rewritten as an signed-integer-weighted sum of expressions taking the same form. The product and the sum are equal, and graph-equivalent, and each is subpermutation-invariant with respect to indexing.

Proof:

As in the proof of Proposition 1, we first convert the sum over unequal indices into an integer-weighted sum of sums over arbitrary indices, of the following form:

$$\begin{aligned}\hat{W}_r \propto \rho_r(\boldsymbol{\lambda}, \boldsymbol{\lambda}') \sum_{\{i_1,\dots i_k\}} & \left[\Big(\prod_{p\in B_r} \prod_{i\neq i_q | \forall q \in \bar{B}_{rp}} E_{i_p\, i} \Big) \Big(\prod_{p\in C_r} \prod_{i\neq i_q | \forall q \in \bar{C}_{rp}} E_{i\, i_p} \Big) \right] \\ & \times \left[\prod_{p',q'\in \mathrm{rhs}(r)} \left(\hat{a}_{i_{p'} i_{q'}} \right)^{{g'}_{p'\,q'}} \right] \left[\prod_{p'\in \mathrm{rhs}(r)} \left(\hat{a}_{i_{p'} {\lambda'}_{p'}} \right)^{h_{p'}} \right] \\ & \times \left[\prod_{p,q\in \mathrm{lhs}(r)} \left(a_{i_p i_q} \right)^{g_{p\,q}} \right] \left[\prod_{p\in \mathrm{lhs}(r)} \left(a_{i_p \lambda_p} \right)^{h_p} \right]. \end{aligned} \tag{44}$$

By integer-weighted linearity we need only prove the Proposition for this form, rather than Equation (16).

Now the product of two expressions of the form of Equation (44) initially takes the general form:

$$
\begin{aligned}
\hat{W}_{r_2}\hat{W}_{r_1} \propto &\left(\rho_{r_1}(\boldsymbol{\lambda}_1,\boldsymbol{\lambda}_1')\rho_{r_2}(\boldsymbol{\lambda}_2,\boldsymbol{\lambda}_2')\right) \\
&\times \sum_{\{i_1,\ldots i_{k_1}\}} \sum_{\{j_1,\ldots j_{k_2}\}} \left[\Big(\prod_{p\in B_r'} \prod_{i\neq i_q|\forall q\in \bar{B}_{rp}'} E_{i_p\, i}\Big)\Big(\prod_{p\in C_r'} \prod_{i\neq i_q|\forall q\in \bar{C}_{rp}'} E_{i\, i_p}\Big)\right] \\
&\times \left[\prod_{p',q'\in \mathrm{rhs}(r_2)} \left(\hat{a}_{i_{p'} i_{q'}}\right)^{g'_{2,p'\,q'}}\right]\left[\prod_{p'\in \mathrm{rhs}(r_2)} \left(\hat{a}_{i_{p'}\lambda'_{2,p'}}\right)^{h'_{2,p'}}\right] \\
&\times \left[\prod_{p,q\in \mathrm{lhs}(r_2)} \left(a_{i_p i_q}\right)^{g_{2,p\,q}}\right]\left[\prod_{p\in \mathrm{lhs}(r_2)} \left(a_{i_p\lambda_{2,p}}\right)^{h_{2,p}}\right] \\
&\times \left[\Big(\prod_{p\in B_r} \prod_{i\neq i_q|\forall q\in \bar{B}_{rp}} E_{i_p\, i}\Big)\Big(\prod_{p\in C_r} \prod_{i\neq i_q|\forall q\in \bar{C}_{rp}} E_{i\, i_p}\Big)\right] \\
&\times \left[\prod_{p',q'\in \mathrm{rhs}(r_1)} \left(\hat{a}_{j_{p'} j_{q'}}\right)^{g'_{1,p'\,q'}}\right]\left[\prod_{p'\in \mathrm{rhs}(r_1)} \left(\hat{a}_{j_{p'}\lambda'_{1,p'}}\right)^{h_{1,p'}}\right] \\
&\times \left[\prod_{p,q\in \mathrm{lhs}(r_1)} \left(a_{j_p j_q}\right)^{g_{1,p\,q}}\right]\left[\prod_{p\in \mathrm{lhs}(r_1)} \left(a_{j_p\lambda_{1,p}}\right)^{h_{1,p}}\right].
\end{aligned}
\tag{45}
$$

This expression should equal a sum of expressions that take the same form as Equation (44), although possibly with negative signs and altered factors of E as stated, for various choices of graph rule matrices $g_{1;2v}$ and $g'_{1;2v}$ (v indexing the summands that result) and likewise for h. The leading factors of ρ multiply properly to give a new leading ρ for each summand, possibly to be multiplied by integers arising from commutation relations. The $\sum_{\{i_1,\ldots i_{k_1}\}} \sum_{\{j_1,\ldots j_{k_2}\}}$ will become a new $\sum_{\{i_1,\ldots i_{k_{1;2}\leqslant k_1+k_2}\}}$ after some nonnegative number of index collisions involving commutators proportional to $\delta_{i_{p_1} i_{p_2}}$ and $\delta_{j_{q_1} j_{q_2}}$ for various p and q subindices reduce the number of indices summed over by demanding index equality.

The proof work occurs in two steps: (1) commuting the post-factors of E in line 5 to join those in line 2, and (2) commuting factors of $\hat{a}$ from line 6 past (to the left of) factors of a in line 4 to restore normal form, more specifically for (2a) the edges and (2b) the node or vertex labels. We will discuss these two steps in reverse order, since Step 2 is simpler. Indeed, in the absence of E factors, the proof of the simplified form of Proposition 2 reduces to Proposition 1.

Recall from Equation (23) that all $a, \hat{a}$ commutators are either zero, when operator types or indices don't match, or they are diagonal and a linear combination of the identity and a normal form $N = \hat{a}a$ matrix, multiplied by a delta function that eliminates one or more indices from the sum.

Step 2. We repeatedly commute factors of $\hat{a}$ to the left of factors of a, as in the proof of Proposition 1, until normal form is reached. This determines the 0/1-valued entries of g, g', h, and h' for each summand.

Step 1. The products of E factors on line 5 of Equation (44) can commute freely to the left and upwards to line 1 except for possible interference with

$$\prod_{p',q' \in \mathrm{rhs}(r_2)} \left(\hat{a}_{i_{p'} i_{q'}}\right)^{g'_{2,p'\,q'}}$$

on line 3. Those E factors that get to line 1 either augment the sets B' and/or C' (possibly having lost some index values i to Kronecker delta functions with i_*, as recorded in sets $\bar{B}'$ and/or $\bar{C}'$) if they are new, or owing to $(E_\alpha)^2 = E_\alpha$ they make no change to line 1. To determine when E does not commute past line 3, we express $E_\alpha = I_\alpha - N_\alpha + a_\alpha = I_\alpha - \hat{a}_\alpha a_\alpha + a_\alpha$ (for $\alpha = (i_p\ i)$ or $(i\ i_p)$) and use the commutation relations Equation (23d), in which the commutation is the first summand and the second and third summands comprise a correction multiplied by a symbolic Kronecker delta function that enforces the equality of (in the case of edges) not just one but two index quantities i_* and/or i. So at least one of the sub-index quantities i_* will be removed by the Kronecker delta, along with its index summation, ensuring eventual termination in the normal form process as in Step 1 or the proof of Proposition 1. Thus, the obstructed terms arising from line 5 can be fully absorbed into the (annihilation-first) normal form factors that obstructed them, using the creation/annihilation commutators Equation (23d) to reduce all factors to normal form, thus joining in the normal form reduction process of Step 2. The result is a sum of operator expressions that are signed integer multiples of expressions in the form of Equation (44). Sub-permutation invariance and graph-equivalence are established as in the proof of Proposition 1. QED.

Propositions 1 and 2 are probably not the tightest or best formulations possible of the respective graph rewrite rule operator algebras, since they admit a wider class of operators than seems necessary (except in for purposes of the proof) and since they don't maintain as much control over the signs of the integer weights as seems possible in particular cases, (e.g. using Equation (23e) rather than (23d) to remove the explicit negative signs from the algebra by introducing matrix $Z_{i_p\, i_q}$ which has nonnegative entries). But they do show that graph rewrite rule semantics is embedded in an operator algebra in the manner specified, and in a way that could be computed automatically.

7.4.5 Model Reduction

The model reduction method introduced in Section 4.1 can be extended to the case of continuous spatial parameters (Equations (6) and (8)) as follows (Ernst et al. 2018): Instead of a discrete state vector $\boldsymbol{s}$ for all the numbers of all the possible (usually molecular) species, we have a representation comprising the total number $n : \mathbb{N}$ of objects (e.g. molecules) present, indexed by $i \in \{1, \ldots n\}$,

together with an n-dimensional vector $\boldsymbol{\alpha}$ of discrete species types α_i and an n-dimensional vector $\boldsymbol{x}$ of continuous spatial parameter vectors x_i such as $d = 3$ dimensions of space (though orientation could contribute $\binom{d}{2} = 3$ more components). Then a pure state vector (probability 1 concentrated on one state) can be denoted by the "ket" basis vector $|n, \boldsymbol{\alpha}, \boldsymbol{x}, t\rangle$, analogous to a single vector $|\boldsymbol{n}\rangle$ in GCCD above, and the full state of the system is a probability mixture of the basis states. Similar to GCCD, construct a coarse-scale mixed state based on an energy function and a Boltzmann distribution that sums over 1-particle contributions $\nu_1(\alpha_{i_1}, x_{i_1}, t)$ to the energy, summed over one particle index i_1, plus 2-particle contributions $\nu_2((\alpha_{i_1}, \alpha_{i_2}), (x_{i_1}, x_{i_2}), t)$ that obey permutation invariance, summed over two indices $i_1 < i_2$, and so on up to k-particle contributions of order $k = K$:

$$
\begin{aligned}
|[\nu_k|k \in \{1, \ldots K\}], t\rangle &= \sum_{n=0}^{\infty} \sum_{\boldsymbol{\alpha}} \int d\boldsymbol{x} \tilde{p}(n, \boldsymbol{\alpha}, \boldsymbol{x}, t) \, |n, \boldsymbol{\alpha}, \boldsymbol{x}, t\rangle \,, \\
&\qquad \text{with mixture probabilities } \tilde{p}(n, \boldsymbol{\alpha}, \boldsymbol{x}, t) : \\
\tilde{p} = \langle n, \boldsymbol{\alpha}, \boldsymbol{x}, t\rangle \, [\nu_k|k], t &= \frac{1}{\mathcal{Z}\,[[\nu_k|k]]} \exp[-\sum_{k=1}^{K} \sum_{\langle i\rangle_k^n} \nu_k(\boldsymbol{\alpha}_{\langle i\rangle_k^n}, \boldsymbol{x}_{\langle i\rangle_k^n}, t)]
\end{aligned}
\tag{46}
$$

where $\langle i\rangle_k^n = \{i_1 < i_2 < \cdots < i_k : i \in [1, n]\}$ denotes ordered subsets of k indexes each in $\{1, \ldots, n\}$. Here the partition functional $\mathcal{Z}\,[[\nu_k|k]]$ has two nested square brackets, the outer brackets indicating that $\mathcal{Z}[\ldots]$ depends on its arguments as a functional depends on functions rather than as a function depends on numbers, and the inner brackets indicating that the ν_k functions indexed by k should all be included in the argument list.

The goal as in Diagram 6 is to find $\tilde{p}$ that approximates the solution to the master equation $\dot{p}(n, \boldsymbol{\alpha}, \boldsymbol{x}, t) = W \cdot p(n, \boldsymbol{\alpha}, \boldsymbol{x}, t)$, where W sums over all processes that affect the state such as chemical reactions and diffusion (studied in Ernst et al. (2018)) but eventually also active transport, crowding effects and so on.

To define the time evolution of the reduced model $\tilde{p}(t)$, introduce a set of functionals $[\mathcal{F}_k|k \in \{1 \ldots K\}]$ (ODEs, PDEs, or even other forms for $\mathcal{F}$ could be tried) to create a governing dynamical system for the interaction functions $[\nu_k|k]$:

$$
\frac{d}{dt}\nu_k(\boldsymbol{\alpha}_{\langle i\rangle_k^n}, \boldsymbol{x}_{\langle i\rangle_k^n}, t) = \mathcal{F}_k\left[[\nu_{k'}(\boldsymbol{\alpha}, \boldsymbol{x}, t)|k']\right]. \tag{47}
$$

The criterion for choosing the functions $\mathcal{F}$ is to minimize an *action* that integrates a dissimilarity measure (KL-divergence) between p and $\tilde{p}$:

$$
\begin{aligned}
S &= \int_0^{\infty} dt \; \mathcal{D}_{\mathcal{KL}}(p||\tilde{p}), \qquad \text{where} \\
\mathcal{D}_{\mathcal{KL}}(p||\tilde{p}) &= \sum_{n=0}^{\infty} \sum_{\boldsymbol{\alpha}} \int d\boldsymbol{x} \; p(n, \boldsymbol{\alpha}, \boldsymbol{x}, t) \ln \frac{p(n, \boldsymbol{\alpha}, \boldsymbol{x}, t)}{\tilde{p}(n, \boldsymbol{\alpha}, \boldsymbol{x}, t)}.
\end{aligned}
\tag{48}
$$

In principle this minimization is a higher-order kind of variational calculus, in which one optimizes a higher order functional $S[\![\mathcal{F}]\!]$ of a functional $\mathcal{F}$ whose arguments are functions $\nu_k(\boldsymbol{\alpha}, \boldsymbol{x})$ of continuous space $\boldsymbol{x}$; in practice this particular problem is well approximated by reduction to a tractable algorithmic problem in terms of real-valued parameters by PDE-constrained optimization and a spatial mesh.

Assuming this objective has been minimized, (Ernst et al. 2018) use the chain rule of calculus to show:

Proposition 3 *Given a reaction network and a fixed collection of K interaction functions $\{\nu_k\}_{k=1}^K$, the linearity of the CME in reaction operators $\dot{p} = \sum_r W^{(r)} p$ extends to the functionals $\mathcal{F}_k = \sum_r \mathcal{F}_k^{(r)}$.*

In this very limited technical sense, there is a vector space homomorphism (which is a particular kind of structure-respecting map $\mathcal{R}$) from the spatial operator algebra semantics to the Dynamic Boltzmann reduced model semantics.

Speculation: Expression Dynamics for Model Search

One more significant connection between modeling languages and graph grammars is the possible use of graph grammars for structural model learning. Reaction and rewrite rule model classes share with artificial neural networks, Markov Random Fields, and many other machine-learnable model classes the property that model architecture is determined by a weighted graph (e.g. the bipartite graph of reactions and reactants) whose structure can be sculpted by setting nonzero weights (e.g. reaction rates) to zero or vice versa, with some expectation of continuity in the neighborhood of zero weight in an optimization formulation of training. This operation would correspond to the deletion or insertion of weak rules. However, other "structural" moves in graph space are bolder and may have higher potential optimization gain. For example if a rule is *duplicated* while maintaining the sum of the rates, then the model behavior and objective function are unchanged but at least one of the daughter rules may be freed up to drift and adopt new functions. Analogous "duplicate and drift" mechanisms are believed to enable neofunctionalization resulting in selective advantage in genetic evolution (Moore and Purugganan 2003; Thompson et al. 2016). Such an operation is easy to encode with a graph grammar meta-rule.

Duplication with arrow-reversal of one daughter rule, on the other hand, would require for continuity that the unreversed rule retain nearly its full strength and the reversed one enter the ruleset at very small strength. But as discussed in Section 2 such a reversed rule could subsequently evolve in strength to establish detailed balance. Other meta-rules could implement the mutation, crossover and three-parent rule-generation operations of genetic algorithms and differential evolution. Semantic word embeddings as used in current machine learning methods for natural language processing may provide a way to learn the inter-substitutability of individual symbols in rewrite rules

based on similarity of vectors evolved under previous successful substitutions, as has been done recently in the context of symbolic regression (Arabshahi et al. 2018). This kind of mechanism also promotes the evolution of evolvability. So in addition to the generic metarules we have listed, modeling-language specific or domain-specific rules could be optimized. All of these mechanisms for evolutionary structural optimization of models are local in the model AST and so can be encoded and studied using graph grammars.

Supplementary References

Note: Citations in the Supplementary Material text are either here, or in the main text References.

(Abelson et al. 1996) Harold Abelson and Gerald Jay Sussman Structure and Interpretation of Computer Programs - Second Edition MIT Press.

(Alnaes et al. 2014) Martin S. Alnaes, Anders Logg, Kristian B. Oelgaard, Marie E. Rognes, Garth N. Wells, "Unified Form Language: A domain-specific language for weak formulations of partial differential equations". ACM Transactions on Mathematical Software. Volume 40, Issue 2, Article No. 9, February 2014. Also arXiv:1211.4047 .

(Arabshahi et al. 2018) F. Arabshahi, S. Singh, A. Anandkumar. "Combining Symbolic Expressions and Black-box Function Evaluations for Training Neural Programs." Proc. International Conference on Learning Representations (ICLR). 2018

(Arnold et al. 2010) Douglas N. Arnold, Richard S. Falk, and Ragnar Winther, "Finite Element Exterior Calculus: From Hodge Theory to Numerical Stability". Bulletin of the American Mathematical Society. Volume 47, Number 2, pp. 281-354, April 2010.

(Backus 1978). J. Backus, "Can programming be liberated from the von Neumann style?: A functional style and its algebra of programs". Communications of the ACM. 21 (8): 613, 1978. doi:10.1145/359576.359579.

(Blinov et al. 2004) Michael L. Blinov, James R. Faeder, Byron Goldstein and William S. Hlavacek. BioNetGen: software for rule-based modeling of signal transduction based on the interactions of molecular domains. Bioinformatics, Vol. 20 no. 17, pages 3289-3291, 2004.

(Booth and Tillotson 1980) P. Booth and J. Tillotson, Monoidal closed, Cartesian closed and convenient categories of topological spaces. Pacific J. Math. Volume 88, Number 1 (1980), 35-53.

(Brown et al. 2008) R. Brown, I. Morris, J. Shrimpton and C.D. Wensley Graphs of morphisms of graphs the electronic journal of combinatorics 15 (2008), #A1

(Coifman and Lafon 2006) Coifman RR, Lafon S, "Diffusion maps". Appl Comput Harm Anal 21:5-30, 2006.

(de Goes et al. 2016) Fernando de Goes, Mathieu Desbrun, Mark Meyer, and Tony DeRose, "Subdivision Exterior Calculus for Geometry Processing". ACM Trans. Graph., 35(4), Art. 133, 2016.

(Desbrun et al 2005) Mathieu Desbrun, Anil N. Hirani, Melvin Leok, Jerrold E. Marsden, "Discrete Exterior Calculus". arXiv:math/0508341v2 , May 2002.

(Engwirda 2016) Darren Engwirda, "Conforming restricted Delaunay mesh generation for piecewise smooth complexes" 25th International Meshing Roundtable (IMR25) Procedia Engineering vol. 163 pp.84-96 (2016) doi:10.1016/j.proeng.2016.11.024

(Fages and Soliman 2008) Francois Fages and Sylvain Soliman 2008, "Abstract Interpretation and Types for Systems Biology". Theoretical Computer Science 403, 52-70, 2008.

(Fomin 1994) Serbey Fomin, Duality of Graded Graphs. Journal of Algebraic Combinatorics 3, 357-404, 1994

(Giavitto and Michel 2001) Jean-Louis Giavitto and Olivier Michel, "MGS: A Programming Language for the Transformations of Topological Collections". LaMI Technical Report 61-2001, Université d'Evry Val d'Essone May 2001.

(Giavitto and Spicher 2008) Jean-Louis Giavitto, Antoine Spicher "Topological rewriting and the geometrization of programming" Physica D 237 (2008) 1302-1314

(Glimm and Jaffe 1981) Glimm and Jaffe, *Quantum Physics: A Functional Integral Point of View.* Springer-Verlag 1981, Secrtion 6.1, Minkowski space axiom W3.

(Hammond et al. 2011) David K. Hammond, Pierre Vandergheynst, and Rémi Gribonval, "Wavelets on graphs via spectral graph theory". Applied and Computational Harmonic Analysis, Elsevier, 2011, 30 (2), pp.129-150.

(Hirsch 1976) Morris Hirsch, *Differential Topology*, Graduate Texts in Mathematics 33, Springer-Verlag New York, 1976.

(Holguera et al. 2018) Isabel Holguera and Claude Desplan, "Neuronal specification in space and time", Science 362, 176-180 (2018)

(HTT 2013) Homotopy Type Theory: Univalent Foundations of Mathematics 2013

(Hughes 2000) Thomeas J. R. Hughes, *Finite Element Methods.* Dover 2000.

(Imrich and Klavžar, 2000) Wilfried Imrich and Sandi Klavžar, *Product Graphs: Structure and Recognition.* John Wiley and Sons, 2000.

(Jones et al. 2008) Peter W. Jones, Mauro Maggioni, and Raanan Schul "Manifold parametrizations by eigenfunctions of the Laplacian and heat kernels" Proc. Nat. Acad. Science USA, vol. 105 no. 6 1803-1808, 2008

(McQuarrie 1967) Donald A. McQuarrie "Stochastic Approach to Chemical Kinetics". Journal of Applied Probability, Vol. 4, No. 3 (Dec., 1967), pp. 413-478

(Kipf and Welling 2016) Thomas N. Kipf, Max Welling, "Semi-Supervised Classification with Graph Convolutional Networks". arXiv:1609.02907.

(Knauer 2011) Ulrich Knauer Algebraic Graph Theory: Morphisms, Monoids and Matrices DeGruyter, Sept. 2011 978-3-11-025509-6 Section 4.3, Theorem 4.3.5.

(Lane 2015) Brendan Lane, "Cell Complexes: The Structure of Space and the Mathematics of Modularity"', PhD thesis, University of Calgary, September 2015.

(Logg et al. 2012) Anders Logg and Kent-Andre Mardal and Garth N. Wells and others, *Automated Solution of Differential Equations by the Finite Element Method.* Springer, 2012.

(Mandl and Shaw 2010) Mandl and Shaw, Quantum Field Theory, 2nd Edition. J. Wiley. Section 13.2, requiring a Wick rotation $t \to \pm it$ to relate quantum and statistical field theories.

(Martinelli et al 1982) A. Martelli and U. Montanari, "An Efficient Unification Algorithm". ACM Transactions on Programming Languages and Systems,Vol.4, No. 2, April 1982,Pages 258-282. April 1982.

(Milnor 1980) Robin Milner, *A Calculus of Communicating Systems*, Springer Verlag,1980.

(McGrew et al. 2018) McGrew, W. F., Zhang, X.,Fasano, R. J., Schffer, S. A., Beloy, K., Nicolodi, D. , Brown, R. C., Hinkley, N., Milani, G., Schioppo, M. and Yoon, T. H., and Ludlow, A. D., "Atomic clock performance enabling geodesy below the centimetre level". Nature 564(7734), pp. 87-90, 2018.

(Mjolsness and Prasad 2013) Eric Mjolsness and Upendra Prasad, "Mathematics of Small Stochastic Reaction Networks: A Boundary Layer Theory for Eigenstate Analysis". Journal of Chemical Physics 138, 104111 (DOI: 10.1063/1.4794128), March 2013.

(Moore and Purugganan 2003) Richard C. Moore and Michael D. Purugganan, "The early stages of duplicate gene evolution". Proceedings of the National Academy of the United States of America, 100 (26) 15682-15687; December 23, 2003. https://doi.org/10.1073/pnas.2535513100

(Murphy et al. 2001) Michael Murphy, David M. Mount, and Carl W. Gable, "A point-placement strategy for conforming Delaunay tetrahedralization". International Journal of Computational Geometry and Applications Vol. 11, No. 6 pp. 669-682, 2001.?

(Nedelec et al. 2007) Francois Nedelec and Dietrich Foethke "Collective Langevin dynamics of flexible cytoskeletal fibers" New J. Phys. 9 427, 2007.

(Pierce 2002) Benjamin C. Pierce, *Types and Programming Languages.* MIT Press 2002.

(Plotkin 2004) Gordon D. Plotkin. The Origins of Structural Operational Semantics. The Journal of Logic and Algebraic Programming. 60-61:3-15, 2004.

(Poggio and Girosi 1990) T. Poggio; F. Girosi "Regularization Algorithms for Learning that are Equivalent to Multilayer Networks" Science, New Series, Vol. 247, No. 4945. , pp. 978-982, Feb. 23, 1990.

(Prusinkiewicz and Lindenmeyer 1990) Przemyslaw Prusinkiewicz and Aristed Lindenmeyer *Algorithmic Beauty of Plants* Springer-Verlag 1990 .

(Rand and Walkington 2009) Alexander Rand and Noel Walkington "Collars and Intestines: Practical Conforming Delaunay Refinement" In: Clark B.W. (eds) Proceedings of the 18th International Meshing Roundtable. Springer, Berlin, Heidelberg 2009. https://doi.org/10.1007/978-3-642-04319-2_28

(Schulze and Tarkhanov 2003) B.-W. Schulze N. Tarkhanov "Differential analysis on stratified spaces" In: *Hyperbolic Differential Operators and Related Problems* (Eds. Ancona and J. Vaillant), Lecture Notes in Pure and Appl. Math., vol. 233, Marcel Dekker, New York, pp. 157-178, 2003.

(Scott and Mjolsness 2019) C.B. Scott and Eric Mjolsness, Multilevel Artificial Neural Network Training for Spatially Correlated Learning. SIAM Journal on Scientific Computing, to appear. Also arXiv preprint arXiv:1806.05703, 2018; URL: http://arxiv.org/abs/1806.05703.

(Shapiro et al. 2015a) Bruce E. Shapiro, Cory Tobin, Eric Mjolsness, and Elliot M. Meyerowitz, "Analysis of Cell Divisions Patterns in the Arabidopsis Shoot Apical Meristem", Proceedings of the National Academy of Sciences 112:15 pp 4815-4820, 2015.

(Si and Gartner 2005) Si H. and Gartner K., Meshing Piecewise Linear Complexes by Constrained Delaunay Tetrahedralizations, Proceeding of the 14th International Meshing Roundtable, September 2005.

(Spicher et al. 2007) Antoine Spicher and Olivier Michel, "Declarative modeling of a neurulation-like process". Bio Systems, vol 87 2-3, pp. 281-8, 2007.

(Steenrod 1967) N. E. Steenrod, "A convenient category of topological spaces". Michigan Mathematical Journal, Volume 14, Issue 2, 133-152, 1967.

(Thompson et al. 2016) Ammon Thompson, Harold H. Zakon, and Mark Kirkpatrick "Compensatory Drift and the Evolutionary Dynamics of Dosage-Sensitive Duplicate Genes" Genetics, vol. 202 no. 2 765-774; February 1, 2016 . https://doi.org/10.1534/genetics.115.178137

(Tindemans et al. 2014) Simon H. Tindemans, Eva E. Deinum, Jelmer J. Lindeboom and Bela M. Mulder, "Efficient event-driven simulations shed new light on microtubule organization in the plant cortical array" Frontiers in Physics, v. 2 Article 19, April 1014. doi: 10.3389/fphy.2014.00019

(Van Kampen 1981) van Kampen, N. G.: *Stochastic Processes in Physics and Chemistry.* North-Holland (1981)

(Weinberger 1994) S. Weinberger, *The Topological Classification of Stratified Spaces*, University of Chicago Press, 1994.

(Wightman and Turner 2007) R. Wightman, S.R. Turner. "Severing at sites of microtubule crossover contributes to microtubule alignment in cortical arrays". The Plant Journal 52:742-751, 2007.

(Wolpert 1969) L. Wolpert, "Positional information and the spatial pattern of cellular differentiation". J. Theoretical Biology 25 (1): 147, 1969.
